# Multi-resolution tone mapping for high dynamic range medical ultrasound images

**DOI:** 10.1371/journal.pone.0340777

**Published:** 2026-01-20

**Authors:** Thi Lan Nhi Vu, Vimal Chandran, Christina Haberl, Otmar Scherzer, Julia Binder

**Affiliations:** 1 Faculty of Mathematics, University of Vienna, Vienna, Austria; 2 Christian Doppler Laboratory for Mathematical Modeling and Simulation of Next Generations of Ultrasound Devices (MaMSi), Vienna, Austria; 3 GE Healthcare, Zipf, Austria; 4 Department of Obstetrics and Gynecology, Division of Obstetrics and Fetomaternal Medicine, Medical University of Vienna, Vienna, Austria; 5 Johann Radon Institute for Computational and Applied Mathematics (RICAM), Linz, Austria; Islamia University of Bahawalpur: The Islamia University of Bahawalpur Pakistan, PAKISTAN

## Abstract

Ultrasound diagnostics is a key tool in obstetrics for detecting fetal anomalies and monitoring pregnancy, but image quality often declines in obese patients due to reduced contrast resolution. This pilot study develops and preliminarily validates a novel tone-mapping algorithm for enhancing contrast resolution in high dynamic range (HDR) ultrasound images. The method employs multi-resolution fusion with depth-adaptive weighting and depth compensation to improve contrast, enhance tissue differentiation, reduce noise, and preserve fine details. The algorithm was tested on 20 fetal ultrasound images focused on fetal kidney visualization. Quantitative evaluation showed a 5.4% mean increase in entropy and mean generalized contrast-to-noise ratio (gCNR) improvements of 15.79%, 8.93%, and 17.39% between fetal kidneys and amniotic fluid, far-field objects and fluid, and fetal kidneys and adjacent tissues, respectively, compared with an existing method. These results demonstrate improved anatomical visualization, particularly of fetal kidneys, with potential clinical relevance.

## Introduction

Ultrasound diagnostics has become a standard tool in obstetrics, widely used for detecting fetal anomalies and monitoring pregnancy progress. It offers the advantages of real-time image acquisition at high frame rates and the absence of radiation exposure [3]. However, research indicates that in obese individuals, the quality of abdominal ultrasound may be compromised, making it more challenging to visualize and assess deeper anatomical structures [4]. Given the global rise in obesity [5], addressing these challenges has become increasingly important.

A critical component of ultrasound image quality affected in patients with a high fatty-layer thickness is contrast resolution [6,7], which is essential for clearly differentiation between soft tissues, fluids, and uterine structures, aiding in the identification and evaluation of fetal organs. Increased adipose tissue in these patients leads to greater attenuation of ultrasound waves due to enhanced absorption and scattering [8]. As a result, the signal strength weakens with depth, lowering contrast resolution and making it more difficult to distinguish structures in deeper regions of the body. This challenge is particularly evident when imaging low impedance structures, such as the fetal kidneys, which are positioned deep within the uterus in obese patients. Improving contrast resolution in these cases is vital to enhance visibility, enable early detection of important anatomical details, and support timely diagnosis of pregnancy-related complications.

An approach to enhance contrast resolution in ultrasound images is high dynamic range (HDR) image processing. This technology, widely used in fields such as photography and computer graphics, improves visual experience by capturing a broader range of tonal details [9–11]. HDR imaging consists of two main steps: first, combining multiple images taken at different exposures to capture both extremely bright and dark regions; second, compressing the HDR image to fit the limited dynamic range of standard displays or human vision [12]. Without this second step, known as *tone mapping*, extreme brightness differences can cause loss of important details in both bright and dark areas.

In the case of ultrasound imaging, only the tone mapping step is required, as raw ultrasound data already possesses an extremely high dynamic range [13]. For instance, raw data acquired from a modern ultrasound scanner (VOLUSON Expert 22, GE Healthcare) can span a range far beyond what standard screens can display [15]. The main challenge is therefore in compressing this broad range of information so that subtle tissue differences remain visible. Doing this effectively is essential for clinical interpretation, as it can help reveal anatomical details that might otherwise be hidden.

In clinical practice, standard B-mode ultrasound uses logarithmic compression to reduce the dynamic range for display. While this approach is simple and effective in scaling the signal range, it presents several limitations that may impact clinical utility [7,13]. One key limitation is the uneven compression across intensity levels. Higher intensity signals, often corresponding to hyperechoic tissues, are disproportionately compressed, leading to loss of structural detail [13,16,33]. Conversely, hypoechoic tissues, which inherently produce weaker echoes, may appear underexposed and difficult to interpret [17]. Furthermore, logarithmic compression tends to flatten local contrast, reducing subtle texture differences and making it harder to distinguish adjacent tissues [13,16,19]. These issues highlight the need for a more sophisticated, ultrasound-specific tone mapping operator (TMO) that can preserve critical anatomical detail while enhancing contrast resolution.

Although tone mapping has been extensively explored in photography and computer vision to balance local contrast and global illumination, with techniques ranging from simple global mapping techniques [18–22] to more complex adaptive local operators [23–32], its application to ultrasound imaging remains limited. Prior studies on high dynamic range ultrasound (HDR-US) have applied popular tone mapping algorithms to compare with conventional US but have not sufficiently addressed ultrasound-specific challenges such as speckle noise and signal attenuation with depth [13,14]. These challenges are particularly critical in fetal ultrasound scanning of obese patients, where increased adipose tissue thickness positions the fetus deeper, resulting in greater signal attenuation in distal regions. This necessitates a dedicated, depth-aware tone mapping technique for ultrasound.

This study aims to develop and validate an ultrasound-specific tone mapping algorithm that accounts for depth-dependent signal attenuation, particularly in fetal ultrasound of obese patients. We hypothesize that this algorithm will improve the visualization of fetal kidneys and other deep structures while enhancing tissue differentiation compared with conventional techniques.

The paper is organized as follows: the Related Works section reviews prior studies. The Methodology section presents the proposed approach, followed by Materials and Experiments. The Results section presents the quantitative findings, and the Discussion interprets them. Finally, the Conclusion summarizes the contributions and outlines future work.

## Related works

Since tone mapping research began in the 1990s, many tone mapping operators (TMOs) have been developed based on criteria such as local contrast reproduction, detail preservation, and models of human vision. In this section, we provide a concise technical overview of traditional TMOs from photography and computer vision, establishing the foundation for understanding their principles and setting the stage for evaluating their applicability to ultrasound imaging in a later section.

**Fig 1:**
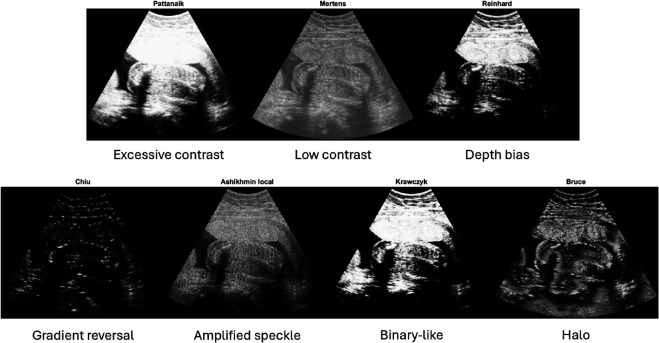
Examples of common failure modes observed in tone-mapped ultrasound images. **Row 1:** *1. Excessive contrast* - Some regions are overexposed (too bright), resulting in overly enhanced contrast and loss of subtle details. *2. Low contrast* - the image appears flat, lacking true black and white regions. *3. Depth bias* - intensity distribution varies unevenly with depth (e.g. overexposed near-field and underexposed far-field). **Row 2:** *1. Gradient reversal* - artifacts appear along object boundaries. *2. Amplified speckle* - speckle noise becomes more pronounced. *3. Binary-like appearance* - image exhibits only black and white regions with limited grayscale tone. *4. Halo arifacts* - bright halo or glow appear around objects boundaries.

To motivate this evaluation and provide context, we present a summary of the discussed TMOs in Table 1, categorized by type, along with their publication year, computational time, and an overview of challenges when applied to the ultrasound domain. Visualization examples illustrating specific failure modes are presented in Fig 1. A detailed comparative analysis of these methods is provided in the Results and Discussion section, and visual results are included in the Appendix.

**Table 1:** Tone mapping operators grouped by types, with publication years, average computational time with standard deviation and limitations in ultrasound domain.

Type	TMO	Year	Time (s)	Limitations
global	Pattanaik [18]	2000	0.12 ± 0.01	excessive contrast
	Drago [19]	2003	0.02 ± 0.00	excessive contrast, binary-like appearance, depth bias
	Kim [20]	2008	0.03 ± 0.00	excessive contrast, depth bias
	Khan18 [21]	2018	0.30 ± 0.04	low contrast
	Khan20 [22]	2020	0.08 ± 0.01	low contrast
local	Chiu [23]	1993	0.38 ± 0.03	gradient reversal artifacts
	Reinhard [25]	2002	0.05 ± 0.01	excessive contrast, binary-like appearance, depth bias
	Ashikhmin [26]	2002	0.31 ± 0.01	amplified speckle artifacts
	Madmad [38]	2003	6.457 ± 0.75	excessive contrast, binary-like appearance, depth-bias, time-intensive
frequency-based	TumblinTurk [24]	1999	0.02 ± 0.00	excessive contrast, binary-like appearance, depth bias
	Durand [27]	2002	0.07 ± 0.02	low contrast
	Liang [32]	2018	1.55 ± 0.06	low contrast
segmentation-based	YeePattanaik [28]	2003	49.48 ± 3.29	amplified speckle artifacts, excessive contrast, time-intensive
	Krawczyk [29]	2005	1.49 ± 0.19	excessive contrast, binary-like appearance
exposure fusion	Mertens [30]	2007	0.78 ± 0.03	low contrast
	Bruce [31]	2014	6.17 ± 0.36	amplified speckle artifacts, halo artifacts, time-intensive

### Global tone mapping operators

TMOs that are applied pixelwise to the entire image are classified as global. The most straightforward approach is to use a single tone curve on the dynamic range of an HDR image. Some basic transformation are logarithmic, exponential and linear scaling. Over the years, more complex global tone mapping models have been developed, taking the human visual system (HVS) into account.

- One of the earliest models that considers the HVS was proposed by Pattanaik et al. in 2000 [18]. In this model, luminance values, the retinal responses of rods and cones, and the color processing mechanisms in human vision are integrated into the tone curve.
- Another approach, developed by Drago et al. [19], adaptively adjusts the logarithmic bases to logarithmically compress the dynamic range. This method enhances the brightness of dark pixels using smaller logarithmic bases and compresses brighter pixels with larger bases.
- Kim et al. [20] proposed a non-linear scaling factor for adjusting the dynamic range, based on HVS sensitivity in the logarithmic domain. This method achieved better performance compared to using a linear scaling factor.
- Years later, Khan et al. [21] introduced a histogram-equalization method that involved constructing a histogram with bins uniformly spaced according to the HVS. Pixels from the histogram bins that are indistinguishable to the HVS are eliminated, and the remaining bins are redistributed proportionally based on pixel counts. In [22], they embedded a perceptual quantizer, a non-linear transfer function based on human visual perception of banding, into the histogram-based approach. when applied to HDR-US data.

### Local tone mapping operators

In contrast to global TMOs, local TMOs adjust each pixel according to the information from its neighboring pixels.

- One of the first operators of this category was introduced by Chiu et al. [23], employing spatially nonuniform mappings to scale the luminance values. In his model, the scaling map used to calculate the local mean of the surrounding pixels is indirect proportional to the blurred version of the original image. The scaling factor can be smoothed again by a low-pass filter to avoid artifacts.
- Reinhard et al. [25] adopted an alternative approach by incorporating photography techniques into the compression process. Their method, inspired by Ansel Adams’ zone system [1], simulates the dodging and burning effects applied in photography print. To retain the maximum contrast, a dark pixel in a relatively bright region will be compressed more, resulting in darkened bright regions, whereas a bright pixel in a relatively dark region will be compressed less, thus brightens up this dark region.
- Another approach using photographic mechanism was presented by Ashikhmin [26]. His TMO takes functionality of HVS into account and addresses two primary objectives: maintaining local contrast and preserving visual contrast. The latter is calculated by a simplified threshold versus intensity function, which describes perceptual capacities of world and display. Based on the pixels’ relative positions in corresponding perceptual scales, image luminance is compressed, ensuring the perceptual uniformity.
- Histogram-based methods were further integrated into locally adaptive tone mapping. Madmad et al. [38] performed histogram equalization using a bilateral weight map to allocate pixels to predefined bins. The weights for each pixel are based on its spatial neighborhood and the diffusion properties of a cross-bilateral filter.

### Frequency-based tone mapping operators

Apart from taking spatial information into account, dynamic range compression can also be performed in the frequency domain. An image can be decomposed into low frequencies (large features) and high frequencies (details) using a filter. To preserve edges and local contrast, low frequencies are often compressed strongly, whereas the detail layer are preserved or even enhanced.

- Tumblin and Turk [24] integrated this concept into tone mapping by using a multi-scale low curvature image simplifier (LCIS) filter. By compressing only the lowest frequencies, the method preserves high-frequency components and local contrast.
- A popular non-linear filter that separate an image into a high frequency and a low frequency image is bilateral filter. Influenced by Tumblin and Turk’s work, Durand and Dorsey [27] used this filter in their tone mapping approach to preserve local contrast, but with a single decomposition step rather than a multi-scale approach, resulting in a more efficient model. The base layer is scaled scaling in the logarithmic domain, while the detail layer remains unchanged.
- Liang et al. [32] address the issues of halo artifacts and over-enhancement present in existing frequency-based TMOs by proposing a hybrid $\ell_{1}\;-\;\ell_{0}$ decomposition model. $\ell_{1}$ and $\ell_{0}$ gradient sparsity terms are optimized and used on the base and detail layer, respectively, to smooth large features while keeping the edges undisturbed.

### Segmentation-based tone mapping operators

Since strong edges and local contrast are typically found at the boundaries of large homogeneous areas, another method to preserve details is dividing the image into uniform segments, applying a global TMO to each segment, and then recombining them.

- Yee and Pattanaik [28] introduced the idea of a segmentation-based TMO. They proposed a method for computing local adaptation luminance that can be used with several different TMOs. In their algorithm, HDR images are partitioned into different regions based on the image histogram in the logarithmic domain. For each region, the adaptation luminance is calculated and then tone-mapped globally.
- Krawczyk [29] integrated Gilchrist et al.’s anchoring theory of lightness perception [2] into a segmentation-based TMO. According to this theory, the highest luminance value in a relative area, known as the anchor, is perceived as white by the HVS. In Krawczyk’s model, the image is segmented into regions of consistent luminance where this anchoring can be applied. Each segment is then tone-mapped and merged proportionally to its strength.

### Exposure fusion

Exposure fusion is not traditionally classified as a tone mapping method, but rather an alternative approach to combining LDR images with different exposures. In conventional photography workflows, a series of LDR images of the same scene, each captured at different exposure settings, is first combined into a single HDR image. This HDR image is then compressed into an 8-bit format using a tone mapping algorithm. Exposure fusion is often used in photography, as images with varying exposures are typically available before being combined into an HDR image. By applying exposure fusion directly, the compression step can be bypassed.

Despite its primary role as an alternative to tone mapping, exposure fusion can also be adapted to function as a TMO. Specifically, given an HDR image, one can synthesize multiple versions at different exposure levels (for example, through segmentation or intensity scaling techniques) and then apply exposure fusion to combine these representations into a final compressed image. This approach effectively leverages the principles of exposure fusion to achieve dynamic range compression.

- Mertens et al. [30] proposed an innovative method that integrates well-exposed pixels from each exposure into a single LDR image. Their model blends a series of LDR images using a multi-resolution approach to preserve details. Instead of averaging the intensity values of all the images to determine the intensity of the new image, Mertens’ algorithm calculates the weighted average of each images’ Laplacian pyramid [34]. The weights are derived from three key metrics: contrast, saturation, and well-exposedness. Contrast prioritizes regions with high local variation, which are visually distinct and contribute to sharper details in the final image. Saturation enhances vivid and vibrant colors, ensuring the resulting image appears visually rich and natural. Well-exposedness emphasizes pixels with mid-range intensities, avoiding underexposed (too dark) nor overexposed (too bright) regions, making these pixels visually optimal. By blending details rather than raw intensity values, the method effectively integrates the most visually significant features from each input image.
- Inspired by the effectiveness of this operator, Bruce [31] later introduced an information maximization fusion strategy that leverages relative entropy across exposures. The algorithm computes local entropy for each exposure after logarithmic intensity compression and normalizes entropy across exposures. This enables a fusion strategy based on relative entropy combined with a soft-maximum operation. Additionally, Bruce’s method introduces a single parameter to balance the trade-off between illumination and fine detail in the final result.

One one hand, traditional tone-mapping techniques have proven effective in natural and photographic imaging, but their direct application to ultrasound can be limited by domain-specific characteristics such as speckle noise and signal attenuation. In particular, traditional TMOs can exhibit failure modes such as gradient reversal or amplified speckle because they do not account for speckle noise. Similarly, excessive contrast, depth-dependent bias, or a binary-like appearance can lead to significant information loss, as these methods do not consider the depth-dependent nature of ultrasound signals. This is especially critical because effective tone-mapping requires retaining as much information as possible during compression.

On the other hand, existing ultrasound enhancement methods, including contrast improvement via histogram equalization [39] and speckle reduction through multiscale filtering [40], have been primarily developed for standard B-mode imaging. While these approaches address some limitations of traditional tone-mapping, they are not specifically designed for HDR data and may fail to preserve details across the full intensity range during HDR-to-LDR compression. In contrast, methods from other domains that require careful information preservation have proven effective in capturing both fine details and broader structural patterns by combining information from multiple sources, analogous to using different intensity intervals in HDR data [41]. Adaptive weighting mechanisms can further enhance performance by adjusting the contribution of each feature according to its informativeness and reliability within each intensity range [42]. Motivated by these complementary strengths, the proposed method seeks to integrate advantages from different domains for application to HDR ultrasound.

## Methodology

In this section, we present a novel tone-mapping technique to compress the wide dynamic range of HDR ultrasound data, which can reach up to 65 dB (or ∼11 bits) with the Voluson Expert 22 (Probe C2-9) [15], into the much narrower dynamic range supported by standard ultrasound displays, typically 8 bits [15]. The goal is to enhance contrast resolution while preserving critical details in the compressed B-mode images, particularly for challenging cases such as imaging in obese patients.

To address these challenges, we propose an algorithm that integrates depth-compensation, multi-resolution processing, and optimized weighted fusion specifically tailored for ultrasound imaging. First, the HDR-US image undergoes depth compensation by progressively enhancing image intensity in deeper regions,addressing signal attenuation unique to ultrasound. The depth-compensated data is then partitioned into regions with similar intensity levels based on its histogram, enables adaptive processing of the HDR-US data. This novel component leverages the fact that different intensity levels correspond to distinct tissue types, such as hyperechoic and hypoechoic regions [17], enabling targeted processing that enhances contrast resolution between these tissues.

To further address the challenges of ultrasound imaging, where subtle anatomical structures are often difficult to distinguish due to complex tissue patterns and intensity variations, we employ a multi-resolution approach. By capturing both coarse and fine details, this method enables analysis and enhancement at multiple scales, improving subtle features in intricate regions without compromising the overall structure of the image.

We then adapt the weighted Mertens’ multi-resolution fusion strategy by making the fusion weights explicitly adaptive to image depth. Rather than relying solely on conventional measures such as contrast and well-exposedness, which are inherently affected by depth-dependent signal attenuation in ultrasound, we dynamically adjust the fusion weights based on the local depth of each region. This approach accounts for the natural weakening of ultrasound signals with depth due to absorption, scattering, and beam divergence, which makes the far-field more susceptible to noise. While depth compensation helps balance signal levels across the field of view, it can also amplify noise in the far-field, where noise and object details are often closely intertwined. By incorporating depth-adaptive weighting, noise is suppressed while critical object details are preserved, particularly in deeper tissues or in patients with thicker adipose tissue.

**Fig 2:**
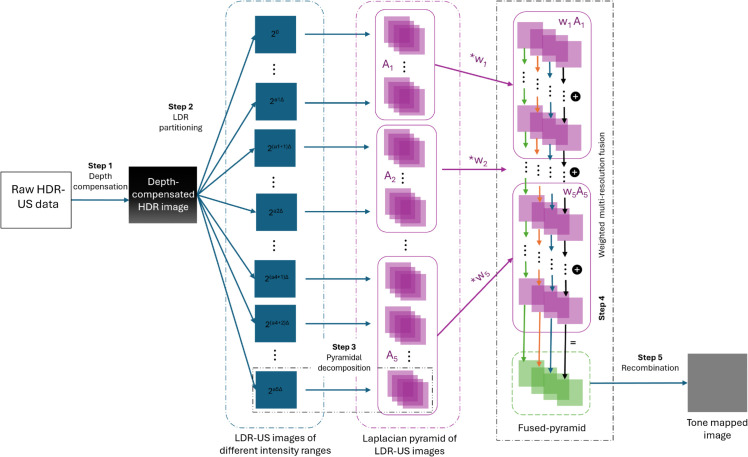
Overview of the presented multi-resolution fusion-based tone mapping. Step 1: raw HDR-US data is depth-compensated. Step 2: depth-compensated HDR-US data is then converted into a series of LDR-US images of different intensity ranges. Step 3: for each image in the LDR-series, a Laplacian pyramid is constructed. Step 4: the pyramids are fused into one using a weighted multi-resolution approach. Step 5: The fused pyramid is recombined into the final tone-mapped image.

The complete workflow of our algorithm is illustrated in Fig 2 and is explained in detail in the following subsections. It consists of 5 steps:

(1) **Our depth compensation model**.
(2) **Our method of partitioning HDR data** into LDR images of different intensity ranges
(3) Pyramidal decomposition, a well-established technique from [34].
(4) **Our new weighting scheme** applied to Mertens’ weighted multi-resolution fusion [30].
(5) Recombining the pyramid into an image, as in Mertens’ technique [30].

### Step 1: Depth compensation

In ultrasound imaging, the area closest to the transducer is called the near-field, while the region beyond the transducer’s focal zone is known as the far-field. The intermediate zone between these regions is referred to as the mid-field. Based on the acquisition setting [15], we define the near-field as ranging from 0 cm to 4.5 cm, the mid-field from 4.5 cm to 10 cm, and the far-field as starting at 10 cm.

In fetal ultrasound scans, the structures typically lie in the range of 4.7 to 13.9 cm [35], depending on gestational age, maternal body habitus, and the specific anatomy being examined. This depth corresponds to the mid-field and far-field regions, where fetal organs such as the kidneys, heart, and brain are commonly visualized (for instance, see Fig 12).

In individuals with obesity, however, the increased thickness of abdominal adipose tissue significantly alters the imaging depth. Maternal abdominal wall thickness, defined as the maximum subcutaneous tissue thickness from the skin surface to the uterine serosa, ranges from 1.2–3.46 cm in patients with a normal BMI but increases to 1.67–6.82 cm in those with obesity [45]. This additional adiposity displaces the uterus and fetal structures further posteriorly, often pushing them into the far-field, at depths exceeding 10 cm. Consequently, the mean depth of insonation required for imaging in obese individuals is significantly greater [6,46].

**Fig 3:**
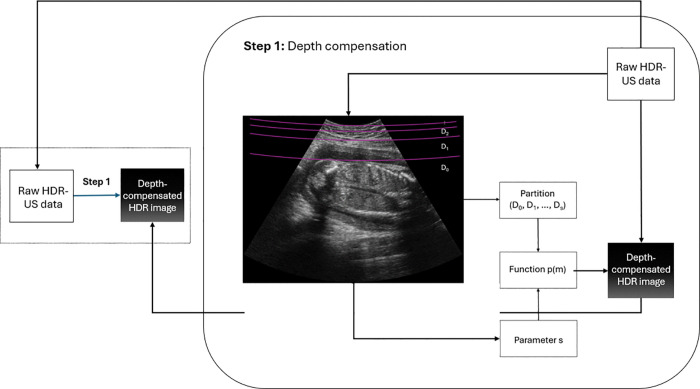
Overview of the depth compensation process. Step 1: Determine the location of the fatty layer and fetal position. Step 2: Choose an appropriate parameter *s*. Step 3: Partition the image into regions D(0),D(1),⋯,D(s). Step 4: Compute the function *p* based on the partitioning. Step 5: Multiply the raw HDR-US data by the function *p*.

This increased depth presents unique challenges in ultrasound imaging for obese patients. The thicker adipose tissue causes significant signal attenuation, which, combined with the natural decrease in ultrasound signal strength with depth due to absorption, scattering, and beam divergence, reduces contrast and makes it particularly challenging to visualize details in the far-field [6]. This necessitates additional compensation in the far-field to maintain maintain uniform contrast and detail throughout the image, enabling better visualization of structures in both the near-field and far-field. The workflow for this depth-compensation step is illustrated in Fig 3.

Formally, let the initial HDR-US data be denoted by


I:Ω:={1,2,...,M}×{1,2,...,N}→[0,∞).

Here, *M* and *N* are the discretizations of depth and radial components, respectively. The depth-compensated data is defined as

$$\hat{I}(m,n)=p(m)I(m,n)\;\forall (m,n)\in \Omega .$$ (1)

Depth compensation balances energy levels across different depths by amplifying signals from deeper regions to match those from shallower regions. Therefore, the function *p* must grow progressively with increasing depth.

Defining *p* as an exponential function is undesirable, as ultrasound scans often support larger depths [15]. Amplifying the intensity of the original HDR data using an exponential function not only results in an excessively large dynamic range that requires compression but also risks numerical overflow, as the values may exceed the limits of standard floating-point representations.

This issue can be partially addressed by approximating the exponential with its Taylor polynomial [47]. However, when a fixed-degree polynomial is used, amplification varies significantly with depth, amplifying far-field signals substantially while only slightly increasing near-field intensities. As a result, after compression, the near-field details are diminished and often lost. To address this, we aim to define a function *p* whose rate of increases adapts across the near-, mid-, and far-field regions. It should approximate exponential growth in the near-field while growing monotonically with a diminishing rate as depth increases. This approach ensures that signals from different regions are amplified appropriately, maintaining uniform contrast throughout the image.

To address this, we partition the ultrasound image into different layers and increase the polynomial degree, which corresponds to the growth rate, as the layer approaches the near-field. We set both the number of partitions and the lowest polynomial degree, corresponding to the region farthest from the transducer, to the same value, denoted by *s*, in order to reduce the number of parameters and simplify the model. This parameter *s* is determined heuristically.

As the result, the piecewise polynomial function *p* is defined formally as the following:

$$p(m)=\sum_{i=0}^{s}p_{i}(m)\;1_{D(i)}(m),$$ (2)

where *D*(*i*) for i=0,⋯,s are the partitions of the image depth, defined by


$$D(s)=\left[0,\frac{5M}{2^{s}s}\right],\;D(i)=\left(\frac{5M}{2^{i+1}s},\frac{5M}{2^{i}s}\right]\text{ for }i=1,\cdots ,s-1,\;D(0)=\left(\frac{5M}{2s},M\right],$$

$1_{A}$ is the indicator function of a set *A* and *p*_*i*_ is the (i+s)-th order approximation of the exponential function, that is, the order of *p* increases by 1 as *i* increases by 1,

$$p_{i}(m)=\left\{ \begin{array}{cc} \sum_{j=1}^{i+s}\frac{1}{j!}m^{j} & \text{ for }i=s\\ p_{i+1}(\frac{5M}{2^{i+1}s})+\sum_{j=1}^{i+s}\frac{1}{j!}m^{j} & \text{ for }i=0,\cdots ,s-1. \end{array}\right..$$ (3)

Intially, *D*(0) encompasses the mid- and far-field regions. The near-field is then divided into two equal parts: the lower half is assigned to *D*(1), while the upper half is further divided into two halves. The lower portion become *D*(2), and this process of dividing the upper half continues iteratively until the final partition, *D*(*s*), is reached. This reducing each partition by half is inspired by the HDR-LDR partitioning performed in the log-base-2 domain, which will be detailed in the next subsection. Note that the layer index *i*, which corresponds to the growth rate of the function *p*, decreases as depth increases. At greater depths, the impact of adjusting the polynomial growth rate becomes more noticeable. Therefore, reducing the frequency of changes ensures that the overall intensity changes remain less abrupt. This approach also helps control the growth rate and prevents it from rising too quickly, as observed in polynomial or exponential cases.

**Fig 4:**
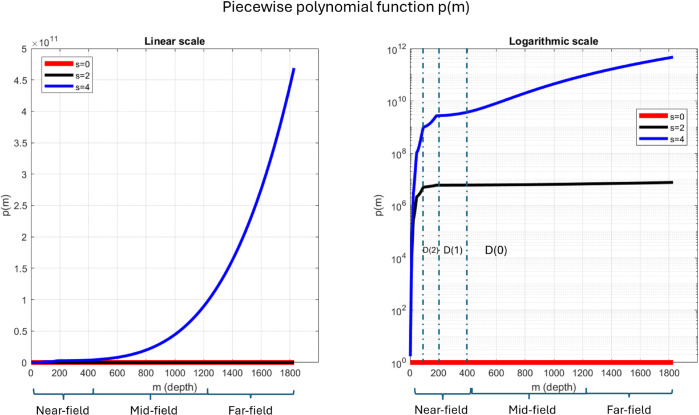
Piecewise polynomial function *p*(*m*) for *s* = 0 (red), *s* = 2 (black), *s* = 4 (blue), shown in linear scale (left) and logarithmic scale (right), with near-field, mid-field, and far-field regions marked. In the linear scale, the graph of *p*(*m*) for *s* = 4 follows an exponential-like form, while *s* = 0 and *s* = 2 have much lower values, appearing nearly overlapping. In the logarithmic view, the increasing steepness across partitions D(0),D(1),D(2),· reflects the rising polynomial order. *D*(0) exhibits the flattest slope, followed by progressively steeper curves for *D*(1),*D*(2), and subsequent partitions. Small kinks at the partition boundaries indicate changes in polynomial order.

The piecewise polynomial *p*(*m*) and its changes in growth rate is displayed in Fig 4

### Step 2: Converting HDR data into set of LDR images

To enhance contrast and improve visibility of specific areas of interest, which is especially critical in ultrasound imaging where certain tissues or abnormalities need better visibility, we separate details from different ultrasound intensity ranges into distinct images. Our partitioning method treats different intensity intervals as distinct information sources, enabling the algorithm to extract features optimally matched to the signal characteristics within each interval.

In our model, the depth-compensated HDR data is partitioned based on its cumulative histogram in the logarithmic domain. For each bin, pixels outside the bin are transformed into white, while the remaining pixels are linearly scaled to produce LDR images with the same dynamic range. Using a logarithmic scale is a natural and effective choice, as it better aligns with the physical signal behavior, given that ultrasound intensities attenuate exponentially with depth. Moreover, the cumulative-histogram-based partitioning, inspired by histogram equalization, effectively redistributes the highly skewed and uneven intensity levels in HDR-US caused by tissue and depth variations.

**Fig 5:**
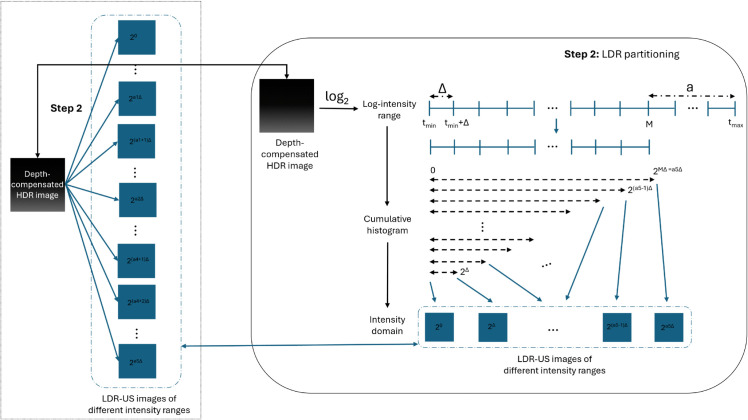
Overview of the HDR-to-LDR image partitioning process. Step 1: the intensity range is transferred to the logarithmic domain. Step 2: eliminate extremely high intensity values to reduce the maximal range we wish to represent. Step 3: the cumulative histogram with bins of size Δ is then calculated. Step 4: for each bin, an LDR image is constructed by transforming pixels that do not belong to the bin into white and scaling the remaining pixels linearly to fit the LDR.

Segmenting using histogram bins enables independent adjustment of different intensity regions, facilitating focused processing while reducing noise. By separating low-, mid-, and high-intensity components, each can contribute structural cues at appropriate resolutions, improving the visibility of subtle tissue differences, low-contrast regions, and overall image uniformity. The workflow is illustrated in Fig 5, with an example series shown in Fig 12.

Precisely, from the HDR data $\hat{I}$, we calculate the series of LDR images $\{I_{k}:\Omega \to [0,1]{\}}_{k}$ as follow. Let

$$I_{min}:=\min_{\begin{array}{c} (m,n)\in \Omega \\ \hat{I}(m,n)\neq 0 \end{array}}\hat{I}(m,n),\;I_{max}:=\max_{(m,n)\in \Omega }\hat{I}(m,n)$$ (4)

be the (non-zero) minimal and maximal intensity value of $\hat{I}$, respectively. The minimum $I_{\min}$ must be non-zero to allow logarithmic calculations. To avoid issues with zero intensities, a small constant ϵ>0 can be added to *I*_*min*_, chosen relative to the minimum meaningful signal. A very small *ϵ* forces the logarithmic domain to extend to extremely low values, generating many LDR images with nearly zero intensities. These images usually contain little or no useful information.

We begin by taking the maximal intensity interval in the logarithmic domain:

$$[t_{\min},t_{\max}]:=\left[-\lceil \log_{2}(I_{\max})\rceil ,-\lfloor \log_{2}(I_{\min})\rfloor \right]⊇\left[-\log_{2}(I_{\max}),-\log_{2}(I_{\min})\right].$$ (5)

This interval is then discretized into equidistant points with a chosen step size Δ>0:

$$\left\{t_{min}+k\Delta \;|\;k=0,1,...,\frac{t_{max}-t_{min}}{\Delta }=:K\right\}.$$ (6)

Finally, we construct a series of LDR images $\{I_{k}{\}}_{k=0,\cdots ,K}$, whose gray-scaled pixels belong to the respective bins in the intensity domain ${\left\{B_{k}=[0,\frac{I_{max}}{2^{k\Delta }})\right\}}_{k=0,\cdots ,K}$, and details within the intensity range $b_{k}=[\frac{I_{max}}{2^{k\Delta }},I_{max}]$ are displayed as white pixels in each image *I*_*k*_. That is, for every k=0,⋯,K,

$$I_{k}(m,n)\left\{ \begin{array}{cc} \in [0,1)\; & \text{if }\hat{I}(m,n)\in B_{k}\\ =1\; & \text{otherwise} \end{array}\right..$$ (7)

In particular, by scaling the depth-compensated HDR-US image $\hat{I}$, as defined in Eq 1, by each $2^{t_{min}+k\Delta }$ in Eq 6, all pixels within the bin *B*_*k*_ are mapped to the range [0,1). Indeed, if $(m,n)\in B_{k}$, or $\hat{I}(m,n)<\frac{I_{max}}{2^{k\Delta }}=2^{-t_{min}-k\Delta }$, then


$$2^{t_{min}+k\Delta }\hat{I}(m,n)<2^{t_{min}+k\Delta }2^{-t_{min}-k\Delta }=2^{0}=1.$$

After scaling, the intensity of pixels outside *B*_*k*_ exceeds one and is therefore set to one, ensuring that the intensity range of the resulting LDR image matches the desired range specified in Eq 7. Formally, each LDR image *I*_*k*_ is determined by

$$I_{k}(m,n):=\left\{ \begin{array}{cc} 2^{t_{min}+k\Delta }\hat{I}(m,n)\; & if\;2^{t_{min}+k\Delta }\hat{I}(m,n)\in [0,1]\\ 1\; & if\;2^{t_{min}+k\Delta }\hat{I}(m,n)>1 \end{array}\right..$$ (8)

White pixels are treated as regular intensity values, with the literal value 1 included in the image and contributing to subsequent calculations. With this construction, we ensure that each pixel is compressed at a logarithmic level adjusted according to the dynamic range it belongs to, allowing details in different dynamic ranges to be visible in the respective LDR image. This effect can be observed in Fig 12. For *k* = 0, the bin *B*_0_ consists of all the pixels in the image, i.e., $B_{0}=[0,I_{max}]$. Thus, the entire image is compressed using the same large logarithmic level, causing the mid-range intensity to become very small after compression, resulting in a completely black image with no visible detail. As *k* increases, the bins *B*_*k*_ contain fewer pixels, its inverse bin *b*_*k*_ becomes larger, and more pixels appear as white, revealing additional details.

Bins *B*_*k*_ that contain only a small fraction of the total pixels, typically 5−10%, are discarded, as they are largely uninformative. In practice, such bins typically correspond to extreme intensity values that are often noise and contribute little useful information. Specifically, we discard images *I*_*k*_ in which less than a chosen fraction p∈[0.05,0.1] of pixels have intensity below 1, ensuring that only intensity ranges representing the majority of the tissue signal are retained. Formally, images *I*_*k*_ satisfying

$$\frac{\#\{(m,n):I_{k}(m,n)<1\}}{MN}<p\text{ for a chosen }p\in [0.05,0.1],$$ (9)

where *M*,*N* are the discretizations of depth and radial components, are excluded. This prevents the final images, constructed from all *I*_*k*_, from being overexposed, as retaining bins with many extreme white pixels (intensity exactly 1) would make the overall intensity unbalanced.

Discarding sparse bins is implemented by reducing the maximum dynamic range represented, effectively eliminating unwanted *k* values and narrowing the interval to

$$[t_{\min},t_{\max}-a],\;\text{with }0<a<t_{\max}-t_{\min},a\in \mathbb{N}.$$ (10)

The parameter *a* is determined according to the percentile rule above:

$$a:=t_{max}-\min\left\{k:\frac{\#\{(m,n):I_{k}(m,n)<1\}}{MN}<p\right\}.$$ (11)

In practice, this involves first choosing *p*, then identifying the smallest index *k* that violates the threshold in Eq 9, and finally computing *a* from the resulting *k*. For datasets acquired under consistent settings, this procedure only needs to be performed once on a representative image. The obtained *a* can then be fine-tuned heuristically within a small range (±2−4) to optimize for specific cases (see Section Tunable parameters).

The parameter *a* controls dynamic range compression: Higher value of *a* enhances the contrast of the compressed image. A larger maximum dynamic range *I*_*max*_ typically requires a larger value of *a*. If *a* is too small, the image appears overexposed; if *a* is too large, the image becomes underexposed. By applying this threshold, we reduce the influence of sparsely populated bins, which are often dominated by noise, resulting in a more meaningful and visually balanced representation.

### Step 3: Pyramidal decomposition

In this step, a multi-scale representation, the Laplacian pyramid, is constructed for each image in the set of LDR images as outlined in [34]. The Laplacian pyramid, widely recognized for its effectiveness, isolates and preserves details across various spatial scales. By decomposing each LDR image into multiple levels, we capture both coarse and fine details across different intensity ranges, allowing them to be analyzed and manipulated independently. This coarse-to-fine approach enhances specific details while maintaining the overall structure, improving ultrasound image visualization by preserving fine details across varying scales, particularly in regions with complex or subtle anatomical features.

**Fig 6:**
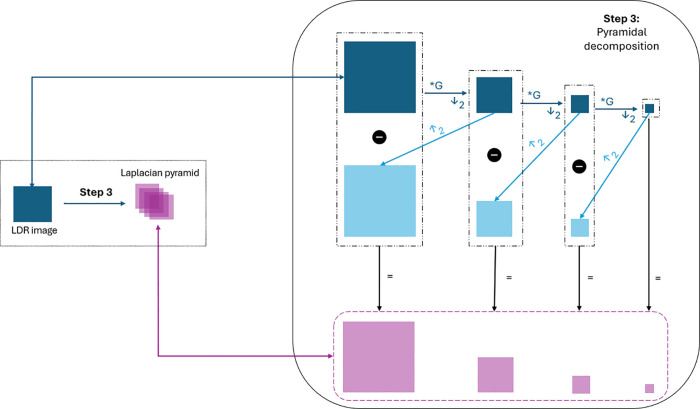
Overview of pyramidal decomposition process. Step 1: Gaussian pyramid progressively smoothing using Gaussian filter and subsampling by a factor of 2 in both coordinate directions (dark blue figures). Step 2: image interpolation is performed between two adjacent levels of resolution (light blue figures). Step 3: Laplacian pyramids (pink figures) is calculated as pixel-by-pixel differences between two consecutive levels of the Gaussian pyramid. The smallest level is not a difference image.

To calculate a Laplacian pyramid, the image is first processed to create a sequence of progressively lower-resolution versions, each representing coarser structures. At each level, the image is smoothed using a Gaussian filter, and the result is subtracted from the original resolution at that level to extract the high-frequency details, which represent the fine details of the image such as edges, textures, and small structures. These differences, or residuals, form the Laplacian levels. The process is repeated until the desired number of levels is reached, with the smallest level storing the coarsest approximation of the image and each subsequent level representing finer and more detailed features. The procedure is summarized in Fig 6.

We begin by iteratively smoothing the images with a Gaussian filter to remove high-frequency components, creating a pyramid of Gaussian-filtered images. After smoothing with a Gaussian filter, redundancy arises because neighboring pixels often have similar or nearly identical values, especially in smooth regions or areas with gradual intensity changes. Removing these redundant pixels has little visual impact—since their values are nearly identical—but significantly reduces the computational load for tasks performed at coarser scales. Moreover, without subsampling, the transition between levels becomes unclear, as high-frequency details from the higher-resolution image may remain and mix with those from the next level, causing a lack of clear separation between scales. As a result, the subtraction process used to calculate the Laplacian pyramid could cause high-frequency details from higher-resolution images to blend with those from lower-resolution ones, making it harder to distinguish between large structures and small details. Therefore, we subsample the smoothed results by a factor of 2 in both coordinate directions at each level, selecting every second pixel to reduce the resolution.

The resulting hierarchical structure, called the Gaussian pyramid, captures coarser structures at lower levels and finer details at higher levels, with each pixel representing the local average of neighboring pixels from the previous level. This procedure, which details the steps of Gaussian smoothing and subsampling, is illustrated in Step 1 of Fig 6.

In a formal setting, consider the set of LDR images $\{I_{k}{\}}_{k=0,\cdots ,K}$ defined in Eq 8. For each k=0,1,...,K, let $I_{k}^{0}:=I_{k}$. Recursively, for d∈ℕ, we calculate

$$I_{k}^{d}(m,n):=\sum_{i=-2}^{2}\sum_{j=-2}^{2}G(i,j)I_{k}^{d-1}(2m+i,2n+j),$$ (12)

where *G* is a Gaussian kernel. By definition, the dimension of $I_{k}^{d}$ is half the dimension of $I_{k}^{d-1}$ in both coordinate directions. Since downsampling to a factor of 2 reduce the dimension in each direction by half, the maximal number of pyramid levels is

$$l:=\lfloor \log_{2}(\min\{M,N\})\rfloor ,$$ (13)

where *M*,*N* is the height and width of the original image, respectively. That is, we have d=0,1,⋯,l. For every image $I_{k},k=1,\cdots ,K$, the sequence of images ${\left\{I_{k}^{d}\right\}}_{d=0,\cdots ,l}$ is called the Gaussian pyramid of *I*_*k*_.

We now compute the Laplacian pyramid by taking the differences between two consecutive levels of the Gaussian pyramid. The smallest level, however, is not a difference image but serves as the base for reconstructing the high-resolution image. To calculate pixel-by-pixel differences, image interpolation is performed between adjacent resolution levels, as illustrated in Step 2 of Fig 6. The subtraction process is shown in Step 3 of Fig 6.

Formally, the Laplacian pyramid is defined by

$$L_{k}^{d}:=\left\{ \begin{array}{cc} I_{k}^{d}\; & for\;d=l\\ I_{k}^{d}-\hat{I_{k}^{d+1}} & otherwise \end{array}\right..$$ (14)

Here, since the resolution in each coordinate of the image at (d+1)−th level, $I_{k}^{d+1}$, is half that of the previous level, $I_{k}^{d}$, it must first be upsampled to match the resolution of $I_{k}^{d}$ before calculating the difference. That is, for each level d=1,⋯,l, we calculate the upsampling

$$\hat{I_{k}^{d}}(m,n):=4\sum_{i=-2}^{2}\sum_{j=-2}^{2}G(i,j)I_{k}^{d}\left(\frac{2m+i}{2},\frac{2n+j}{2}\right)$$ (15)

of $I_{k}^{d}$ in both coordinate directions by a factor of 2, so that $\hat{I_{k}^{d}}$ matches the resolution of $I_{k}^{d}$. This process involves interpolating new pixels between the original pixels of $I_{k}^{d}$ using the weights defined by the Gaussian filter *G*, which are normalized to preserve intensity scaling. At image borders, replication is used, i.e., the nearest border pixel is repeated to provide input for the convolution.

### Step 4: Weighted multi-resolution fusion

In this step, we introduce a new scheme for determining the weights used in weighted fusion, derived from the spatial depth information of the ultrasound data. This approach highlights the critical role of depth in mitigating signal attenuation and improving the visibility of deeper structures. In far-field regions, signal attenuation makes it particularly challenging to distinguish anatomical structures from noise. Building on the separation of noise and critical details achieved through the multi-resolution approach, this step implements a depth-adaptive weighting scheme. By assigning lower weights to noise-dominated regions and higher weights to areas with critical details, this method enhances the visibility of meaningful structures while effectively suppressing noise.

To enable depth-specific processing, the ultrasound images are divided into five depth-based regions: near-field, transitioning near-to-mid field, mid-field, transitioning mid-to-far field, and far-field, based on the acquisition setting. This segmentation accounts for the inherent variations in anatomical structures, signal characteristics, and noise levels across different depths, enabling a targeted focus on the unique challenges and features of each region.

Previously, we used histogram partitioning to divide the ultrasound image based on intensity values. By incorporating depth information into partitioning, we can better focus on improving contrast and reducing noise, providing more precise control over the process. Our approach combines the strengths of both histogram-based and depth-based partitioning, leveraging the simplicity of intensity-based methods alongside the precision of depth-specific techniques. The partitioned image sets and their corresponding weights are then calibrated to achieve the best alignment with our fetal ultrasound datasets (see Section Tunable parameters for details).

**Fig 7:**
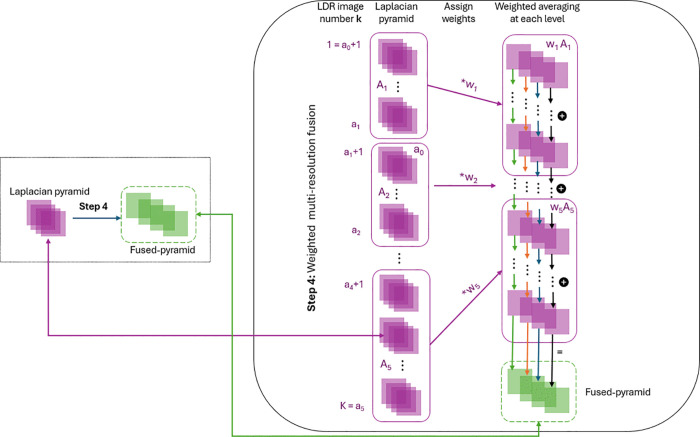
Overview of the weighted fusion process. Step 1: the Laplacian pyramids of all LDR images $\{L_{k}^{d}{\}}_{k=1,\cdots ,K}$ are partitioned into 5 sets $A_{1},A_{2},\cdots ,A_{5}$. Step 2: weights are assigned to the corresponding sets. Step 3: at each level d=1,⋯,l, weighted averaging ais performed on the *d*-th level of the Laplacian pyramid $\{L_{k}^{d}{\}}_{k=1,\cdots ,K}$ to obtain the *d*-th level of the final fused Laplacian pyramid *L*^*d*^.

Following this, we fuse the series of Laplacian pyramids using a weighted multi-resolution approach, as described in [30]. A weight, heuristically determined, is assigned to each pyramid in the series, and at each pyramid level, the weighted averages are computed. The resulting fused pyramid is then combined into the final tone-mapped image, as detailed in the next section. The weighted-averaging process is illustrated in Fig 7.

Rigorously, the LDR image series $\{I_{k}{\}}_{k=0,\cdots ,K}$ from Eq 8 is divided into 5 distinct sets, each corresponding to a different depth-specific region:

$${\left\{A_{i}:=\{I_{k}\;|\;k=a_{i-1}+1,\cdots ,a_{i}\}\right\}}_{i=1,\cdots ,5},$$ (16)

with their corresponding weights $\{w_{i}{\}}_{i=1,\cdots ,5}$. Here, each *a*_*i*_ for i=1,⋯,5 denotes the index of the last image in its corresponding set *A*_*i*_. The first image of the set *A*_1_, $I_{a_{0}+1}$, is *I*_0_, and the last image of the set *A*_5_ is *I*_*K*_, where *K* is the total number of LDR images, determined in Step 2, Eq 6.

The fusion process is then performed at each pyramid level d∈{0,...,l}, as described in [30]. At each level, the *d*-th layer of each Laplacian pyramid $L_{d}^{k}$, computed using Eq 14, is assigned a weight according to its corresponding set $A_{1},\cdots ,A_{5}$. The sets and their weights are determined in Eqs 16 and 19. A weighted averaging is then applied to obtain the *d*-th level of the final fused Laplacian pyramid, given by:

$$L^{d}(m,n)=\;{(\sum_{j=1}^{5}w_{j}|A_{j}|)}^{-1}\left(\sum_{j=1}^{5}w_{j}\sum_{k=a_{j}-1}^{a_{j}}L_{k}^{d}(m,n)\right),$$ (17)

with (m,n)∈Ω, for all d=1,⋯,l. Here, $L_{k}^{d}$ denotes the *d*–th level of the Laplacian pyramid of the LDR image *I*_*k*_,|*A*_*j*_| represent the number of elements, *a*_*j*_ and *a*_*j*−1_ are the upper and lower bounds, respectively, and *w*_*j*_ is the weight of the set *A*_*j*_ for each j=1,⋯,5.

### Step 5: Recombining the pyramid into an image

**Fig 8:**
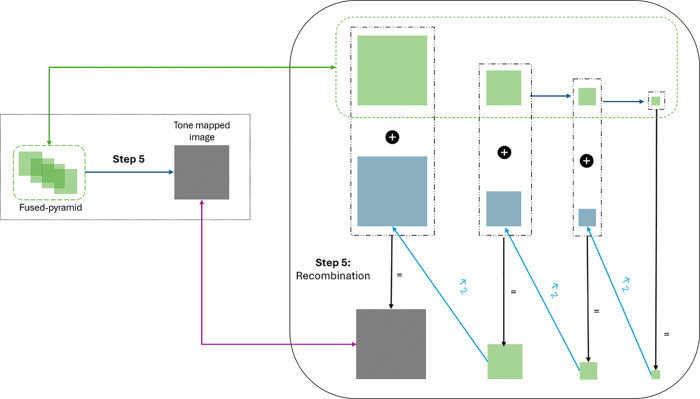
Overview of the pyramid recombination process, starting from the smallest scale (far right) and moving backward. Step 1: the smallest level of the Laplacian pyramid (green figures) serves as a base for reconstructing the high-resolution image. Step 2: image interpolation is applied to each layer of the reconstructed Gaussian pyramid (blue figures). Step 3: each level of the reconstructed Gaussian pyramid (gray figures) is calculated as the sum of the corresponding Laplacian level and the interpolated previous level of the Gaussian pyramid. The largest level of the Gaussian pyramid is the final tone-mapped image.

In the last step, the fused pyramid is recombined into the final tone-mapped image by inverting the pyramid generation procedure in Eq 14. We invert the pyramid decomposition exactly as performed in the forward step to ensure no loss of details during reconstruction. During this backward reconstruction, multi-scale information is smoothly merged back into a single image, minimizing artifacts that may otherwise appear in the tone-mapped result. In particular, fine details and edges from higher-frequency layers are progressively combined with lower-frequency components that capture the broader structural information, enabling effective blending of contrast and brightness adjustments across spatial scales while preserving image sharpness and clarity. This procedure is visualized in Fig 8.

Formally, from the fused Laplacian pyramid ${\left\{L^{d}\right\}}_{d=0,\cdots ,l}$, calculated in Eq 17, a Gaussian pyramid ${\left\{{\bar{I}}^{d}\right\}}_{d=0,\cdots ,l}$ of the resulting tone-mapped image *T*(*I*) is computed from the highest level *d* = *l* backwards. Each level d=0,1,⋯,l of the Gaussian pyramid is calculated by:

$${\bar{I}}^{d}=\left\{ \begin{array}{cc} L^{d}\; & if\;d=l\\ L^{d}+\hat{{\bar{I}}^{d+1}} & otherwise, \end{array}\right.$$ (18)

where $\hat{{\bar{I}}^{d+1}}$ is the upsampling of the Gaussian pyramid level ${\bar{I}}^{d+1}$ by a factor of 2 in both coordinate directions, calculated by Eq 15. The 0-th level of the Gaussian pyramid is the desired tone-mapped image, i.e., $T(I):={\bar{I}}^{0}$.

### Tunable parameters

All tunable parameters of our algorithm, which are determined heuristically, are summarized in Table 2. A sensitivity analysis of all these parameters is presented in the Results section.

**Table 2:** List of the heuristically computed tunable parameters used in the present study. The notations {·,·} and (·,·) denote discrete and continuous ranges, respectively.

	Tunable parameters	Range	Defaults	Description
**Step 1:**	*s*	{0,1,⋯,10}	4	Number of partitions.
**Step 2:**	Δ	[0,2]	1	Discretization step size.
	*a*	{0,2,⋯,30}	26	Cutting parameter.
**Step 3:**	n×n	{3,5,7}	5	Size of Gaussian kernel.
**Step 4:**	$a_{1},w_{1}$	$\left\{\lfloor \frac{K}{4}\rfloor -1,\lfloor \frac{K}{4}\rfloor +1\},(2,3\right)$	$\lfloor \frac{K}{4}\rfloor ,3$	Upper bound and weight of set *A*_1_.
	$a_{2},w_{2}$	$\left\{\lfloor \frac{K}{2}\rfloor -1,\lfloor \frac{K}{2}\rfloor +1\},(2,3\right)$	$\lfloor \frac{K}{2}\rfloor ,2.5$	Upper bound and weight of set *A*_2_.
	$a_{3},w_{3}$	$\left\{\lfloor \frac{3K}{4}\rfloor -2,\lfloor \frac{3K}{4}\rfloor \},(2,3\right)$	$\lfloor \frac{3K}{4}\rfloor -1,3$	Upper bound and weight of set *A*_3_.
	$a_{4},w_{4}$	$\left\{\lfloor \frac{3K}{4}\rfloor ,\lfloor \frac{3K}{4}\rfloor +2\},(1,2\right)$	$\lfloor \frac{3K}{4}\rfloor +1,1.5$	Upper bound and weight of set *A*_4_.
	$a_{5},w_{5}$	{K},(1,2)	*K*,1	Upper bound and weight of set *A*_5_.
			where *K* is the number of LDR images acquired from Step 2	

**Fig 9:**
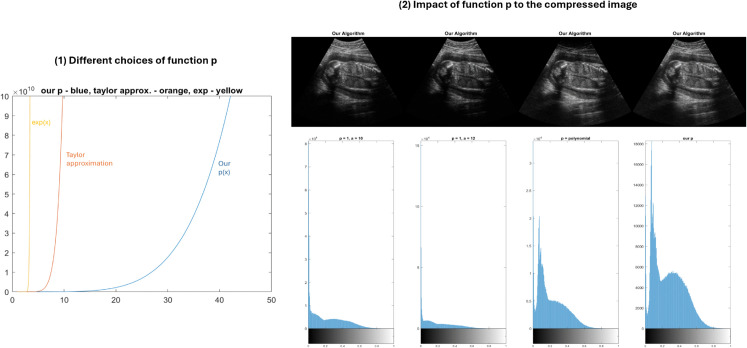
(1) The piecewise polynomial function *p* with *s* = 4 (blue), the 8th-order Taylor polynomial (orange), and the exponential functions (yellow) are compared. Our function behaves like the exponential function in lower-depth regions but grows more slowly with increasing depth. **(2)** Results and histograms: **1st and 2nd images**: Without a depth-increasing function (p(x)=1), contrast distribution depends on *a*. At *a* = 10, the far-field has good contrast (gCNR = 0.67), but the mid-field shows low contrast with noise (gCNR = 0.37 and 0.17), with entropy of 6.80. Increasing *a* to 12 improves mid-field contrast (gCNR = 0.40 and 0.21) but underexposes the far-field (gCNR = 0.60), lowering entropy to 6.42. Both histograms are right-skewed, with higher kurtosis at *a* = 12, indicating greater underexposure. **3rd image**: using a 4th-order polynomial function 4 (*p*(*x*) = *x*^4^). This improves far-field detail visibility (gCNR = 0.70 and reduces mid-field noise (gCNR = 0.54 and 0.21), but results in a loss of near-field detail (entropy = 6.30). The histogram is close to a bell shape but remains right-skewed, with low kurtosis due to underexposed near-field. **4th image**: using a piecewise polynomial function with *s* = 4. This approach achieves balanced contrast across all depths without significant detail loss (gCNR = 0.72 in the far-field; 0.68 and 0.22 in the mid-field; entropy = 7.27). The histogram shows a more balanced distribution.

**Step 1:** In the depth compensation step, the piecewise polynomial depth function *p*(*m*) in Eq 1 is chosen with *s* = 4. The impact of the parameter *s* and the choice of the function *p* is illustrated in Fig 9. In the first and second images, we observe that without multiplication by an increasing depth function, either the mid-field remains low in contrast or the far-field becomes underexposed. The next two images demonstrate the advantage of using a piecewise polynomial function over a standard polynomial: employing a piecewise polynomial preserves details in the near-field, whereas using a high-degree polynomial alone results in the loss of near-field details after compression. Furthermore, the histogram corresponding to the piecewise polynomial result is more equalized compared to those of the other results, indicating a more balanced contrast distribution across depth regions. A detailed sensitivity analysis of the parameter *s* is presented in the Results section (see Table 5).

**Step 2:** In the step of converting HDR data to a set of LDR images, the cutting parameter is set to *a* = 26 (Eq 10), and the discretization step size is set to Δ=1 (Eq 6).

**Fig 10:**
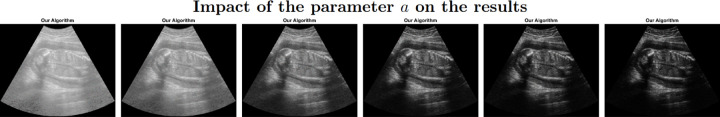
Results of our algorithm with varying *a* values (left to right: *a* = 0, 10, 20, 26, 28, 30). A higher *a* yields stronger contrast. For a=0,10,20, the images appear overexposed, with symmetric histograms and high kurtosis. Entropy scores rises with *a* (6.57,6.89,7.28). The contrast improves gradually: far-field gCNRs are 0.44, 0.53, and 0.55; kidney-fluid: 0.31, 0.41, 0.52; kidney-spine: 0.19, 0.22, 0.23. At *a* = 30, the image becomes underexposed with excessive contrast, reflected by a right-skewed histogram and a low entropy of 6.81; the contrast is high, with gCNRs of 0.70 (far-field), 0.61 (kidney-fluid), and 0.21 (kidney-spine). The best overall performance is achieved at *a* = 26 and *a* = 28, both producing symmetric histograms with low kurtosis, indicating balanced contrast. The setting *a* = 26 performs better, with entropy of 7.27 vs. 7.17; far-field gCNR of 0.72 vs. 0.68; kidney-fluid gCNR of 0.67 vs. 0.64; and kidney-spine gCNR of 0.21 vs. 0.19.

Choosing this value of *a* is equivalent to discarding the cumulative histogram bins that contains fewer than 10% of the total numbers of pixels (*p* = 0.1), as these primarily represent noise and lack meaningful information. Fig 10 illustrates the effect of the parameter *a*, while Fig 12 shows the visual results of the second step. We observe that for the images where k=a/2≥13, no valuable information is present, and thus these images are discarded. Furthermore, after applying depth compensation, the number of such white images increases significantly, further emphasizing the need for effective processing to handle these cases. A detailed sensitivity analysis of the parameter *a* is presented in the Results section (see Table 6).

For the logarithmic calculation, instead of adding a small *ϵ* to zero intensities, we define *I*_*min*_ as the smallest non-zero intensity in the data (for our dataset, this is approximately equivalent to adding $\epsilon =10^{-3}$). Using a smaller *ϵ* would increase *t*_*max*_ and, consequently, the number of bin to be discarded *a*, which is unnecessary since the chosen value *a* = 26 already filters these bins.

**Fig 11:**
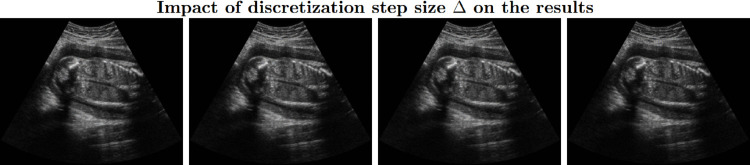
Results of our algorithm with varying Δ values (left to right: Δ=0.1,0.5,1,2). Lower Δ produces brighter images. The histograms for all settings are generally symmetric with low skewness, but smaller Δ values yield distributions that are more centered and smoother, resembling a Gaussian shape. Entropy is highest at Δ=1 (7.15,7.10,7.27,6.94), and EPI remains consistent across setting (≈0.22), indicating that Δ=1 preserves the most information. Mid-field and far-field contrast decrease with increasing Δ (0.72,0.69,0.68,0.60 and 0.73,0.72,0.72,0.69), whereas local hyperechoic-hypoechoic contrast in the mid-field increases (0.17,0.18,0.22,0.29). Computational time increases substantially for lower Δ values (4.89,1.37,0.7,0.42s). Overall, Δ=1 provides the best balance between information preservation, contrast, tissue differentiation, natural histogram shape, and computational efficiency.

Fig 11 illustrates the effect of the parameter Δ. Choosing Δ=1 achieves an optimal trade-off among information preservation, contrast, tissue differentiation, natural histogram characteristics, and computational efficiency. A detailed sensitivity analysis of the parameter *a* is presented in the Results section (see Table 7).

**Fig 12:**
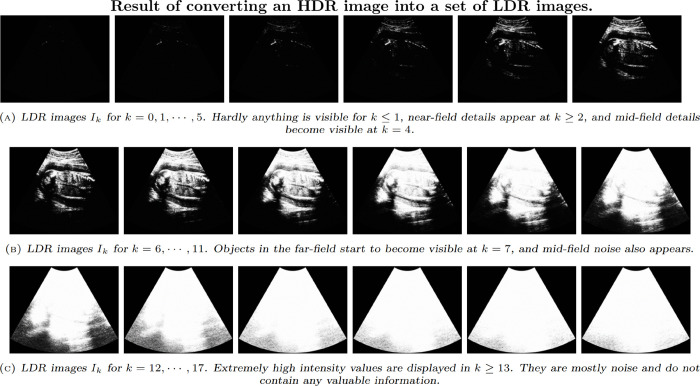
Series of LDR images generated from the original HDR-US data with a=0,Δ=2.

**Fig 13:**
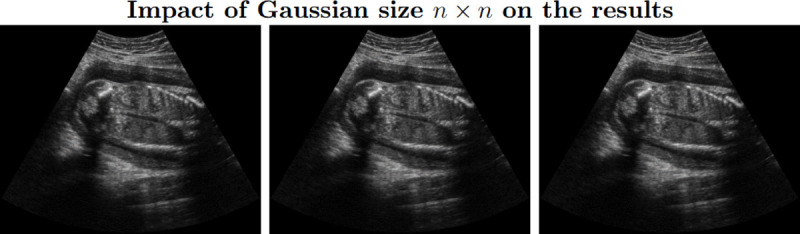
Results of our algorithm with varying size of the Gaussian kernel n×n values (left to right: n=3,5,7). Varying the Gaussian kernel size produces no meaningful visual or quantitative differences in the output. Across all kernel sizes, the images retain similar brightness, contrast, and structural details (similar entropy, EPI, gCNR values and histogram). Computation times are also comparable, with the 5×5 kernel yielding the lowest and most stable performance (average time with standard deviation 0.54±0.11,0.53±0.07,0.64±0.09).

**Step 3:** The pyramidal composition (Eqs 12 and 15) is calculated using a 5×5 Gaussian kernel, commonly used in image processing for effective noise reduction while preserving important details, balancing performance and computational efficiency, and providing a reliable approximation of natural blurring. Varying the Gaussian kernel size n×n produces no meaningful visual or quantitative differences in the output, and the 5×5 kernel yields the lowest and most stable computational time. The effect of different kernel sizes is illustrated in Fig 13, and a detailed sensitivity analysis is provided in the Results section (see Table 8).

**Step 4:** The weights for the weighted-fusion process are

$$\left\{ \begin{array}{c} a_{0}:=-1,a_{1}:=\lfloor \frac{K}{4}\rfloor -1,\;a_{2}:=\lfloor \frac{K}{2}\rfloor ,a_{3}:=\lfloor \frac{3K}{4}\rfloor -1,a_{4}:=\lfloor \frac{3K}{4}\rfloor +1,a_{5}:=K\\ \text{and}\\ w_{1}=3,w_{2}=2.5,w_{3}=3,w_{4}=1.5,w_{5}=1. \end{array}\right.$$ (19)

The reason is that the histogram distribution of the near-field and far-field regions varies significantly. In the near-field, the energy is strong, whereas the far-field signal is weaker and contains more noise. Consequently, the first set, *A*_1_, primarily consists of histogram bins with extremely low values, while *A*_5_ contains extremely high values. To balance the histogram distribution, a higher weight is assigned to *A*_1_, and a lower weight to *A*_5_. Increasing *w*_5_ or decreasing *w*_3_ would result in an overexposed image.

**Fig 14:**
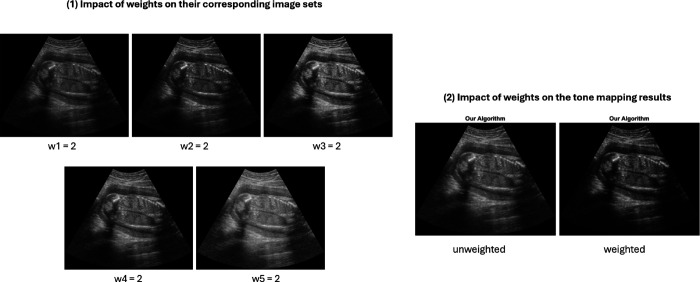
(1) Results of multi-resolution fusion by varying one weight at a time (others fixed at 1) to isolate each weight’s effect. Increasing $w_{1}$ darkens the image due to greater emphasis on black pixels and near-field details. Both histograms remain symmetric with low kurtosis, but the one for *w*_1_ = 2 is more left-shifted. Entropy decreases from 7.38 to 7.12, and gCNR values drop from 0.59 to 0.53 (far-field), 0.61 to 0.60 (kidney-fluid), and 0.17 to 0.16 (kidney-spine). Raising $w_{2}$ or $w_{3}$ improves mid-field contrast (kidney-spine gCNR = 0.22), with *w*_3_ having a stronger effect on kidney-fluid (gCNR: 0.68 for *w*_3_ = 2, 0.55 for *w*_2_ = 2). However, setting *w*_2_ or *w*_3_ higher than *w*_1_ leads to overexposure due to reduced dark pixel contribution. Histograms are right-shifted with low kurtosis. Entropy is 7.20 for *w*_2_ = 2 and 7.50 for *w*_3_ = 2; far-field gCNR is 0.45 and 0.65, respectively. Weights $w_{4}$ and $w_{5}$ affect far-field contrast, with *w*_5_ more sensitive to noise in amniotic fluid. Setting either higher than the others leads to overexposure in near- and mid-field regions. Histograms remain symmetric with high kurtosis. The far-field gCNR is 0.63 and 0.52; kidney-fluid is 0.66 and 0.63; kidney-spine is 0.22 and 0.17. Entropy remains at 7.4 in both cases. **(2)** Multi-resolution fusion results without weighting (left) and with optimized weights (right). The optimized result shows improved contrast and reduced noise, especially in the amniotic fluid regions. Both histograms are bell-shaped but the weighted version has lower kurtosis. With optimized weights, the gCNR scores improve from 0.59 to 0.72 (far-field), 0.61 to 0.67 (kidney-fluid), and 0.16 to 0.22 (kidney-spine), while entropy drops from 7.38 to 7.27.

The transitioning regions between the near- and mid-field are grouped into *A*_2_, while the mid-field region is represented by *A*_3_. Since the object of interest predominantly lies in these regions, higher weights—*w*_2_ and *w*_3_—are assigned, with *w*_3_ being greater than *w*_2_. This is because anechoic mid-field areas (e.g., amniotic fluid) often exhibit noise, which we aim to suppress. Similarly, *A*_4_ corresponds to the transitioning mid-to-far and far-field regions. While these regions are prone to noise like *A*_5_, they still contain meaningful information. As a result, the weights for *A*_4_ are kept lower than *w*_2_ and *w*_3_ to minimize noise amplification, but still higher than *w*_5_. The influence of the weights on their corresponding image sets is illustrated in Fig 14. A one-at-a-time sensitivity analysis of the weights *w*_*i*_ and the corresponding set boundaries *a*_*i*_ is presented in the Results section (see Table 9).

## Materials and experiments

### Study design

In this prospective observational study, our ultimate goal is to address the diagnostic challenge of visualizing and assessing fetal kidneys, particularly in the second trimester. This design enables standardized, real-time data collection during routine clinical practice without intervention, ensuring ethical suitability in a prenatal setting. Serving as a preliminary methodological investigation, the study establishes the technical feasibility and diagnostic potential of tone mapping in ultrasound imaging, providing a foundation for future clinical studies to evaluate its impact on prenatal diagnostic accuracy.

In this context, we tested the hypothesis that the proposed tone mapping algorithm, which incorporates depth compensation, multi-resolution processing, and depth-informed weighting, will improve the quality of fetal ultrasound images compared to existing tone mapping methods. This improvement was evaluated based on three key criteria: contrast resolution, preservation of anatomical details, and tissue differentiation, with a focus on clarifying fetal kidneys and structures located in deeper tissue layers.

To evaluate this, we compared conventional B-mode images with tone-mapped results obtained by applying traditional tone mapping algorithms listed in Table 1 as well as our method applied to the raw HDR-US data. Objective image quality assessment (IQA) were applied to 20 fetal kidney images, with details on the IQA metrics provided in a later subsection.

This work primarily serves as a technical feasibility study, laying the groundwork for a larger clinical investigation. The sample size was selected based on practical considerations and the availability of high-quality data. A more extensive clinical study with a larger sample size is planned for future work to provide a comprehensive evaluation and validation.

**Data acquisition.**

Sonographic transabdominal images of the fetal kidneys were obtained at the Medical University of Vienna, Department of Obstetrics and Gynaecology from April 13th, 2023 to January 29th, 2024. All images were acquired according to the ISUOG Practice Guidelines (updated): performance of the routine second-trimester fetal ultrasound scan [36]. In accordance with established clinical guidelines, the inclusion criteria for the study consisted of pregnant women between 20 and 24 weeks of gestation (19+0 to 23+6 weeks of gestation), aged 18 years or older, with a singleton pregnancy. During this gestational window, fetal kidney anatomy is optimally visualized. Exclusion criteria included multiple pregnancies, fetal malformations or genetic disorders, and fetal growth restriction.

Raw ultrasound data were acquired using the Voluson Expert 22 (GE Healthcare) ultrasound machine with a transabdominal curved transducer (C2-9), under the same acquisition settings: the routine obstetric preset (Routine/OB) with harmonic imaging (HI 7.97–4.15 MHz), default gain (Gn 0), a 7.0 MHz probe (C7.0), focus level 3 and edge enhancement level 2 (FF3/E2). The axial depth (distance to the transducer) and lateral width (lateral distance) of our data were 1828 and 464 pixels in the vertical and horizontal directions, respectively. All acquired images were stored locally on the ultrasound machine and in an electronic database for further analysis.

**Data security.**

All sampled images generated during this study are kept confidential in accordance with data safety policies of the Medical University of Vienna and University of Vienna. Therefore, images are stored pseudonymized and only authorized study personnel have access to the generated images. The study investigators and other study personnel do not use such data and records for any other purpose than conducting the study.

**Implementation.**

Traditional tone-mapping algorithms were implemented and their execution time was calculated in MATLAB R2024b, Intel i9-12900K CPU, NVIDIA RTX A4000 GPU, using the default parameters provided by [21,22,32,37,38]. Conventional B-mode images were directly acquired from the Voluson Expert 22 (GE Healthcare) ultrasound machine along with the raw HDR-US data. Quantitative metrics were then computed using MATLAB.

### Ethical approval

The study was approved by the local ethics committee of the Medical University of Vienna (2092/2022). The study was conducted in accordance with the current version of the Helsinki Declaration. All patients gave oral and written consent before study inclusion.

### Quantitative image analysis methods

The objective image quality assessment was completed using the Generalized Contrast-to-Noise Ratio (gCNR) [43] of 3 different regions of interest (ROIs), the image entropy [44] metrics and Edge Preservation Index (EPI). By analyzing the gCNR scores, we evaluated whether our method improves fetal tissues differentiation and enhances contrast between echoic and anechoic regions compared to existed tone-mapping algorithm. Comparison of image entropy highlights the method’s ability to preserve more of the original image’s information and detail during processing. The EPI scores evaluate how well edge information is maintained when tone-mapped HDR ultrasound images and complements image entropy, which can increase with noise and overestimate quality.

Furthermore, skewness [55] and kurtosis [56] of the image histogram were used as descriptive metrics of pixel intensity distribution, reflecting the asymmetry and sharpness of tonal values across the image, rather than as a direct measure of image quality. Together, these metrics provide complementary information on contrast, tonal distribution, and image characteristics.

#### Generalized contrast-to-noise ratio [43].

The gCNR score determines the separability rate between ROIs by evaluating how much their normalized histograms overlap. It is defined as

$$gCNR=1-\sum_{I}\min\{P_{1}(I),P_{2}(I)\},$$ (20)

where *I* represents the intensity value in the histogram, and *P*_1_(*I*) and *P*_2_(*I*) are probabilities of pixel intensity *I* in the first and second ROIs, respectively. The normalized histogram are calculated for intensity interval [0,1] using 256 bins.

An increase in gCNR indicates greater separability between the target regions. A high gCNR value (close to 1) indicates minimal overlap, meaning the two regions are well separated in terms of their intensity distributions. Conversely, a low gCNR value (closer to 0) suggests significant overlap between the histograms, indicating that the regions are not easily distinguishable.

In this study, three pairs of ROIs were compared:

1. Fetal kidney versus amniotic fluid.
2. Tissue versus fluid in the far-field.
3. Fetal kidney versus adjacent bone tissue.

**Fig 15:**
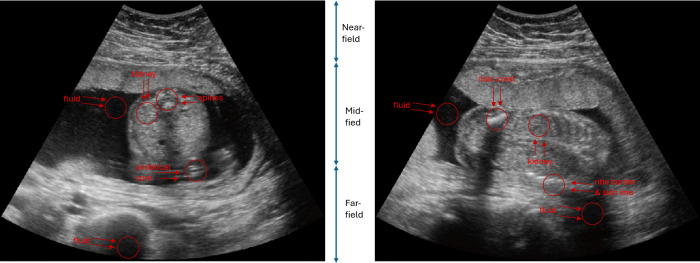
Example of ROIs in axial and coronal views (ROIs shown in red). Left: Axial view, showing fetal kidney and fluid in the mid-field for gCNR(i), umbilical cord and fluid in the far-field for gCNR(ii), and fetal kidney with spines for gCNR(iii). Right: Coronal view, showing fetal kidney and fluid in the mid-field for gCNR(i), rib borders with skin line and fluid in the far-field for gCNR(ii), and fetal kidney with iliac crest for gCNR(iii).

An example of the selected ROI pairs is shown in Fig 15. These ROIs were selected to evaluate the improvements in contrast resolution from different aspects. The first two pairs were used to assess contrast resolution, where an increase in gCNR indicates better contrast, as fluid should appear dark in ultrasound images. The third pair was used to evaluate tissue differentiation within the fetus, with an increase in gCNR reflecting improved differentiation due to the differing echogenicity of kidneys and calcified structures.

The ROI placement process was designed to be reproducible, with predefined criteria and reference guidelines ensuring consistent placement across different scans and raters. All ROIs were selected based on their anatomical locations within each scan. Due to variations in fetal position and scanning angles (axial and coronal), anatomical structures appeared in different regions of the image, and thus ROI locations varied across images. All anatomical structures were marked in accordance with the pictorial guidelines for second-trimester ultrasound outlined in [36,48]. The selection process was performed independently by multiple authors in consensus to ensure consistency and relevance across the dataset. To minimize potential bias, the raters were unaware of which processing algorithm had been applied to the images when selecting ROIs, ensuring that their choices were based solely on anatomical feature.

**Image entropy [44].**

Image entropy is often used to classify textures, serving as an indicator of the richness of information in an image. The larger the information entropy, the more detailed information contained in the image, indicating less loss of details or important contents during compression. In ultrasound imaging, a higher entropy value indicates greater variation in pixel intensities, suggesting more complex structures and better visibility of finer details. In contrast, low entropy might indicate blurred or noisy images with poor contrast, making it harder to distinguish between different tissues or abnormalities. The entropy is calculated based on the histogram of the image, which represents the probability distribution of pixel intensity values. For each tone-mapped image, we first computes the histogram and normalizes it by scaling the histogram counts so that the sums equals 1, converting counts to probabilities. Afterwards, we apply the entropy formula:

$$H=-\sum_{I}P(I)\log_{2}(P(I)),$$ (21)

where *P*(*I*) is the probability of the raw pixel value *I*.

**Edge preservation index [50,51].**

The Edge Preservation Index (EPI), also known as Edge Preservation Ratio (EPR), measures how well structural information, especially edges, is preserved between a raw HDR image and its tone-mapped version. In ultrasound, edges mark important anatomical boundaries that can be degraded by speckle noise or processing procedure. It is defined as


$$EPI=\frac{\sum Edge(I_{HDR})\cap Edge(I_{TM})}{\sum Edge(I_{HDR})},$$

where $Edge(I_{HDR})$ is the binary edge map of the HDR-US image and $Edge(I_{TM})$ is the binary edge map of the tone-mapped image. To calculate the edge map, the Canny edge detector [52], designed to identify true edges while minimizing noise, is used because it accurately localizes edges and avoids multiple responses for the same edge, and performs reliably in noisy images.

**Histogram skewness and kurtosis [54–56].**

Skewness and kurtosis are used to analyze the shape and distribution of image histograms, enabling better evaluation of image contrast, exposure and tonal distribution.

The skewness of an image histogram is a statistical measure often used to evaluate the well-exposedness of an image, which refers to the optimal balance of brightness and contrast that preserves details in both hypoechoic and hyperechoic regions. It characterizes the asymmetry in the distribution of pixel intensity values and is mathematically defined as:

$$\text{Skewness}=\frac{\frac{1}{MN}\sum_{m=1}^{M}\sum_{n=1}^{N}(I(m,n)-\mu {)}^{3}}{\sigma^{3}},$$ (22)

where *I*(*m*,*n*) represents the pixel intensity values at position (*m*,*n*) in the image, *μ* is the mean intensity, *σ* the standard deviation of the pixel intensities, *M* and *N* are the height and width of the image, respectively. A negative skewness value indicates a left-skewed histogram, where pixel intensities are concentrated toward lower values, often corresponding to underexposure and a loss of detail in hypoechoic regions. Conversely, a positive skewness value indicates a right-skewed histogram, where pixel intensities are concentrated toward higher values, suggesting overexposure and potential loss of detail in hyperechoic regions. A skewness value close to zero typically indicates a sym. histogram, but the interpretation depends on the overall distribution of pixel intensities.

Kurtosis, which quantifies the concentration of pixel intensities around the mean, provides additional insight into image quality. It is calculated as:

$$\text{Kurtosis}=\frac{\frac{1}{MN}\sum_{m=1}^{M}\sum_{n=1}^{N}(I(m,n)-\mu {)}^{4}}{\sigma^{4}}.$$ (23)

If the histogram is bell-shaped with high kurtosis (Kurtosis >3), where most pixel intensities are tightly clustered near the mean, the image may appear flat or low in contrast due to insufficient tonal variation. This occurs because high kurtosis indicates a sharp peak and heavy tails, concentrating intensities in a narrow range. Conversely, a histogram with low kurtosis (Kurtosis <3) is more evenly spread across the intensity range, with a flatter peak and lighter tails, suggesting a more balanced distribution of intensities. A bell-shaped histogram with Kurtosis  = 3 is a normal-distribution. Such a distribution enhances tonal variation and improves the richness of image details, which is crucial for accurately visualizing anatomical structures in ultrasound scans.

### Statistical analysis

A repeated-measures analysis of variance (RM-ANOVA) [49,53] was performed to assess the statistical significance of differences in EPI, entropy, and the three gCNR scores across 18 tone mapping algorithms, with each algorithm applied to the same 20 images. Effect sizes were reported as partial eta squared $\left(\eta_{p}^{2}\right)$, which indicates the proportion of variance explained by the factor of interest, with higher values reflecting a stronger effect. Exact F-statistics with degrees of freedom, which quantify the ratio of variance between groups to variance within groups, and p-values are reported. A high F-value with a corresponding p-value less than 0.05 was considered statistically significant.

The quantitative results are reported as the means of the calculated IQA metrics with 95% confidence intervals, indicating both central tendency and precision.

The analysis was conducted using commercial software Excel (Microsoft, Pota Lato, CA) and Spyder (Python IDE, Anaconda, Austin, TX).

## Results

In this section, we present the quantitative results in Table 3, which reports the mean and 95% confidence interval of the EPI, entropy and gCNR scores, along with the histogram skewness and kurtosis. These results are compared across the conventional B-mode image (VOLUSON Expert 22 ultrasound machine), our proposed algorithm, and 16 photographic tone mapping algorithms applied to 20 HDR-US datasets. To better illustrate the differences among the algorithms and analyze their behaviors in the ultrasound domain, we grouped them based on their performance trends.

**Table 3:** Mean and 95%CI of EPI, entropy and gCNR scores, along with histogram skewness and kurtosis, across different tone-mapping methods.

Algorithm	Histogram						EPI	Entropy	gCNR		
	Near-field		Mid-field		Far-field				(i)	(ii)	(iii)
	Skewness	Kurtosis	Skewness	Kurtosis	Skewness	Kurtosis					
*Proposed algorithm*	symmetric	low	symmetric	low	symmetric	low	0.27 ± 0.02	7.22 ± 0.07	0.66 ± 0.06	0.61 ± 0.08	0.27 ± 0.04
VOLUSON	symmetric	low	symmetric	low	right	low	0.25 ± 0.03	6.85 ± 0.12	0.57 ± 0.06	0.56 ± 0.08	0.23 ± 0.04
Expert 22 [15]											
Chiu [23]	right	high	right	high	right	high	0.86 ± 0.03	5.98 ± 0.02	0.44 ± 0.09	0.43 ± 0.09	0.33 ± 0.07
Ashikhmin [26]	right	low	right	low	right	low	0.75 ± 0.01	7.44 ± 0.03	0.32 ± 0.07	0.36 ± 0.06	0.17 ± 0.02
YeePattanaik [28]	right	high	right	high	right	high	0.26 ± 0.06	6.78 ± 0.04	0.70 ± 0.13	0.75 ± 0.07	0.25 ± 0.11
Bruce [31]	right	high	right	high	right	high	0.24 ± 0.07	7.39 ± 0.04	0.49 ± 0.07	0.44 ± 0.08	0.30 ± 0.05
TumblinTurk [24]	right	high	right	high	right	high	0.62 ± 0.03	3.94 ± 0.11	0.30 ± 0.07	0.33 ± 0.08	0.25 ± 0.07
Pattanaik [18]	left	high	left	low	right	high	0.00 ± 0.00	7.18 ± 0.07	0.91 ± 0.05	0.80 ± 0.08	0.20 ± 0.04
Reinhard [25]	left	high	right	low	right	high	0.00 ± 0.00	6.56 ± 0.07	0.86 ± 0.08	0.73 ± 0.11	0.25 ± 0.05
Drago [19]	left	high	right	low	right	low	0.10 ± 0.02	6.86 ± 0.05	0.83 ± 0.08	0.69 ± 0.11	0.25 ± 0.05
Krawczyk [29]	left	high	right	high	right	high	0.01 ± 0.01	4.22 ± 0.11	1.02 ± 0.35	0.81 ± 0.10	0.18 ± 0.06
Kim [20] [38]	right	high	right	high	right	high	0.26 ± 0.04	6.03 ± 0.05	0.55 ± 0.09	0.47 ± 0.06	0.27 ± 0.05
Madmad [38]	left	high	right	high	right	high	0.64 ± 0.03	1.21 ± 0.70	0.81 ± 0.15	0.77 ± 0.17	0.36 ± 0.07
Durand [27]	symmetric	high	symmetric	high	symmetric	high	0.32 ± 0.02	6.80 ± 0.03	0.74± 0.10	0.73± 0.08	0.28 ± 0.05
Mertens [30]	symmetric	high	symmetric	high	symmetric	high	0.38 ± 0.02	7.10 ± 0.03	0.34 ± 0.08	0.40 ± 0.08	0.17 ± 0.02
Khan18 [21]	left	low	symmetric	low	right	high	0.08 ± 0.02	7.85 ± 0.02	0.65 ± 0.07	0.54 ± 0.08	0.24 ± 0.04
Liang [32]	symmetric	high	symmetric	high	symmetric	high	0.21 ± 0.03	7.24 ± 0.07	0.27 ± 0.03	0.32 ± 0.05	0.19 ± 0.02
Khan20 [22]	left	low	symmetric	low	right	low	0.06 ± 0.02	7.89 ± 0.02	0.67 ± 0.07	0.55 ± 0.08	0.24 ± 0.04

Subsequently, we present the statistical analyses conducted on these quantitative metrics, followed by a sensitivity analysis with respect to the tunable parameters listed in Table 2. Table 4 details the incremental evaluation of our algorithm through the stepwise incorporation of its components, while Tables 5 and 6 show the results obtained by varying parameters *s* in Step 1 and *a* in Step 2, respectively. Tables 7, 8, 9, and 10 report the results of varying the parameter Δ in Step 2, the Gaussian kernel size n×n in Step 3, and the weights *w*_*i*_ and upper bounds *a*_*i*_ of the corresponding set *A*_*i*_ for i=1,⋯,5. The complete numerical results, histograms, and visualization examples are provided in the Appendix.

**Table 4:** Incremental evaluation of the proposed method starts with Step 1 (depth compensation), Step 2 (log-based histogram equalization), and Step 5. Steps 3–5 cannot be computed separately, as Step 3 performs decomposition, Step 4 provides recombination weights, and Step 5 reconstructs the image. The baseline Mertens’ algorithm corresponds to Steps 3 and 5 combined.

Algorithm	Histogram						EPI	Entropy	gCNR		
	Near-field		Mid-field		Far-field				(i)	(ii)	(iii)
	Skewness	Kurtosis	Skewness	Kurtosis	Skewness	Kurtosis					
VOLUSON Expert 22 [15]	symmetric	low	symmetric	low	right	low	0.25 ± 0.03	6.85 ± 0.12	0.57 ± 0.06	0.56 ± 0.08	0.23 ± 0.04
Baseline Mertens	symmetric	high	symmetric	high	symmetric	high	0.38 ± 0.02	7.10 ± 0.03	0.34 ± 0.08	0.40 ± 0.08	0.17 ± 0.02
After step 1	symmetric	high	symmetric	high	symmetric	high	0.26 ± 0.02	7.46 ± 0.05	0.64 ± 0.07	0.53 ± 0.09	0.25 ± 0.05
After step 2	symmetric	low	symmetric	low	symmetric	low	0.26 ± 0.02	7.44 ± 0.04	0.53 ± 0.08	0.50 ± 0.07	0.27 ± 0.04
After step 5	symmetric	low	symmetric	low	symmetric	low	0.27 ± 0.02	7.22 ± 0.07	0.66 ± 0.06	0.61 ± 0.08	0.27 ± 0.04

**Table 5:** Mean with 95%CI of EPI, entropy and gCNR scores, along with histogram skewness and kurtosis, across different values of the parameter *s* in Step 1.

Algorithm	Histogram						EPI	Entropy	gCNR		
	Near-field		Mid-field		Far-field				(i)	(ii)	(iii)
	Skewness	Kurtosis	Skewness	Kurtosis	Skewness	Kurtosis					
VOLUSON Expert 22 [15]	symmetric	low	symmetric	low	right	low	0.25 ± 0.03	6.85 ± 0.12	0.57 ± 0.06	0.56 ± 0.08	0.23 ± 0.04
s = 0	left	low	symmetric	low	right	low	0.29 ± 0.02	7.44 ± 0.04	0.53 ± 0.08	0.50 ± 0.07	0.27 ± 0.04
*s* = 1	symmetric	low	symmetric	low	symmetric	low	0.28 ± 0.02	7.09 ± 0.07	0.68 ± 0.07	0.58 ± 0.08	0.27 ± 0.04
*s* = 2	symmetric	low	symmetric	low	symmetric	low	0.28 ± 0.02	7.17 ± 0.06	0.67 ± 0.05	0.56 ± 0.07	0.27 ± 0.04
*s* = 3	symmetric	low	symmetric	low	symmetric	low	0.28 ± 0.02	7.22 ± 0.08	0.67 ± 0.06	0.56 ± 0.07	0.27 ± 0.04
*s* = 4	symmetric	low	symmetric	low	symmetric	low	0.27 ± 0.02	7.22 ± 0.07	0.66 ± 0.06	0.61 ± 0.08	0.27 ± 0.04

**Table 6:** Mean with 95%CI of EPI, entropy and gCNR scores, along with histogram skewness and kurtosis, across different values of the parameter *a* in Step 2.

Algorithm	Histogram						EPI	Entropy	gCNR		
	Near-field		Mid-field		Far-field				(i)	(ii)	(iii)
	Skewness	Kurtosis	Skewness	Kurtosis	Skewness	Kurtosis					
VOLUSON Expert 22 [15]	symmetric	low	symmetric	low	right	low	0.25 ± 0.03	6.92 ± 0.10	0.59 ± 0.06	0.59 ± 0.08	0.23 ± 0.04
*a* = 0	symmetric	high	symmetric	high	symmetric	high	0.27 ± 0.02	6.68 ± 0.02	0.64 ± 0.09	0.66 ± 0.10	0.27 ± 0.04
*a* = 20	symmetric	low	symmetric	low	symmetric	low	0.27 ± 0.02	7.34 ± 0.03	0.53 ± 0.07	0.44 ± 0.07	0.28 ± 0.04
*a* = 24	symmetric	low	symmetric	low	symmetric	low	0.27 ± 0.02	7.31 ± 0.04	0.64 ± 0.06	0.53 ± 0.08	0.28 ± 0.04
*a* = 26	symmetric	low	symmetric	low	symmetric	low	0.27 ± 0.02	7.22 ± 0.07	0.66 ± 0.06	0.61 ± 0.08	0.27 ± 0.04
*a* = 28	right	low	right	low	right	low	0.27 ± 0.02	7.08 ± 0.12	0.63 ± 0.07	0.57 ± 0.08	0.27 ± 0.04
*a* = 30	right	high	right	high	right	high	0.27 ± 0.02	6.67 ± 0.20	0.70 ± 0.06	0.61 ± 0.08	0.26 ± 0.04

**Table 7:** Mean with 95%CI of EPI, entropy and gCNR scores, along with histogram skewness and kurtosis, across different values of the discretization step size Δ in Step 2.

Algorithm	Histogram						EPI	Entropy	gCNR			Time
	Near-field		Mid-field		Far-field				(i)	(ii)	(iii)	
	Skewness	Kurtosis	Skewness	Kurtosis	Skewness	Kurtosis						
VOLUSON Expert 22 [15]	symmetric	low	symmetric	low	right	low	0.25 ± 0.03	6.92 ± 0.10	0.59 ± 0.06	0.59 ± 0.08	0.23 ± 0.04	-
Δ=0.1	symmetric	low	symmetric	low	symmetric	low	0.27 ± 0.02	7.09 ± 0.13	0.69 ± 0.06	0.61 ± 0.08	0.24 ± 0.05	4.01 ± 0.45
Δ=0.5	symmetric	low	symmetric	low	symmetric	low	0.27 ± 0.02	7.07 ± 0.13	0.67 ± 0.06	0.59 ± 0.08	0.25 ± 0.05	0.97 ± 0.14
Δ=1	symmetric	low	symmetric	low	symmetric	low	0.27 ± 0.02	7.22 ± 0.07	0.66 ± 0.06	0.61 ± 0.08	0.27 ± 0.04	0.53 ± 0.07
Δ=2	symmetric	low	symmetric	low	symmetric	low	0.27 ± 0.02	6.93 ± 0.13	0.60 ± 0.07	0.56 ± 0.06	0.34 ± 0.05	0.35 ± 0.04

**Table 8:** Mean with 95%CI of EPI, entropy and gCNR scores, along with histogram skewness and kurtosis, across different values of the size for Gaussian kernel *G* in Step 3.

Algorithm	Histogram						EPI	Entropy	gCNR			Time
	Near-field		Mid-field		Far-field				(i)	(ii)	(iii)	
	Skewness	Kurtosis	Skewness	Kurtosis	Skewness	Kurtosis						
VOLUSON Expert 22 [15]	symmetric	low	symmetric	low	right	low	0.25 ± 0.03	6.92 ± 0.10	0.59 ± 0.06	0.59 ± 0.08	0.23 ± 0.04	-
3×3	symmetric	low	symmetric	low	symmetric	low	0.27 ± 0.02	7.22 ± 0.07	0.66 ± 0.06	0.61 ± 0.08	0.27 ± 0.04	0.54 ± 0.11
5×5	symmetric	low	symmetric	low	symmetric	low	0.27 ± 0.02	7.22 ± 0.07	0.66 ± 0.06	0.61 ± 0.08	0.27 ± 0.04	0.53 ± 0.07
7×7	symmetric	low	symmetric	low	symmetric	low	0.27 ± 0.02	7.22 ± 0.07	0.66 ± 0.06	0.61 ± 0.08	0.27 ± 0.04	0.64 ± 0.09

**Table 9:** Mean with 95%CI of EPI, entropy and gCNR scores, along with histogram skewness and kurtosis, across different values of the upper bound *a*_*i*_ of the set $A_{i},i=1,\ldots ,4$ in Step 4.

Algorithm	Histogram						EPI	Entropy	gCNR		
	Near-field		Mid-field		Far-field				(i)	(ii)	(iii)
	Skewness	Kurtosis	Skewness	Kurtosis	Skewness	Kurtosis					
VOLUSON Expert 22 [15]	symmetric	low	symmetric	low	right	low	0.25 ± 0.03	6.85 ± 0.12	0.57 ± 0.06	0.56 ± 0.08	0.23 ± 0.04
$a_{1}=\lfloor K/4\rfloor -1$	symmetric	low	symmetric	low	symmetric	low	0.24 ± 0.02	7.14 ± 0.11	0.65 ± 0.07	0.60 ± 0.08	0.25 ± 0.04
$a_{1}=\lfloor K/4\rfloor$	symmetric	low	symmetric	low	symmetric	low	0.27 ± 0.02	7.22 ± 0.07	0.66 ± 0.06	0.61 ± 0.08	0.26 ± 0.04
$a_{1}=\lfloor K/4\rfloor +1$	symmetric	low	symmetric	low	symmetric	low	0.26 ± 0.02	7.07 ± 0.11	0.64 ± 0.06	0.59 ± 0.07	0.26 ± 0.04
$a_{2}=\lfloor K/2\rfloor -1$	symmetric	low	symmetric	low	symmetric	low	0.27 ± 0.02	7.06 ± 0.12	0.63 ± 0.07	0.58 ± 0.07	0.26 ± 0.04
$a_{2}=\lfloor K/2\rfloor$	symmetric	low	symmetric	low	symmetric	low	0.27 ± 0.02	7.22 ± 0.07	0.66 ± 0.06	0.61 ± 0.08	0.27 ± 0.04
$a_{2}=\lfloor K/2\rfloor +1$	symmetric	low	symmetric	low	symmetric	low	0.27 ± 0.02	7.05 ± 0.12	0.64 ± 0.06	0.58 ± 0.07	0.26 ± 0.04
$a_{3}=\lfloor 3K/4\rfloor -2$	symmetric	low	symmetric	low	symmetric	low	0.27 ± 0.02	7.02 ± 0.12	0.60 ± 0.07	0.54 ± 0.07	0.26 ± 0.04
$a_{3}=\lfloor 3K/4\rfloor -1$	symmetric	low	symmetric	low	symmetric	low	0.27 ± 0.02	7.22 ± 0.07	0.66 ± 0.06	0.61 ± 0.08	0.27 ± 0.04
$a_{3}=\lfloor 3K/4\rfloor$	symmetric	low	symmetric	low	symmetric	low	0.26 ± 0.02	7.36 ± 0.07	0.64 ± 0.06	0.54 ± 0.08	0.27 ± 0.05
$a_{4}=\lfloor K/4\rfloor$	symmetric	low	symmetric	low	symmetric	low	0.27 ± 0.02	7.06 ± 0.13	0.62 ± 0.07	0.57 ± 0.07	0.26 ± 0.04
$a_{4}=\lfloor K/4\rfloor +1$	symmetric	low	symmetric	low	symmetric	low	0.27 ± 0.02	7.22 ± 0.07	0.66 ± 0.06	0.61 ± 0.08	0.27 ± 0.04
$a_{4}=\lfloor K/4\rfloor +2$	symmetric	low	symmetric	low	symmetric	low	0.27 ± 0.02	7.07 ± 0.12	0.65 ± 0.06	0.58 ± 0.08	0.26 ± 0.04

**Table 10:** Mean with 95%CI of EPI, entropy and gCNR scores, along with histogram skewness and kurtosis, across different values of weights *w*_*i*_ of the set $A_{i},i=1,\ldots ,4$ in Step 4.

Algorithm	Histogram						EPI	Entropy	gCNR		
	Near-field		Mid-field		Far-field				(i)	(ii)	(iii)
	Skewness	Kurtosis	Skewness	Kurtosis	Skewness	Kurtosis					
VOLUSON Expert 22 [15]	2*symmetric	low	symmetric	low	right	low	0.25 ± 0.03	6.85 ± 0.12	0.57 ± 0.06	0.56 ± 0.08	0.23 ± 0.04
*w*_1_=2	symmetric	low	symmetric	low	symmetric	low	0.26 ± 0.02	7.17 ± 0.12	0.64 ± 0.07	0.59 ± 0.08	0.25 ± 0.04
*w*_1_=2.5	symmetric	low	symmetric	low	symmetric	low	0.27 ± 0.02	7.11 ± 0.12	0.64 ± 0.07	0.58 ± 0.08	0.26 ± 0.04
*w*_1_=3	symmetric	low	symmetric	low	symmetric	low	0.27 ± 0.02	7.22 ± 0.07	0.66 ± 0.06	0.61 ± 0.08	0.27 ± 0.04
*w*_2_=2	symmetric	low	symmetric	low	symmetric	low	0.26 ± 0.02	7.10 ± 0.11	0.66 ± 0.06	0.60 ± 0.07	0.27 ± 0.04
*w*_2_=2.5	symmetric	low	symmetric	low	symmetric	low	0.27 ± 0.02	7.22 ± 0.07	0.66 ± 0.06	0.61 ± 0.08	0.27 ± 0.04
*w*_2_=3	symmetric	low	symmetric	low	symmetric	low	0.28 ± 0.02	6.66 ± 0.73	0.61 ± 0.07	0.56 ± 0.07	0.25 ± 0.04
*w*_3_=2	symmetric	low	symmetric	low	symmetric	low	0.29 ± 0.02	6.98 ± 0.11	0.58 ± 0.07	0.51 ± 0.07	0.26 ± 0.05
*w*_3_=2.5	symmetric	low	symmetric	low	symmetric	low	0.28 ± 0.02	6.71 ± 0.67	0.61 ± 0.07	0.54 ± 0.08	0.26 ± 0.04
*w*_3_=3	symmetric	low	symmetric	low	symmetric	low	0.27 ± 0.02	7.22 ± 0.07	0.66 ± 0.06	0.61 ± 0.08	0.27 ± 0.04
*w*_4_=1	symmetric	low	symmetric	low	symmetric	low	0.27 ± 0.02	6.74 ± 0.63	0.62 ± 0.07	0.57 ± 0.07	0.26 ± 0.04
*w*_4_=1.5	symmetric	low	symmetric	low	symmetric	low	0.27 ± 0.02	7.22 ± 0.07	0.66 ± 0.06	0.61 ± 0.08	0.27 ± 0.04
*w*_4_=2	symmetric	low	symmetric	low	symmetric	low	0.27 ± 0.02	7.07 ± 0.12	0.65 ± 0.06	0.59 ± 0.07	0.26 ± 0.04
*w*_5_=1	symmetric	low	symmetric	low	symmetric	low	0.27 ± 0.02	7.22 ± 0.07	0.66 ± 0.06	0.61 ± 0.08	0.27 ± 0.04
*w*_5_=1.5	symmetric	low	symmetric	low	symmetric	low	0.27 ± 0.02	7.05 ± 0.30	0.63 ± 0.06	0.56 ± 0.07	0.27 ± 0.04
*w*_5_=2	symmetric	low	symmetric	low	symmetric	low	0.27 ± 0.02	7.21 ± 0.12	0.63 ± 0.06	0.55 ± 0.07	0.27 ± 0.04

### Statistical analysis

Repeated-measures ANOVA confirmed statistically significant effects of the tone-mapping algorithm across all evaluated metrics (*p* < 0.001). Greenhouse–Geisser corrections were applied where the sphericity assumption was violated. The corresponding test statistics and effect sizes were as follows: EPI - F(4.62,87.78)=182.4, *p* < 0.001, $\eta_{p}^{2}=0.906$; Entropy - F(17,323)=158.9, *p* < 0.001, $\eta_{p}^{2}=0.893$; gCNR between the fetal kidney and amniotic fluid - F(5.01,95.19)=94.2, *p* < 0.001, $\eta_{p}^{2}=0.832$; gCNR between the far-field object and amniotic fluid - F(4.78,90.82)=118.7, *p* < 0.001, $\eta_{p}^{2}=0.862$; and gCNR between the fetal kidney and adjacent ribs or spines - F(5.22,99.18)=71.5, *p* < 0.001, $\eta_{p}^{2}=0.790$. All effect sizes were large, indicating that algorithm choice accounted for a substantial proportion of the variance in image quality metrics.

Post-hoc Holm–Bonferroni–corrected pairwise comparisons were conducted following significant repeated-measures ANOVAs for each image-quality metric. For the EPI score, our algorithm differed significantly from all algorithms (adjusted *p* < 0.001). For the entropy score, differences with TumblinTurk, Pattanaik, Durand, Drago, YeePattanaik, Mertens, Bruce and Liang were not statistically significant (adjusted *p* = 0.999), and the comparison with Ashikhmin was marginally non-significant (adjusted *p* = 0.156). For the gCNR score between the fetal kidney and amniotic fluid, no significant differences were found with TumblinTurk, Pattanaik, Ashikhmin, Khan18, Khan20, Reinhard, or Bruce (adjusted *p* = 0.999), and a similar pattern was observed for the gCNR between the far-field echoic object and amniotic fluid. For the gCNR between the fetal kidney and adjacent hyperechoic structures, only the comparison with Chiu was non-significant (adjusted *p* = 0.999), while all other comparisons were significant (adjusted *p* < 0.001).

These post-hoc results indicate that the proposed tone-mapping algorithm produced distinct image-quality outcomes across most evaluation metrics, confirming that algorithm selection has a strong influence on image appearance. For the EPI and most gCNR measures, the algorithm’s performance differed significantly from nearly all reference methods, suggesting consistent and characteristic behavior in contrast enhancement. In contrast, the entropy results showed that several tone-mapping methods achieved statistically similar outcomes, indicating comparable levels of tonal variability. Overall, these findings imply that while the proposed algorithm yields stable and distinct image characteristics in most contexts, some existing approaches produce similar results for certain aspects of image characteristics.

#### Incremental study of algorithm steps.

Table 4 presents the incremental evaluation of our algorithm through the stepwise incorporation of its components. Steps 3 and 5, when applied without the other steps, correspond to the baseline Mertens’ algorithm. These two steps cannot be separated, as step 3 involves decomposition, and step 5 combines the components back into the image. We observe that, compared to the conventional B-mode image acquired from a commercial ultrasound (US) machine, Mertens’ algorithm improves EPI and entropy scores but suffers from low contrast, which indicates overexposure.

We then incorporate Step 1, which accounts for signal attenuation. This results in significant improvements contrast between hyperechoic and hypoechoic tissue in the mid-field, as reflected by the improved mean gCNR (iii) score. Additionally, the contrast between echoic and anechoic regions in the mid-field improves, as indicated by the gCNR (i) score. In the far-field, slight improvements in contrast between echoic and anechoic regions are observed, but the contrast remains lower than that of the conventional B-mode image, as shown by the mean gCNR (ii) score. Entropy increases notably, but EPI decreases, suggesting that the data may contain noise.

By incorporating Step 2, which involves log-based histogram division and discarding bins that lack meaningful information, overall contrast are improved. This enhancement is attributed to histogram equalization, which balances the intensity distribution. The effect is evident in the reduced kurtosis of the histogram after including Step 2, compared to the higher kurtosis observed when only Step 1 is applied. However, while the overall contrast improves, the contrast in the far-field still remains below the scores achieved by the conventional B-mode image. The entropy and EPI scores remain largely unchanged, indicating that this step enhances contrast without altering the overall information content or degrading structural integrity.

Finally, to enhance local contrast in the far-field, we introduce depth-aware weights into the fusion process. The entropy score decreases while the EPI increases, implying that the data becomes less noisy. Moreover, this step improves all contrast metrics, outperforming both the baseline Mertens’ algorithm and the conventional B-mode image in terms of overall contrast, local contrast, and tissue differentiation in the mid-field.

#### Parameter sensitivity analysis.

Table 5 presents the results of our algorithm when varying the parameter *s* in Step 1, which determines the choice of the function *p*(*m*) used for depth compensation. As described in the methodology section and illustrated in Fig 9, *p*(*m*) is selected as a piecewise polynomial function, whose order decreased as depth goes larger. Using a fixed polynomial order results in a loss of near-field details, causing the image visually unusable. Here, we analyze the impact of the parameter *s*, which represents the baseline order of the piecewise polynomial function *p* (lowest order *s*, highest order 2*s*.)

The benefits of depth compensation have already been observed in the incremental analysis. Furthermore, here, we observe that using higher-order polynomials for *p*, i.e., selecting *s*>0, improves local contrast between tissues, as well as overall contrast. For all values s=1,2,3,4, we observe an increase in contrast between tissues in the mid-field region, as reflected by higher mean gCNR scores (i) and (iii) in comparison to conventional B-mode images. For contrast improvement in the far-field, only *s* = 1 and *s* = 4 show significant gains. Notably, *s* = 4 performs better in the far-field due to stronger compensation that penetrates deeper. It achieves higher entropy scores, indicating improved detail visibility, particular in the far-field. Since our focus is on obese patients, where fetal details are located deeper into the far-field, we select *s* = 4 as the optimal choice. The enhancement in contrast comes at a trade-off with edge preservation, as reflected by a slight decrease in the EPI score. However, the reduction is minor, and the EPI remains higher than that of the conventional B-mode image, indicating that the overall structural integrity remains well maintained.

Table 6 presents the results of our algorithm when varying the parameter *a* in Step 2, which determines how bins without important information are discarded.

When no bins are discarded (*a* = 0), the final results appear overexposed, as reflected by a symmetric but high-kurtosis histogram. The mean entropy score is also lower compared to that of conventional B-mode images, although we observe improvements in local contrast between echoic and anechoic regions, as well as between hyper- and hypoechoic tissues in both mid-field and far-field. Increasing *a* improves overall contrast and raises entropy scores. However, if *a* is set too high (e.g., *a* = 30), the image becomes underexposed with excessive contrast due to the excessive discarding of information. This is reflected in a significant drop in entropy scores, indicating a loss of important details, and the histogram becomes increasingly right-skewed. Notably, changes in *a* do not affect the EPI, which is consistent with the behavior observed in the incremental analysis when Step 2 was introduced.

We observe that *a* = 26 and *a* = 28 provide the best results, with improvements across all four metrics compared to the conventional B-mode image. Among these, *a* = 26 performs slightly better, with higher scores and more symmetric histogram. The value *a* = 26 corresponds to discarding bins that contains fewer than 10% of the total numbers of pixels. The parameter *a* will vary depends on the dynamic range of the HDR-data that need to be compressed, but the 10% threshold can be consistently used to calculate the corresponding *a*. For a visualized example reflecting the influence of *a* to the tone-mapped procedure, see Fig 10.

Table 7 summarize the effect of varying the discretization size Δ in Step 2. We observe that Δ does not influence edge preservation, as all settings yield nearly identical mean EPI. However, Δ=1 achieves the highest entropy, indicating better retention of fine textures and subtle gray-level variations. Smaller values increase mid-field contrast, raising mean gCNR(i), but reduce tissue differentiation, reflected by lower mean gCNR(iii). Far-field contrast remains stable for Δ≤1 but drops noticeably for Δ=2, which, together with its lower entropy, suggests underexposure.

Histograms are generally symmetric, but smaller Δ produce more centered, Gaussian-like distributions and overall brighter images, whereas larger Δ yields darker results. Setting Δ too high can therefore lead to underexposed images. Computational cost increases for smaller Δ; however, although larger Δ offers a notable speed gain (e.g., Δ=2 is ∼34% faster than Δ=1), image quality is significantly degraded, with Δ=2 performing worse than conventional B-mode images. Overall, Δ=1 provides the best balance between image-quality metrics and computational efficiency. A visual example is shown in Fig 11.

Table 8 and Fig 13 illustrate the impact of different Gaussian kernel sizes on the proposed algorithm. Overall, varying the kernel size does not result in noticeable visual or quantitative changes. All kernel sizes produce images with comparable brightness, contrast, and structural detail, as reflected in similar entropy, EPI, and gCNR values, as well as consistent histogram shapes. Computation times are also similar, with the 5×5 kernel providing the most stable and efficient performance.

Tables 9 and 10 present the effect of weights *w*_*i*_ and upper bounds *a*_*i*_ for the corresponding image sets *A*_*i*_, i=1,…,5. As expected, higher *w*_1_ increases contrast and prevents overexposure. Moreover, setting $w_{2}=w_{3}$ notably reduces contrast, as *w*_3_ contains more mid-field details and should have a higher weight. Similarly, the weights *w*_4_ and *w*_5_ significantly influence contrast in the far-field; adjusting these weights by ±0.5 or shifting the boundaries by ±1 noticeably decreases the gCNR score in that region. Overall, selecting the weights and upper bounds as specified in the Tunable Parameters section yields the best results within the tested range, achieving the highest entropy, EPI, and gCNR scores. Histograms are generally symmetric with low skewness. Deviating from the chosen values—using lower or higher values—produces images with more Gaussian-like, centered histograms and lower peaks, indicating reduced contrast and less optimal preservation of subtle details.

## Discussion

### Comparison with traditional tone mapping techniques

Evaluating traditional TMOs in the context of ultrasound helps identify their strengths and limitations, providing insights to guide the development of tone mapping methods specifically tailored to the unique characteristics of ultrasound images. A comprehensive analysis of these limitations, along with the improvements introduced by our algorithm, is provided below. The analysis reveals that photographic tone mapping algorithms are generally ineffective for ultrasound images, as their outputs often suffer from artifacts and either excessive or insufficient contrast, making them unsuitable for clinical use. The limitations of traditional tone mapping algorithms in the ultrasound domain are summarized in Table 1.

#### Algorithms prone to artifacChiu, Ashikhmin, Yee and Pattanaik, and Bruce.

Common issues in tone-mapping HDR images include the generation of artifacts due to the over-amplification of scattering-induced speckle noise. These artifacts cause unnatural intensity transitions and distort fine anatomical details, ultimately reducing the diagnostic quality of the images. Additionally, histograms often exhibit skewness, indicating poor contrast distribution. As a consequence, such tone-mapping techniques are not directly applicable to ultrasound images, highlighting the need for approaches that address the unique physical properties of ultrasound wave propagation. This often results in one or two unusually high metric scores, while the other evaluation scores remain low. This motivated us to address depth-dependent signal attenuation in our algorithm, specifically in Step 1 and Step 4.

A detailed analysis of each algorithm in this category is provided below:

1. **Chiu’s algorithm** [23] blurs the image to eliminate high frequencies and then inverts the result, aiming to reproduce fine details of the original image. However, when very bright and very dark areas are close to each other, this method produce artifacts called gradient reversal. In images affected by gradient reversal, regions with high gradient, such as bright areas adjacent to dark areas (edges), may appear unnatural. Bright regions may appear darker than expected, while dark regions may appear brighter, leading to distorted edge appearances. For visual examples, see Appendix. These artifacts cause abrupt and unnatural changes in intensity, leading to a distorted image. Moreover, the elimination of high frequencies results in a loss of fine details, reflected in low entropy scores and underexposure, particularly within the fetus. This is further indicated by a extremely high gCNR scores inside fetus and right-skewed histograms. This phenomenon occurs because most of the pixels are turned black, while only the brightest values remain white. This artifact is also reflected by very low entropy but unusually high edge preservation scores, highlighting that edges are exaggerated despite the overall loss of image detail.
2. The results of **Ashikhmin’s algorithm** [26] is unusable due to artifacts caused by the over-enhancement of speckle noise present throughout the processed images. While the details are still present, they are indistinguishable because of these artifacts, which explains the high entropy and EPI, but low gCNR scores. Moreover, the right-skewed histograms suggest that the overall image is still underexposed.
3. Method of **Yee and Pattanaik** [28] is affected by artifacts caused by over-amplification of speckle noise, resulting in a completely distorted image where fine details become indistinguishable. Their algorithm also exhibits excessive contrast, especially in the far-field region: the mean entropy and EPI score is low, while the mean gCNR scores between far-field tissues and fluid are extremely high. Furthermore, the histograms are strongly right-skewed, reflecting a predominance of dark pixels and underexposure.
4. Results from **Bruce’s algorithm** [31] suffer from underexposure, as reflected by a right-skewed histogram. Moreover, the method over-enhances speckle noise and produces halo artifacts around high-contrast edges, making it unsuitable for clinical diagnostics. The halo artifact is also reflected by high entropy but low edge preservation scores.

#### Algorithms prone to excessive contrast: TumblinTurk, Pattanaik, Reinhard, Drago, Krawczyk, Kim, and Madmad.

A prevalent issue in traditional TMOs is excessive contrast, which results in underexposure, overly dark regions, and poor local contrast, thereby degrading image quality and diagnostic value. Many algorithms produce binary-like images with histograms skewed toward extremely dark or bright intensity values, making it difficult to differentiate hypoechoic and hyperechoic tissues. While some methods enhance contrast or tissue differentiation in specific regions, they often fail to preserve subtle fetal details, particularly in the mid-field and far-field areas. This results in low entropy and EPI scores with extremely high gCNR values, indicating significant detail loss and inconsistent brightness distribution, or abnormally low entropy with high EPI, suggesting loss of subtle structures due to over-enhanced edges. To address these issues, our algorithm is designed to include histogram equalization in Step 2, aiming to redistribute intensity values, improve brightness balance, enhance contrast, and preserve anatomical details.

A detailed analysis of each algorithm in this category is provided below:

1. **Tumblin and Turk’s** [24] algorithm only compresses the lowest frequencies of the original signal while preserving high frequencies. This frequency-based algorithm is not affected by artifacts; however, high-frequency details - specifically, fetal and maternal tissues in both the near-field and far-field areas - are not well compressed. As a result, fine details are lost, with both hypoechoic and hyperechoic tissues are represented as white pixels in the final tone-mapped image, making it appear significantly overexposed. While the near-field is dominated by white pixels due to the hyperechoic maternal tissue and the far-field by black pixels due to anechoic amniotic fluid, the mid-field appears as black-and-white. This black-and-white appearance refers to an artifact in which the image’s pixel intensities are exaggerated to extremes, causing bright pixels to appear overly bright and dark pixels overly dark. This creates a strong contrast between regions, resulting in a binary-like appearance instead of natural transitions between shades of gray. These issues are reflected in abnormally low mean entropy with high mean EPI, as well as low mean gCNR scores between echogenic and anechoic regions in the mid- and far-field. In the histograms, pixel intensities in the near-field are concentrated at the far right, while those in the mid- and far-field are strongly right-skewed.
2. In the results of **Pattanaik’s** algorithm [18], fine details within the fetus are barely visible, and the tone-mapped images remain overexposed. These issues are reflected by an EPI score of 0, low entropy, and low mean gCNR scores between adjacent tissues within the fetus. The overexposure is further evidenced by extremely high mean gCNR scores between hyperechoic tissues and fluid in the mid-field and far-field regions, indicating that the gray-level differences approach those of pure black and white. The histograms for the near-field are concentrated at higher intensities, while those for the far-field are concentrated at lower intensities. The histogram of the mid-field is right-skewed with low kurtosis, with peaks at both extremely dark and bright intensity values.
3. **Reinhard’s** algorithm [25] suffers from issues of excessive contrast and loss of fine details, as reflected by low mean entropy and EPI scores, with strong pixel concentrations in the low- and high-intensity bins of the histograms. Specifically, the histograms of the near-field and far-field are strongly skewed, with the near-field concentrated at higher intensities and the far-field concentrated at lower intensities, indicating areas of high contrast.
4. **Drago’s** algorithm [19] suffers from the same issues of overexposure and excessive contrast. Its histograms are left-skewed in the near-field and strongly right-skewed in the mid- and far-field.
5. **Krawczyk’s** algorithm [29] enhances the contrast between edges and their surrounding areas, as indicated by high mean gCNR scores between hyperechoic tissues and fluid. However, it produces images with excessively high contrast, resulting in a loss of fine details—particularly in the mid-field region, where structures adjacent to hyperechoic tissues are no longer visible. The images appear nearly black-and-white, with very low mean entropy, EPI, and poor fetal tissue differentiation scores. These issues are reflected in strongly skewed histograms, strongly dominated by the darkest and brightest pixels.
6. **Kim’s** algorithm [20] produces underexposed images, as evidenced by right-skewed histograms. In the mid-field and far-field, hyperechoic tissues appear too dark, and hypoechoic tissues are completely lost, resulting in lower entropy scores. The local contrast in these regions is reduced, leading to low gCNR scores, especially between tissues and fluid in the mid- and far-field.
7. While enhancing high-intensity values, **Madmad’**s histogram equalization method [38] removed low-intensity values, resulting in a loss of details, especially hyperechoic tissues. The images appear binary. All the pixels belong to either the darkest or the brightest bins in the histogram. The overall contrast is very high, with the near-field area being overexposed, while details in the far field are completely lost. This phenomenon can be observed through an abnormal low mean entropy score and extremely high EPI and gCNR scores between echogenic tissues and fluid. Increasing the number of bins in the equalization process can improve detail preservation and entropy scores a little bit. However, the issue of lost details cannot be resolved, and the contrast remains excessive. This behavior is also expected, since the method was originally designed for X-ray images, where preserving strong edges and high contrast is important for visualizing dense structures, rather than for ultrasound data, which requires careful preservation of subtle details and mid-range intensity variations.

#### Algorithms prone to insufficient contrast: Durand, Mertens, Khan18, Khan20 and Liang.

The third category of image quality issues is insufficient contrast. Unlike excessive contrast, this results in images dominated by mid-tone gray levels with few dark or bright regions, making it difficult to distinguish anechoic and hyperechoic tissues and reducing diagnostic clarity. While some methods enhance sharpness or reduce noise, as reflected by moderately high entropy and EPI, other methods suffer from noisy far-field regions with really high entropy but abnormally low EPI. Moreover, challenges such as overexposure and poor tissue differentiation persist. This issue is often associated with high entropy scores but low gCNR scores, particularly between anechoic and echogenic tissues. Histograms typically exhibit bell-shaped distributions with high kurtosis, characterized by a central peak and a lack of extreme dark or bright values, reflecting a limited intensity range and a flat appearance. To address this issue, we include a depth-dependent weighting process in the multi-resolution procedure in Step 4.

A detailed analysis of each algorithm in this category is provided below:

1. **Durand’s** [27] method is characterized by insufficient contrast. While finer anatomical structures are more visible and distinguishable, the overall images are predominantly composed of mid-gray tones, with minimal dark or bright areas. Anechoic tissues, such as fluid-filled structures, fail to appear black, and hyperechoic tissues, such as bone, fail to appear white. This is reflected in histograms with a high peak in the middle and no bins at the lowest or highest intensity values. Furthermore, the far-field regions often exhibit noise. Nevertheless, the images demonstrate histograms with greater homogeneity.
2. **Mertens’** exposure fusion [30] suffers from the same issue of insufficient contrast as Durand’s method. Anechoic regions appear noisy, and hypoechoic tissues are barely distinguishable, resulting in higher entropy and EPI but extremely low gCNR scores. The histograms of all three regions are concentrated in the center, with no bins at the extreme dark or bright values, leading to a reduced intensity range and making the image appear flat and lacking in contrast.
3. The algorithm of **Khan** [21] results in high overall pixel variability, as indicated by very high mean entropy accompanied by abnormally low EPI, indicating that important structures and edges are not well preserved, and the image appears noisy. The histograms of all three regions are bell-shaped with lower peaks in comparison to all the previous algorithms, indicating that the pixel intensity values are distributed more evenly across the whole intensity range. In the mid-field, however, echogenic tissues are overexposed, and anechoic areas are noisy, resulting in low mean gCNR scores. The noisy mid-field is also reflected in the fairly uniform distribution of its histogram. Additionally, the near-field is excessively overexposed, as indicated by a left-skewed histogram, which makes tissue differentiation within this region more challenging.
4. Results from **Liang’s algorithm** [32] exhibit very low contrast, as reflected by low mean gCNR scores. The histograms are left-skewed, consistent with the bright appearance of Liang’s results. Additionally, the anechoic and hypoechoic regions are overwhelmed by noise, reflected by a lower mean EPI.
5. In the results of **Khan’s** second algorithm [22], issues of overexposed hyperechoic tissues, insufficient contrast, and indistinguishable fetal tissues persist. The mid-field displays a flat distribution, reflected by flattened histograms, while the near-field is excessively overexposed with a left-skewed histogram, and the far-field histogram is right-skewed.

### Our proposed method

#### Comparison to existing algorithms.

Compared to the conventional images acquired from the VOLUSON Expert 22 ultrasound machine, the proposed algorithm enhances contrast resolution, improves tissue differentiation, and reduces noise in the far-field area while preserving fine detail, enabling clearer visualization of fetal structures, better distinction between tissues, and more reliable assessment of subtle anatomical features. This improvement is reflected in the increased gCNR scores between echoic and anechoic tissues in the mid- and far-field, indicating better contrast resolution and reduced noise in the anechoic area; higher gCNR scores between hyperechoic and hypoechoic fetal tissues, reflecting improved tissue differentiation; and higher entropy and EPI scores, demonstrating enhanced preservation of fine details and sharper edges.

Moreover, in contrast to traditional TMOs, when applied to our sample, the proposed method is not prone to artifacts, underexposure, or overexposure, as demonstrated by its higher entropy scores compared to algorithms prone to excessive contrast and higher gCNR scores with moderately high EPI relative to those with insufficient contrast. Additionally, the histograms of the proposed method are non-skewed, indicating a balanced range of pixel intensities without significant bias toward dark or bright regions. The distributions also exhibit lower, broader peaks centered in the middle, reflecting a good dynamic range that captures a wide variety of intensity levels and provides balanced contrast and detail in both shadows and highlights. Building on the strengths and addressing the weaknesses of existing tone-mapping methods, our algorithm improves contrast resolution, tissue differentiation, and fine detail preservation by combining depth-informed compensation, histogram equalization, and weighted multi-resolution fusion. Furthermore, incorporating depth information helps reduce artifacts caused by speckle noise amplification, resulting in outputs showing no visible artifacts in our dataset.

#### Limitations.

Although the proposed algorithm outperforms traditional tone-mapping methods for fetal ultrasound images, it is important to acknowledge its potential limitations and areas for further extension.

Firstly, the current evaluation relies primarily on quantitative metrics, which, while informative, are insufficient for comprehensive clinical validation. An extensive medical study with expert assessments is planned to address this.

Secondly, the algorithm can be applied to different acquisition settings, including varying probes, sector sizes, and regions of interest, though heuristic parameter values may require adjustment.

The dependence on manually tuned parameters may limit ease of use. Future work could implement automatic, learning-based weight optimization using clinical IQA metrics. Additionally, performance should be validated across diverse clinical datasets and patient populations to ensure robustness and reliability. Addressing these challenges will enhance the algorithm’s versatility and broaden its applicability across ultrasound imaging tasks.

The computational complexity of the method may limit real-time applications, especially in resource-constrained environments. It is


O(M×N×log(minM,N)×K),

where *M* and *N* are the input dimensions and *K* is the number of LDR images generated from the HDR-US data. For high-resolution images, this leads to longer processing times; for example, an image of size 1828×,464 pixels required 0.53±0.07 seconds on MATLAB R2024b (Intel i9-12900K CPU, NVIDIA RTX A4000 GPU). This relatively high runtime is due to the segmentation and multi-resolution fusion steps, which is typical for tone-mapping operators in this category (Table 1).

## Conclusion

In conclusion, we present a novel ultrasound-specific tone mapping algorithm that integrates depth compensation, multi-resolution processing, and optimized weighted fusion to enhance contrast resolution while preserving detail. Our approach is specifically designed to address the unique challenges of ultrasound imaging, particularly for patients with increased adipose tissue thickness, where thicker adipose tissue leads to stronger signal attenuation. Depth compensation progressively enhances image intensity in deeper regions to address signal attenuation, while histogram-based partitioning enables adaptive processing of distinct tissue types, such as hyperechoic and hypoechoic regions. A multi-resolution approach captures both coarse and fine details, improving subtle anatomical features without compromising the overall structure of the image. Finally, depth-adaptive weighting is incorporated into the multi-resolution fusion strategy to suppress noise and preserve critical details in deeper tissues, accounting for depth-dependent signal attenuation and noise amplification in the far-field.

From our results, we address two key open questions. First, we find that tone mapping operators designed for photography are unsuitable for ultrasound images, due to the fact that light intensity in photographic images is depth-invariant, whereas ultrasound images experience signal loss with increasing depth. As a result, it is essential to incorporate depth compensation into the tone mapping process to address this attenuation. Second, our methodology proves to be robust and demonstrates improved image quality compared to existing tone mapping methods, highlighting its potential to enhance ultrasound imaging and diagnostic utility.

The next phase of our work focuses on the clinical evaluation of the proposed methods by medical professionals at the hospital. This assessment aims to determine the practical applicability and effectiveness of our techniques in real-world medical scenarios. The clinicians’ evaluation of image quality is already underway to ensure the methods meet the specific needs and challenges of clinical practice. Additionally, we plan to investigate learning-based weight tuning to automate parameter optimization, and to explore real-time implementation to improve computational efficiency. The impact of the display monitor on tone-mapping HDR-US images will also be assessed. The results of these evaluations will be reported in a future publication, providing critical feedback to refine our approach and supporting its potential for broader adoption in medical imaging workflows.

## Supporting information

S1 Table Abbreviations. (PNG)

S1 File Full quantitative results for different TMOs. (XLSX)

S2 File Full sensitivity analysis results for tuneable parameters. (XLSX)

S1 Fig Results of different TMOS applied to image 563. Left to right, top to bottom: image from VOLUSON Expert 22, our proposed method, Artifacts: Chiu, Ashikhmin local, YeePattanaik applied on Tumblin, Bruce; Overexcessive contrast: TumblinTurk, Pattanaik, Reinhard, Drago, Krawczyk, Kim, Madmad; Insufficient contrast: Durand, Mertens, Khan18, Liang, Khan20.
(TIFF)

S2 Fig Histograms of different TMOS results applied to image 563. Left to right, top to bottom: image from VOLUSON Expert 22, our proposed method, Artifacts: Chiu, Ashikhmin local, YeePattanaik applied on Tumblin, Bruce; Overexcessive contrast: TumblinTurk, Pattanaik, Reinhard, Drago, Krawczyk, Kim, Madmad; Insufficient contrast: Durand, Mertens, Khan18, Liang, Khan20.
(TIFF)

S3 Fig Results of different TMOS applied to image 564. Left to right, top to bottom: image from VOLUSON Expert 22, our proposed method, Artifacts: Chiu, Ashikhmin local, YeePattanaik applied on Tumblin, Bruce; Overexcessive contrast: TumblinTurk, Pattanaik, Reinhard, Drago, Krawczyk, Kim, Madmad; Insufficient contrast: Durand, Mertens, Khan18, Liang, Khan20.
(TIFF)

S4 Fig Histograms of different TMOS results applied to image 564. Left to right, top to bottom: image from VOLUSON Expert 22, our proposed method, Artifacts: Chiu, Ashikhmin local, YeePattanaik applied on Tumblin, Bruce; Overexcessive contrast: TumblinTurk, Pattanaik, Reinhard, Drago, Krawczyk, Kim, Madmad; Insufficient contrast: Durand, Mertens, Khan18, Liang, Khan20.
(TIFF)

S5 Fig Results of different TMOS applied to image 573. Left to right, top to bottom: image from VOLUSON Expert 22, our proposed method, Artifacts: Chiu, Ashikhmin local, YeePattanaik applied on Tumblin, Bruce; Overexcessive contrast: TumblinTurk, Pattanaik, Reinhard, Drago, Krawczyk, Kim, Madmad; Insufficient contrast: Durand, Mertens, Khan18, Liang, Khan20.
(TIFF)

S6 Fig Histograms of different TMOS results applied to image 573. Left to right, top to bottom: image from VOLUSON Expert 22, our proposed method, Artifacts: Chiu, Ashikhmin local, YeePattanaik applied on Tumblin, Bruce; Overexcessive contrast: TumblinTurk, Pattanaik, Reinhard, Drago, Krawczyk, Kim, Madmad; Insufficient contrast: Durand, Mertens, Khan18, Liang, Khan20.
(TIFF)

S7 Fig Results of different TMOS applied to image 593. Left to right, top to bottom: image from VOLUSON Expert 22, our proposed method, Artifacts: Chiu, Ashikhmin local, YeePattanaik applied on Tumblin, Bruce; Overexcessive contrast: TumblinTurk, Pattanaik, Reinhard, Drago, Krawczyk, Kim, Madmad; Insufficient contrast: Durand, Mertens, Khan18, Liang, Khan20.
(TIFF)

S8 Fig Histograms of different TMOS results applied to image 593. Left to right, top to bottom: image from VOLUSON Expert 22, our proposed method, Artifacts: Chiu, Ashikhmin local, YeePattanaik applied on Tumblin, Bruce; Overexcessive contrast: TumblinTurk, Pattanaik, Reinhard, Drago, Krawczyk, Kim, Madmad; Insufficient contrast: Durand, Mertens, Khan18, Liang, Khan20.
(TIFF)

S9 Fig Results of different TMOS applied to image 598. Left to right, top to bottom: image from VOLUSON Expert 22, our proposed method, Artifacts: Chiu, Ashikhmin local, YeePattanaik applied on Tumblin, Bruce; Overexcessive contrast: TumblinTurk, Pattanaik, Reinhard, Drago, Krawczyk, Kim, Madmad; Insufficient contrast: Durand, Mertens, Khan18, Liang, Khan20.
(TIFF)

S10 Fig Histograms of different TMOS results applied to image 598. Left to right, top to bottom: image from VOLUSON Expert 22, our proposed method, Artifacts: Chiu, Ashikhmin local, YeePattanaik applied on Tumblin, Bruce; Overexcessive contrast: TumblinTurk, Pattanaik, Reinhard, Drago, Krawczyk, Kim, Madmad; Insufficient contrast: Durand, Mertens, Khan18, Liang, Khan20.
(TIFF)

S11 Fig Results of different TMOS applied to image 604. Left to right, top to bottom: image from VOLUSON Expert 22, our proposed method, Artifacts: Chiu, Ashikhmin local, YeePattanaik applied on Tumblin, Bruce; Overexcessive contrast: TumblinTurk, Pattanaik, Reinhard, Drago, Krawczyk, Kim, Madmad; Insufficient contrast: Durand, Mertens, Khan18, Liang, Khan20.
(TIFF)

S12 Fig Histograms of different TMOS results applied to image 604. Left to right, top to bottom: image from VOLUSON Expert 22, our proposed method, Artifacts: Chiu, Ashikhmin local, YeePattanaik applied on Tumblin, Bruce; Overexcessive contrast: TumblinTurk, Pattanaik, Reinhard, Drago, Krawczyk, Kim, Madmad; Insufficient contrast: Durand, Mertens, Khan18, Liang, Khan20.
(TIFF)

S13 Fig Results of different TMOS applied to image 622. Left to right, top to bottom: image from VOLUSON Expert 22, our proposed method, Artifacts: Chiu, Ashikhmin local, YeePattanaik applied on Tumblin, Bruce; Overexcessive contrast: TumblinTurk, Pattanaik, Reinhard, Drago, Krawczyk, Kim, Madmad; Insufficient contrast: Durand, Mertens, Khan18, Liang, Khan20.
(TIFF)

S14 Fig Histograms of different TMOS results applied to image 622. Left to right, top to bottom: image from VOLUSON Expert 22, our proposed method, Artifacts: Chiu, Ashikhmin local, YeePattanaik applied on Tumblin, Bruce; Overexcessive contrast: TumblinTurk, Pattanaik, Reinhard, Drago, Krawczyk, Kim, Madmad; Insufficient contrast: Durand, Mertens, Khan18, Liang, Khan20.
(TIFF)

S15 Fig Results of different TMOS applied to image 626. Left to right, top to bottom: image from VOLUSON Expert 22, our proposed method, Artifacts: Chiu, Ashikhmin local, YeePattanaik applied on Tumblin, Bruce; Overexcessive contrast: TumblinTurk, Pattanaik, Reinhard, Drago, Krawczyk, Kim, Madmad; Insufficient contrast: Durand, Mertens, Khan18, Liang, Khan20.
(TIFF)

S16 Fig Histograms of different TMOS results applied to image 626. Left to right, top to bottom: image from VOLUSON Expert 22, our proposed method, Artifacts: Chiu, Ashikhmin local, YeePattanaik applied on Tumblin, Bruce; Overexcessive contrast: TumblinTurk, Pattanaik, Reinhard, Drago, Krawczyk, Kim, Madmad; Insufficient contrast: Durand, Mertens, Khan18, Liang, Khan20.
(TIFF)

S17 Fig Results of different TMOS applied to image 636. Left to right, top to bottom: image from VOLUSON Expert 22, our proposed method, Artifacts: Chiu, Ashikhmin local, YeePattanaik applied on Tumblin, Bruce; Overexcessive contrast: TumblinTurk, Pattanaik, Reinhard, Drago, Krawczyk, Kim, Madmad; Insufficient contrast: Durand, Mertens, Khan18, Liang, Khan20.
(TIFF)

S18 Fig Histograms of different TMOS results applied to image 636. Left to right, top to bottom: image from VOLUSON Expert 22, our proposed method, Artifacts: Chiu, Ashikhmin local, YeePattanaik applied on Tumblin, Bruce; Overexcessive contrast: TumblinTurk, Pattanaik, Reinhard, Drago, Krawczyk, Kim, Madmad; Insufficient contrast: Durand, Mertens, Khan18, Liang, Khan20.
(TIFF)

S19 Fig Results of different TMOS applied to image 643. Left to right, top to bottom: image from VOLUSON Expert 22, our proposed method, Artifacts: Chiu, Ashikhmin local, YeePattanaik applied on Tumblin, Bruce; Overexcessive contrast: TumblinTurk, Pattanaik, Reinhard, Drago, Krawczyk, Kim, Madmad; Insufficient contrast: Durand, Mertens, Khan18, Liang, Khan20.
(TIFF)

S20 Fig Histograms of different TMOS results applied to image 643. Left to right, top to bottom: image from VOLUSON Expert 22, our proposed method, Artifacts: Chiu, Ashikhmin local, YeePattanaik applied on Tumblin, Bruce; Overexcessive contrast: TumblinTurk, Pattanaik, Reinhard, Drago, Krawczyk, Kim, Madmad; Insufficient contrast: Durand, Mertens, Khan18, Liang, Khan20.
(TIFF)

S21 Fig Results of different TMOS applied to image 658. Left to right, top to bottom: image from VOLUSON Expert 22, our proposed method, Artifacts: Chiu, Ashikhmin local, YeePattanaik applied on Tumblin, Bruce; Overexcessive contrast: TumblinTurk, Pattanaik, Reinhard, Drago, Krawczyk, Kim, Madmad; Insufficient contrast: Durand, Mertens, Khan18, Liang, Khan20.
(TIFF)

S22 Fig Histograms of different TMOS results applied to image 658. Left to right, top to bottom: image from VOLUSON Expert 22, our proposed method, Artifacts: Chiu, Ashikhmin local, YeePattanaik applied on Tumblin, Bruce; Overexcessive contrast: TumblinTurk, Pattanaik, Reinhard, Drago, Krawczyk, Kim, Madmad; Insufficient contrast: Durand, Mertens, Khan18, Liang, Khan20.
(TIFF)

S23 Fig Results of different TMOS applied to image 677. Left to right, top to bottom: image from VOLUSON Expert 22, our proposed method, Artifacts: Chiu, Ashikhmin local, YeePattanaik applied on Tumblin, Bruce; Overexcessive contrast: TumblinTurk, Pattanaik, Reinhard, Drago, Krawczyk, Kim, Madmad; Insufficient contrast: Durand, Mertens, Khan18, Liang, Khan20.
(TIFF)

S24 Fig Histograms of different TMOS results applied to image 677. Left to right, top to bottom: image from VOLUSON Expert 22, our proposed method, Artifacts: Chiu, Ashikhmin local, YeePattanaik applied on Tumblin, Bruce; Overexcessive contrast: TumblinTurk, Pattanaik, Reinhard, Drago, Krawczyk, Kim, Madmad; Insufficient contrast: Durand, Mertens, Khan18, Liang, Khan20.
(TIFF)

S25 Fig Results of different TMOS applied to image 685. Left to right, top to bottom: image from VOLUSON Expert 22, our proposed method, Artifacts: Chiu, Ashikhmin local, YeePattanaik applied on Tumblin, Bruce; Overexcessive contrast: TumblinTurk, Pattanaik, Reinhard, Drago, Krawczyk, Kim, Madmad; Insufficient contrast: Durand, Mertens, Khan18, Liang, Khan20.
(TIFF)

S26 Fig Histograms of different TMOS results applied to image 685. Left to right, top to bottom: image from VOLUSON Expert 22, our proposed method, Artifacts: Chiu, Ashikhmin local, YeePattanaik applied on Tumblin, Bruce; Overexcessive contrast: TumblinTurk, Pattanaik, Reinhard, Drago, Krawczyk, Kim, Madmad; Insufficient contrast: Durand, Mertens, Khan18, Liang, Khan20.
(TIFF)

S27 Fig Results of different TMOS applied to image 692. Left to right, top to bottom: image from VOLUSON Expert 22, our proposed method, Artifacts: Chiu, Ashikhmin local, YeePattanaik applied on Tumblin, Bruce; Overexcessive contrast: TumblinTurk, Pattanaik, Reinhard, Drago, Krawczyk, Kim, Madmad; Insufficient contrast: Durand, Mertens, Khan18, Liang, Khan20.
(TIFF)

S28 Fig Histograms of different TMOS results applied to image 692. Left to right, top to bottom: image from VOLUSON Expert 22, our proposed method, Artifacts: Chiu, Ashikhmin local, YeePattanaik applied on Tumblin, Bruce; Overexcessive contrast: TumblinTurk, Pattanaik, Reinhard, Drago, Krawczyk, Kim, Madmad; Insufficient contrast: Durand, Mertens, Khan18, Liang, Khan20.
(TIFF)

S29 Fig Results of different TMOS applied to image 698. Left to right, top to bottom: image from VOLUSON Expert 22, our proposed method, Artifacts: Chiu, Ashikhmin local, YeePattanaik applied on Tumblin, Bruce; Overexcessive contrast: TumblinTurk, Pattanaik, Reinhard, Drago, Krawczyk, Kim, Madmad; Insufficient contrast: Durand, Mertens, Khan18, Liang, Khan20.
(TIFF)

S30 Fig Histograms of different TMOS results applied to image 698. Left to right, top to bottom: image from VOLUSON Expert 22, our proposed method, Artifacts: Chiu, Ashikhmin local, YeePattanaik applied on Tumblin, Bruce; Overexcessive contrast: TumblinTurk, Pattanaik, Reinhard, Drago, Krawczyk, Kim, Madmad; Insufficient contrast: Durand, Mertens, Khan18, Liang, Khan20.
(TIFF)

S31 Fig Results of different TMOS applied to image 705. Left to right, top to bottom: image from VOLUSON Expert 22, our proposed method, Artifacts: Chiu, Ashikhmin local, YeePattanaik applied on Tumblin, Bruce; Overexcessive contrast: TumblinTurk, Pattanaik, Reinhard, Drago, Krawczyk, Kim, Madmad; Insufficient contrast: Durand, Mertens, Khan18, Liang, Khan20.
(TIFF)

S32 Fig Histograms of different TMOS results applied to image 705. Left to right, top to bottom: image from VOLUSON Expert 22, our proposed method, Artifacts: Chiu, Ashikhmin local, YeePattanaik applied on Tumblin, Bruce; Overexcessive contrast: TumblinTurk, Pattanaik, Reinhard, Drago, Krawczyk, Kim, Madmad; Insufficient contrast: Durand, Mertens, Khan18, Liang, Khan20.
(TIFF)

S33 Fig Results of different TMOS applied to image 717. Left to right, top to bottom: image from VOLUSON Expert 22, our proposed method, Artifacts: Chiu, Ashikhmin local, YeePattanaik applied on Tumblin, Bruce; Overexcessive contrast: TumblinTurk, Pattanaik, Reinhard, Drago, Krawczyk, Kim, Madmad; Insufficient contrast: Durand, Mertens, Khan18, Liang, Khan20.
(TIFF)

S34 Fig Histograms of different TMOS results applied to image 717. Left to right, top to bottom: image from VOLUSON Expert 22, our proposed method, Artifacts: Chiu, Ashikhmin local, YeePattanaik applied on Tumblin, Bruce; Overexcessive contrast: TumblinTurk, Pattanaik, Reinhard, Drago, Krawczyk, Kim, Madmad; Insufficient contrast: Durand, Mertens, Khan18, Liang, Khan20.
(TIFF)

S35 Fig Results of different TMOS applied to image 724. Left to right, top to bottom: image from VOLUSON Expert 22, our proposed method, Artifacts: Chiu, Ashikhmin local, YeePattanaik applied on Tumblin, Bruce; Overexcessive contrast: TumblinTurk, Pattanaik, Reinhard, Drago, Krawczyk, Kim, Madmad; Insufficient contrast: Durand, Mertens, Khan18, Liang, Khan20.
(TIFF)

S36 Fig Histograms of different TMOS results applied to image 724. Left to right, top to bottom: image from VOLUSON Expert 22, our proposed method, Artifacts: Chiu, Ashikhmin local, YeePattanaik applied on Tumblin, Bruce; Overexcessive contrast: TumblinTurk, Pattanaik, Reinhard, Drago, Krawczyk, Kim, Madmad; Insufficient contrast: Durand, Mertens, Khan18, Liang, Khan20.
(TIFF)

S37 Fig Results of different TMOS applied to image 734. Left to right, top to bottom: image from VOLUSON Expert 22, our proposed method, Artifacts: Chiu, Ashikhmin local, YeePattanaik applied on Tumblin, Bruce; Overexcessive contrast: TumblinTurk, Pattanaik, Reinhard, Drago, Krawczyk, Kim, Madmad; Insufficient contrast: Durand, Mertens, Khan18, Liang, Khan20.
(TIFF)

S38 Fig Histograms of different TMOS results applied to image 734. Left to right, top to bottom: image from VOLUSON Expert 22, our proposed method, Artifacts: Chiu, Ashikhmin local, YeePattanaik applied on Tumblin, Bruce; Overexcessive contrast: TumblinTurk, Pattanaik, Reinhard, Drago, Krawczyk, Kim, Madmad; Insufficient contrast: Durand, Mertens, Khan18, Liang, Khan20.
(TIFF)

S39 Fig Results of different TMOS applied to image 743. Left to right, top to bottom: image from VOLUSON Expert 22, our proposed method, Artifacts: Chiu, Ashikhmin local, YeePattanaik applied on Tumblin, Bruce; Overexcessive contrast: TumblinTurk, Pattanaik, Reinhard, Drago, Krawczyk, Kim, Madmad; Insufficient contrast: Durand, Mertens, Khan18, Liang, Khan20.
(TIFF)

S40 Fig Histograms of different TMOS results applied to image 743. Left to right, top to bottom: image from VOLUSON Expert 22, our proposed method, Artifacts: Chiu, Ashikhmin local, YeePattanaik applied on Tumblin, Bruce; Overexcessive contrast: TumblinTurk, Pattanaik, Reinhard, Drago, Krawczyk, Kim, Madmad; Insufficient contrast: Durand, Mertens, Khan18, Liang, Khan20.
(TIFF)
