# Supplementary figures and images for: Multi-resolution tone mapping for high dynamic range medical ultrasound images
Source: PLoS One. 2026 Jan 20;21(1):e0340777. doi: 10.1371/journal.pone.0340777 (PMC12818761; doi:10.1371/journal.pone.0340777)

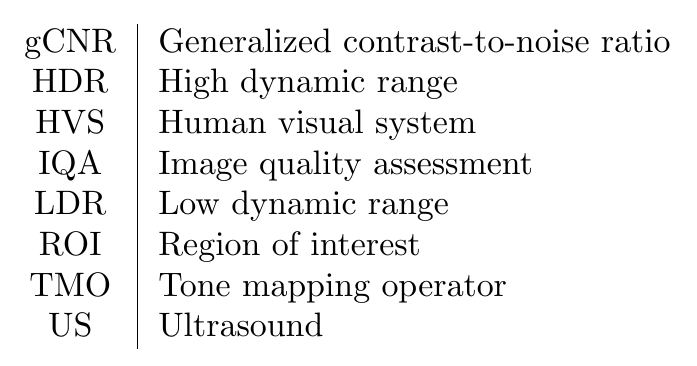

Supplement: S1 Table — (PNG) [file pone.0340777.s001.png]

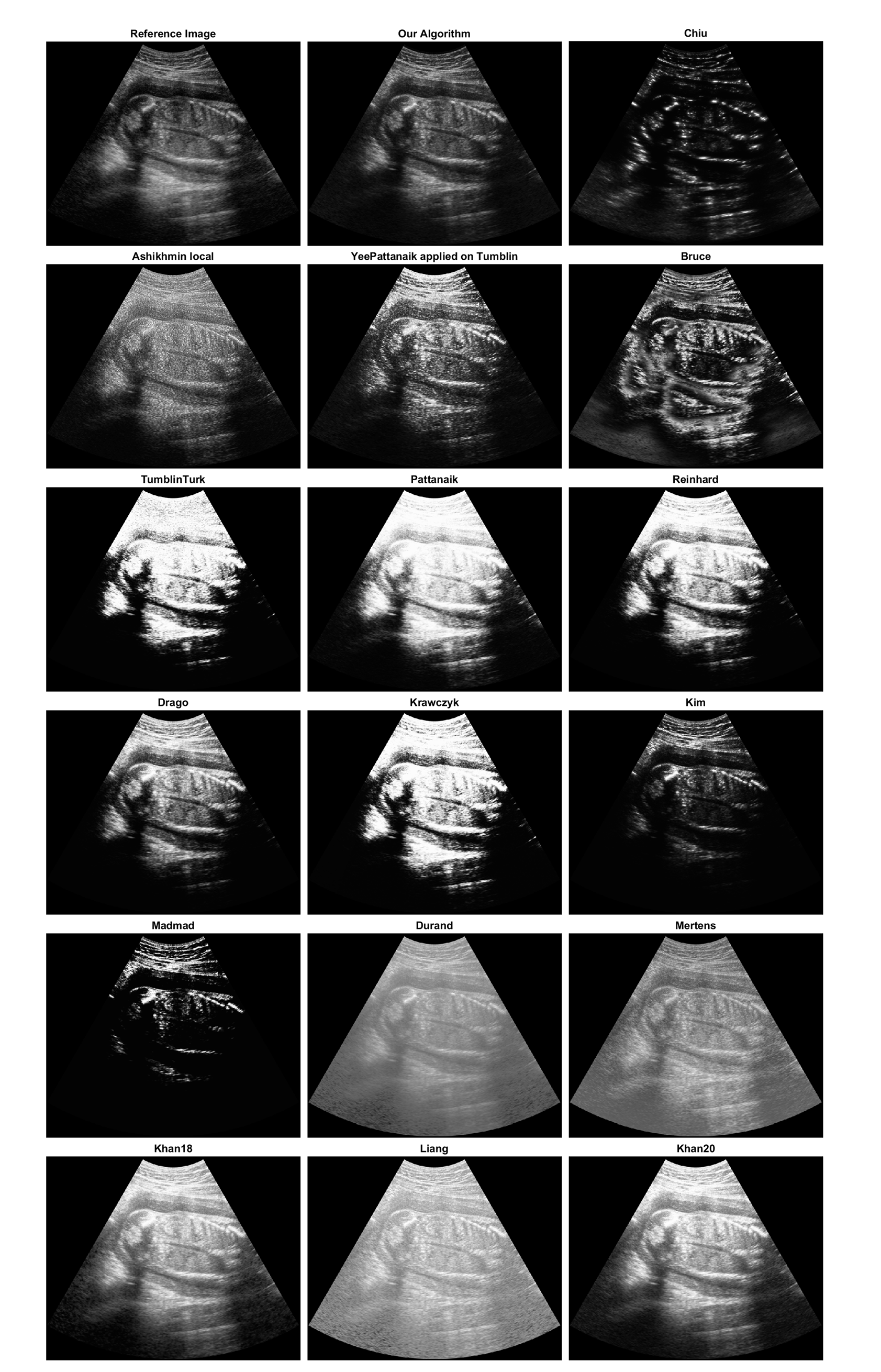

Supplement: S1 Fig — Left to right, top to bottom: image from VOLUSON Expert 22, our proposed method, Artifacts: Chiu, Ashikhmin local, YeePattanaik applied on Tumblin, Bruce; Overexcessive contrast: TumblinTurk, Pattanaik, Reinhard, Drago, Krawczyk, Kim, Madmad; Insufficient contrast: Durand, Mertens, Khan18, Liang, Khan20. (TIFF) [file pone.0340777.s004.tif]

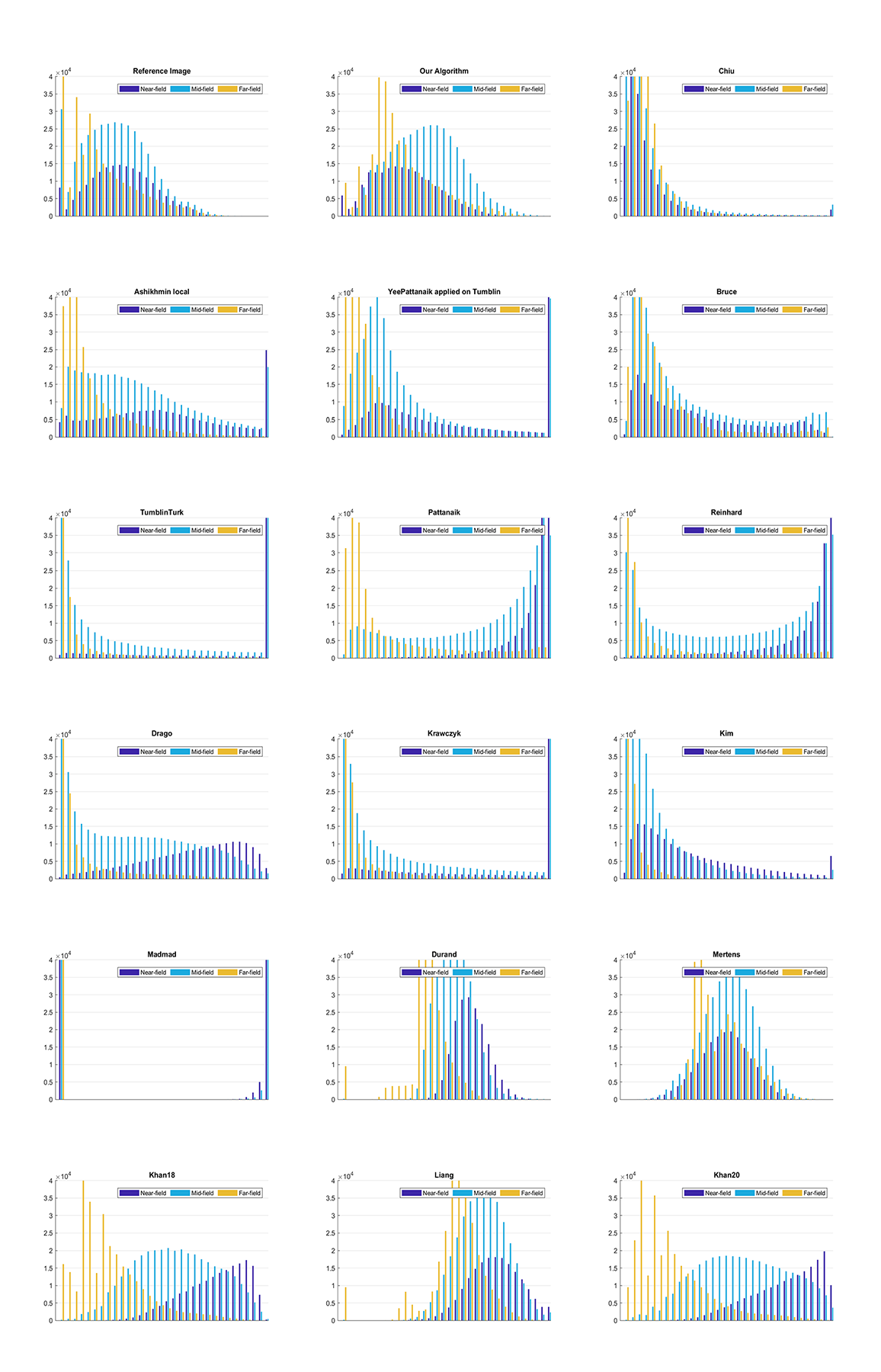

Supplement: S2 Fig — Left to right, top to bottom: image from VOLUSON Expert 22, our proposed method, Artifacts: Chiu, Ashikhmin local, YeePattanaik applied on Tumblin, Bruce; Overexcessive contrast: TumblinTurk, Pattanaik, Reinhard, Drago, Krawczyk, Kim, Madmad; Insufficient contrast: Durand, Mertens, Khan18, Liang, Khan20. (TIFF) [file pone.0340777.s005.tif]

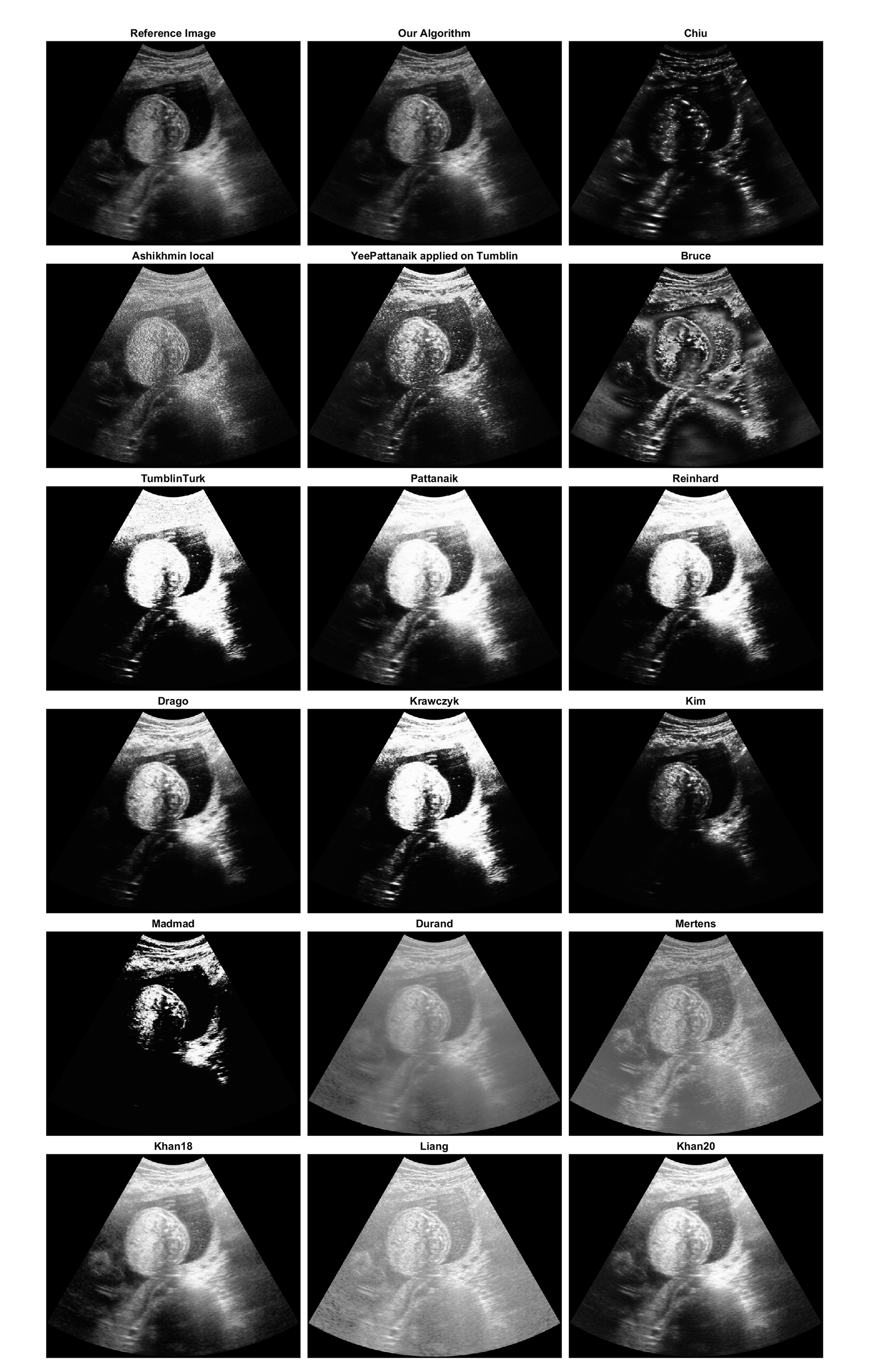

Supplement: S3 Fig — Left to right, top to bottom: image from VOLUSON Expert 22, our proposed method, Artifacts: Chiu, Ashikhmin local, YeePattanaik applied on Tumblin, Bruce; Overexcessive contrast: TumblinTurk, Pattanaik, Reinhard, Drago, Krawczyk, Kim, Madmad; Insufficient contrast: Durand, Mertens, Khan18, Liang, Khan20. (TIFF) [file pone.0340777.s006.tif]

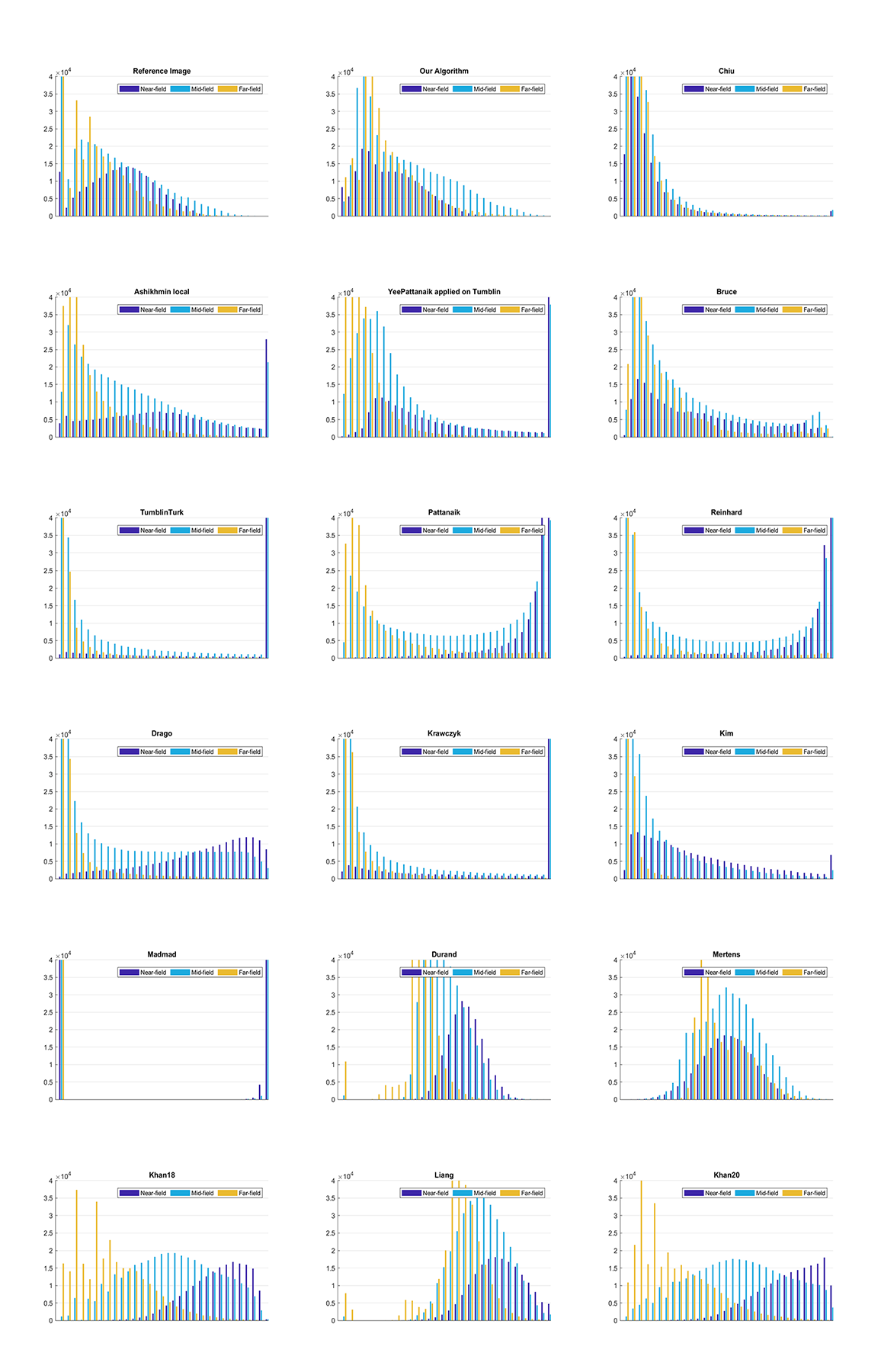

Supplement: S4 Fig — Left to right, top to bottom: image from VOLUSON Expert 22, our proposed method, Artifacts: Chiu, Ashikhmin local, YeePattanaik applied on Tumblin, Bruce; Overexcessive contrast: TumblinTurk, Pattanaik, Reinhard, Drago, Krawczyk, Kim, Madmad; Insufficient contrast: Durand, Mertens, Khan18, Liang, Khan20. (TIFF) [file pone.0340777.s007.tif]

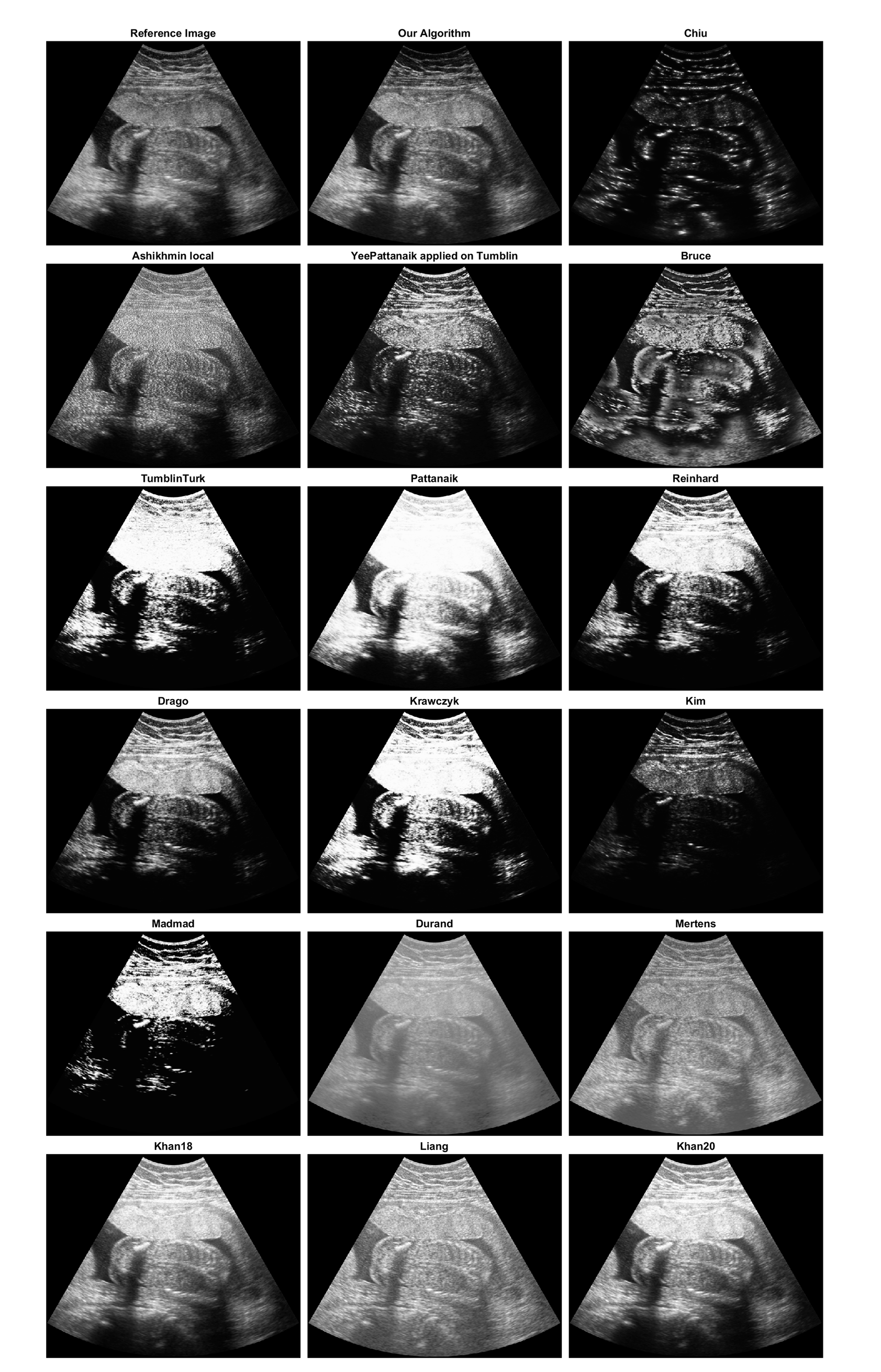

Supplement: S5 Fig — Left to right, top to bottom: image from VOLUSON Expert 22, our proposed method, Artifacts: Chiu, Ashikhmin local, YeePattanaik applied on Tumblin, Bruce; Overexcessive contrast: TumblinTurk, Pattanaik, Reinhard, Drago, Krawczyk, Kim, Madmad; Insufficient contrast: Durand, Mertens, Khan18, Liang, Khan20. (TIFF) [file pone.0340777.s008.tif]

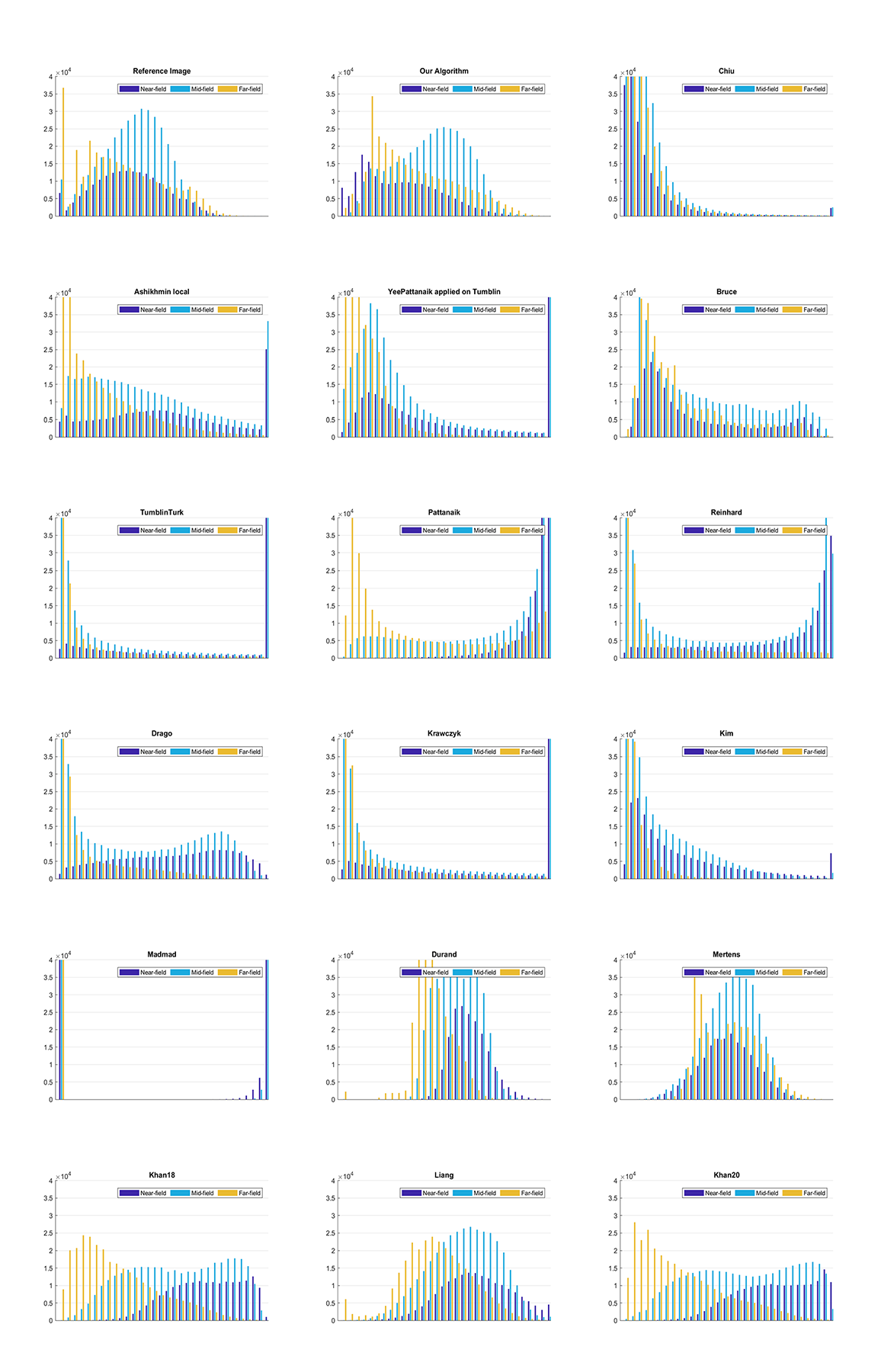

Supplement: S6 Fig — Left to right, top to bottom: image from VOLUSON Expert 22, our proposed method, Artifacts: Chiu, Ashikhmin local, YeePattanaik applied on Tumblin, Bruce; Overexcessive contrast: TumblinTurk, Pattanaik, Reinhard, Drago, Krawczyk, Kim, Madmad; Insufficient contrast: Durand, Mertens, Khan18, Liang, Khan20. (TIFF) [file pone.0340777.s009.tif]

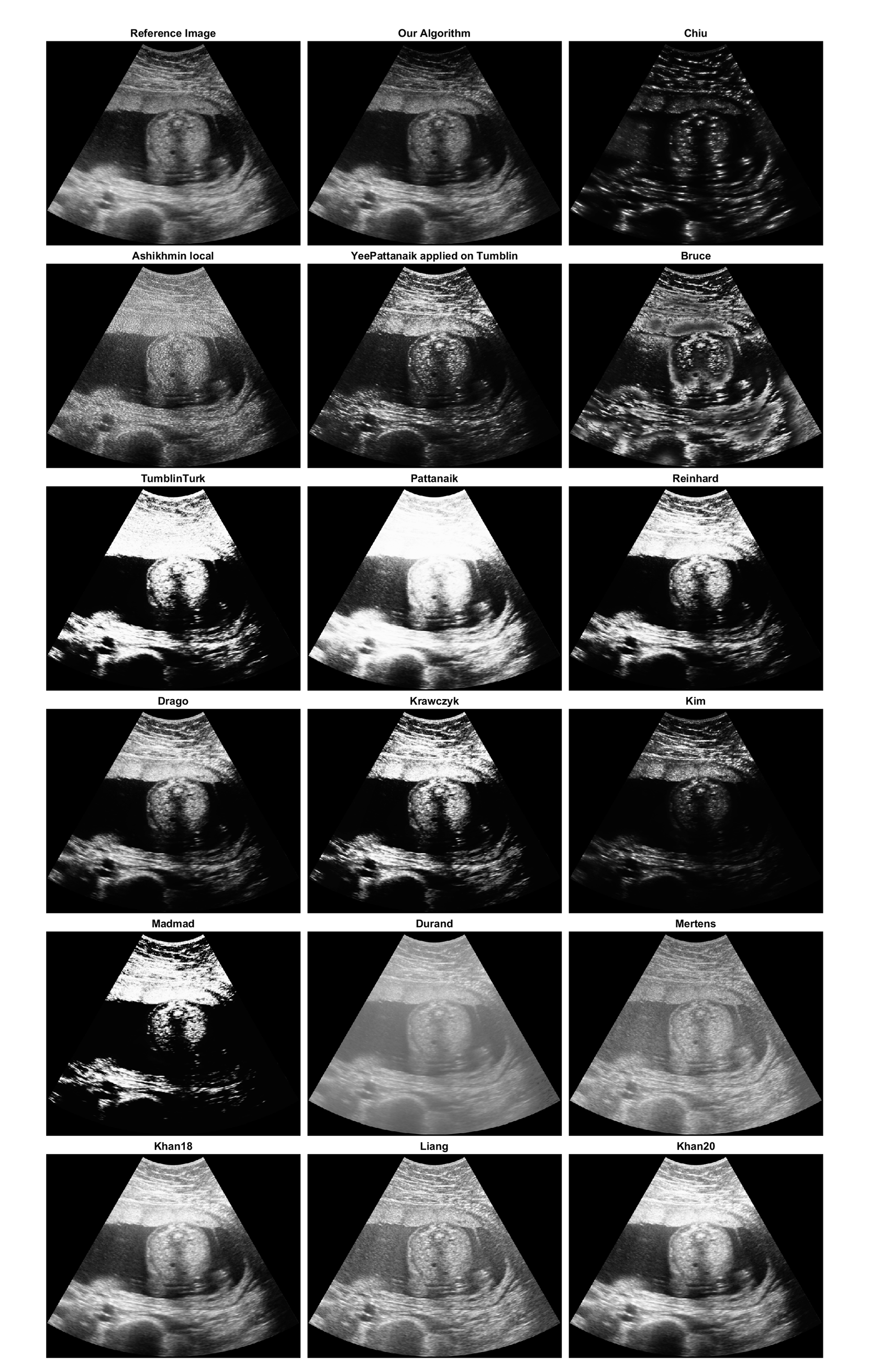

Supplement: S7 Fig — Left to right, top to bottom: image from VOLUSON Expert 22, our proposed method, Artifacts: Chiu, Ashikhmin local, YeePattanaik applied on Tumblin, Bruce; Overexcessive contrast: TumblinTurk, Pattanaik, Reinhard, Drago, Krawczyk, Kim, Madmad; Insufficient contrast: Durand, Mertens, Khan18, Liang, Khan20. (TIFF) [file pone.0340777.s010.tif]

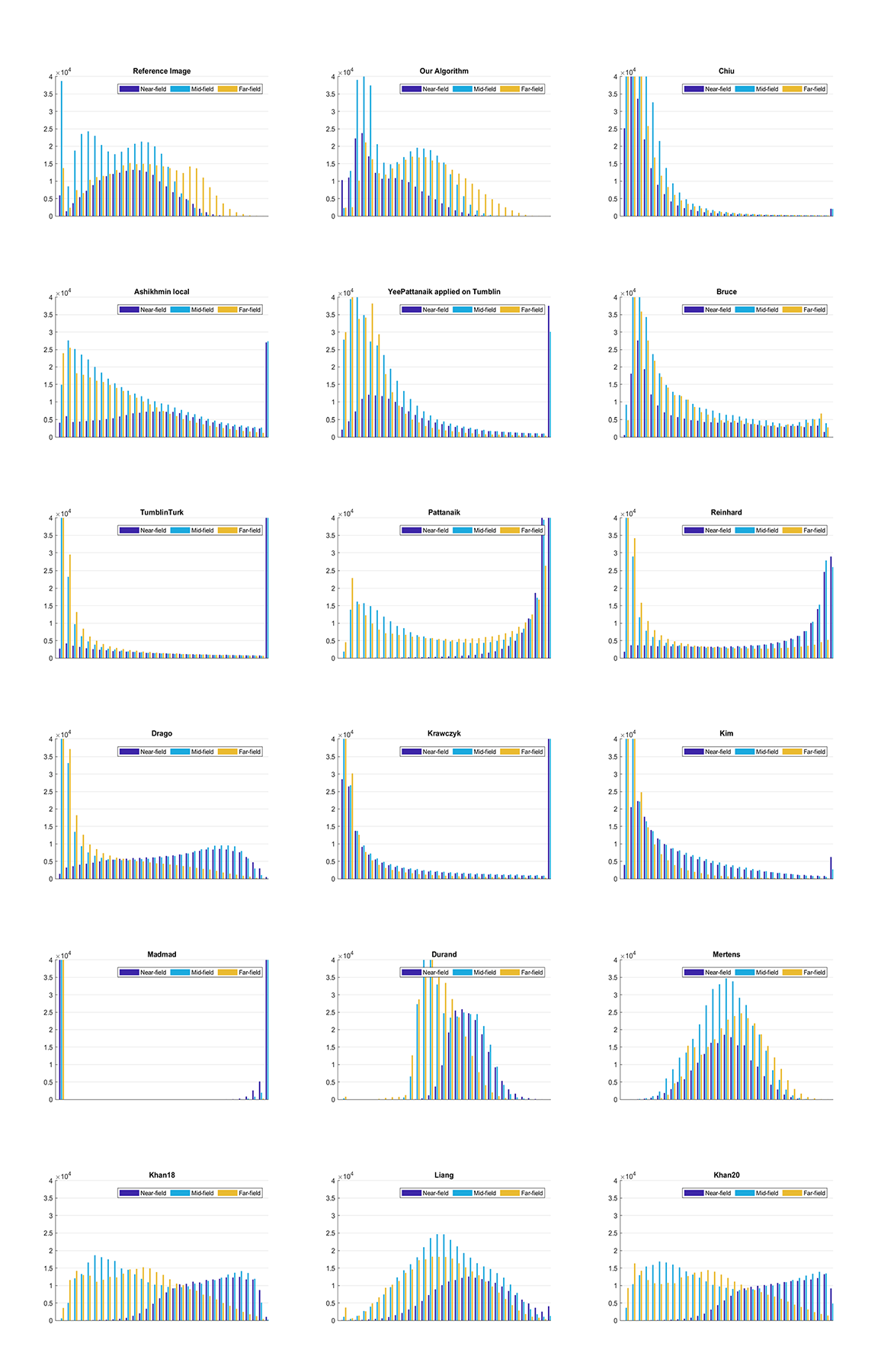

Supplement: S8 Fig — Left to right, top to bottom: image from VOLUSON Expert 22, our proposed method, Artifacts: Chiu, Ashikhmin local, YeePattanaik applied on Tumblin, Bruce; Overexcessive contrast: TumblinTurk, Pattanaik, Reinhard, Drago, Krawczyk, Kim, Madmad; Insufficient contrast: Durand, Mertens, Khan18, Liang, Khan20. (TIFF) [file pone.0340777.s011.tif]

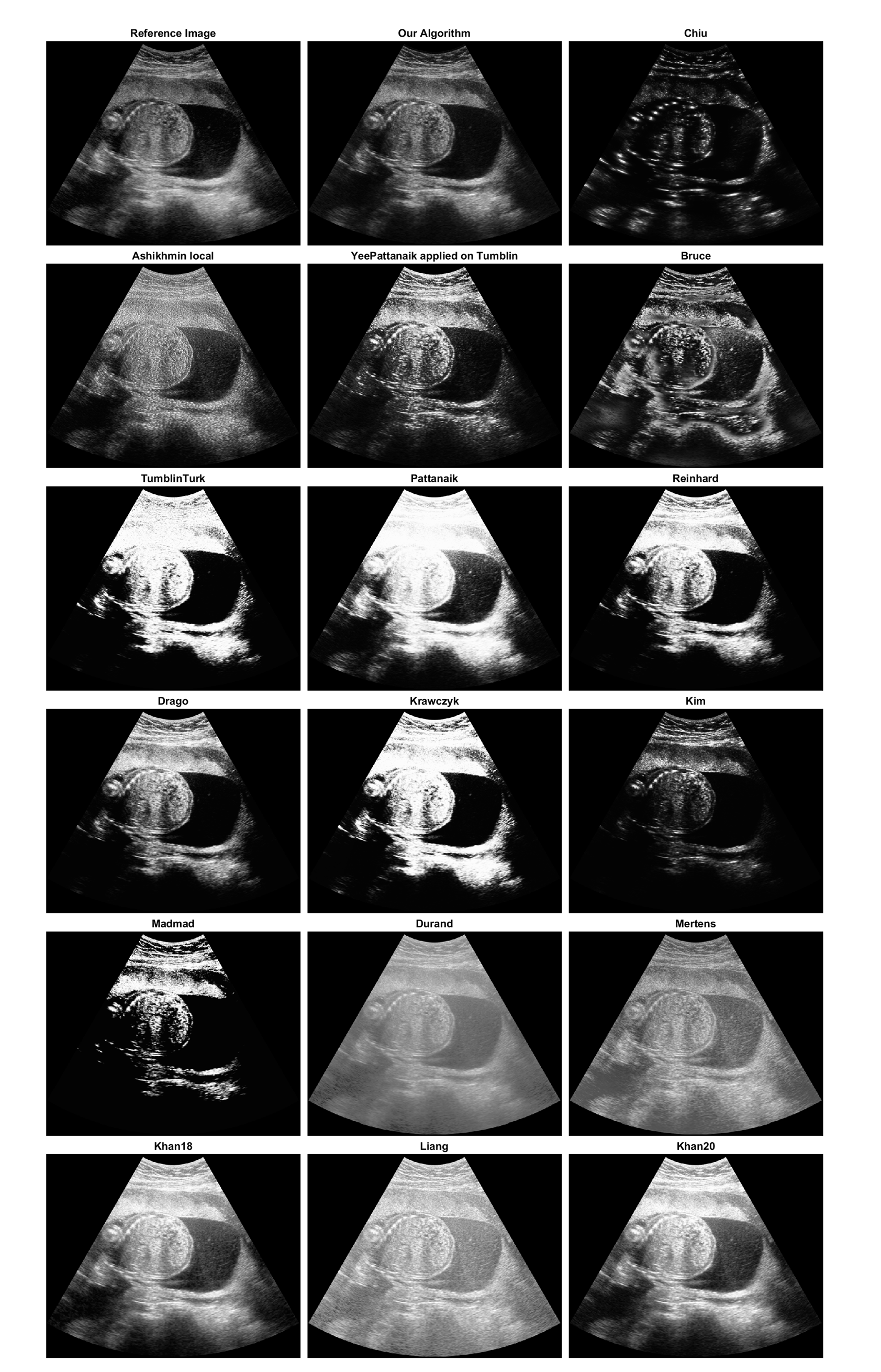

Supplement: S9 Fig — Left to right, top to bottom: image from VOLUSON Expert 22, our proposed method, Artifacts: Chiu, Ashikhmin local, YeePattanaik applied on Tumblin, Bruce; Overexcessive contrast: TumblinTurk, Pattanaik, Reinhard, Drago, Krawczyk, Kim, Madmad; Insufficient contrast: Durand, Mertens, Khan18, Liang, Khan20. (TIFF) [file pone.0340777.s012.tif]

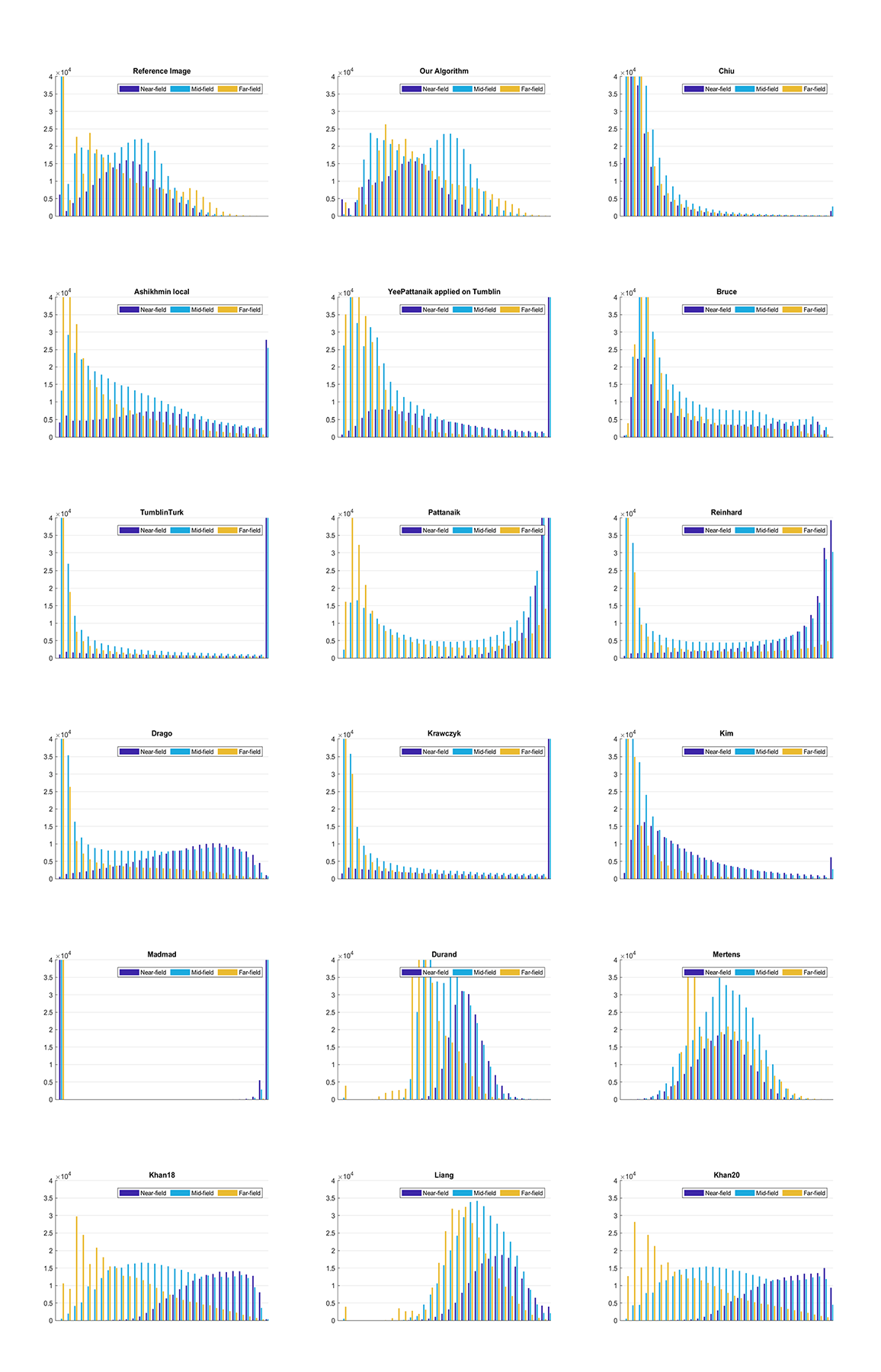

Supplement: S10 Fig — Left to right, top to bottom: image from VOLUSON Expert 22, our proposed method, Artifacts: Chiu, Ashikhmin local, YeePattanaik applied on Tumblin, Bruce; Overexcessive contrast: TumblinTurk, Pattanaik, Reinhard, Drago, Krawczyk, Kim, Madmad; Insufficient contrast: Durand, Mertens, Khan18, Liang, Khan20. (TIFF) [file pone.0340777.s013.tif]

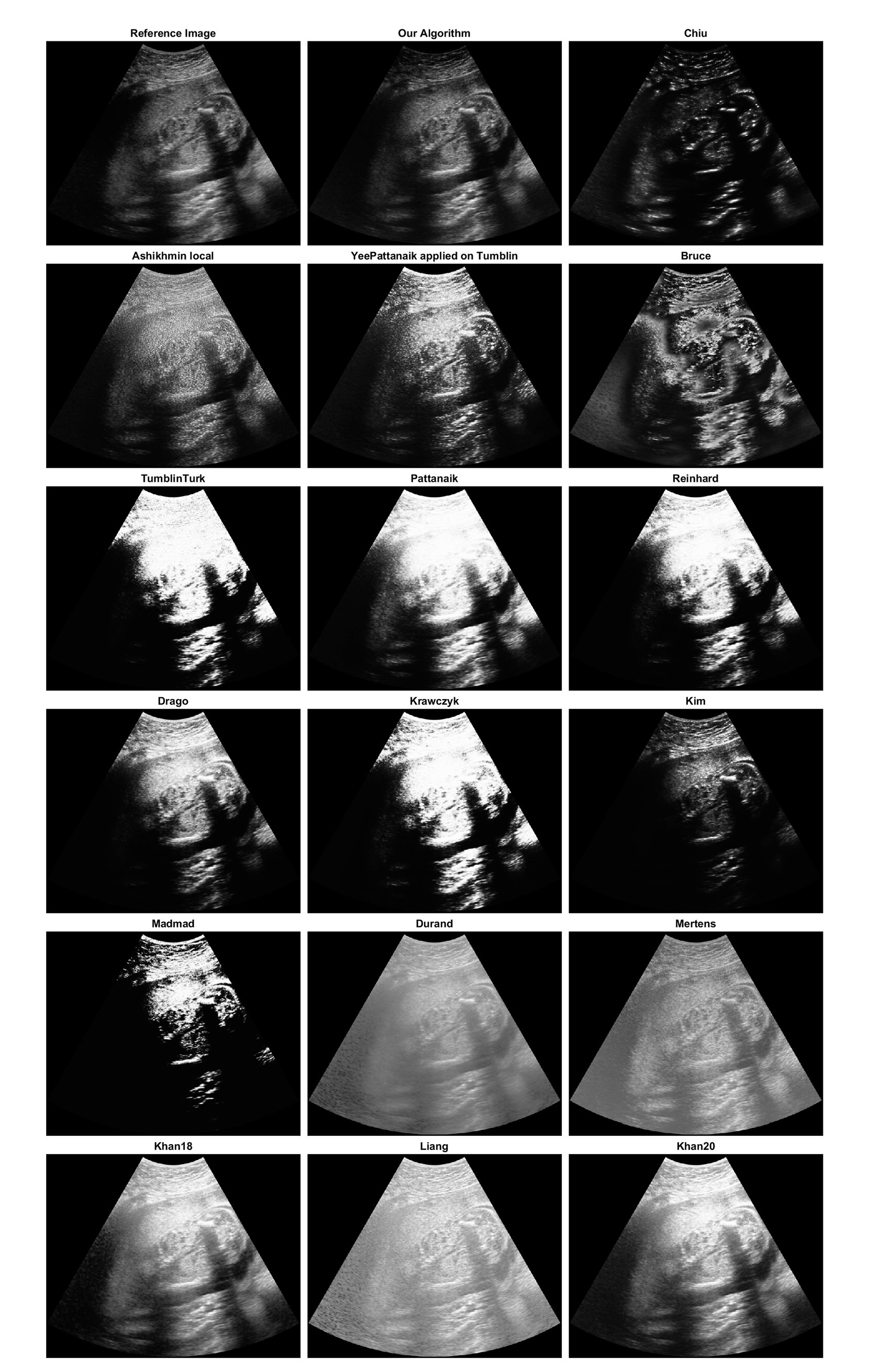

Supplement: S11 Fig — Left to right, top to bottom: image from VOLUSON Expert 22, our proposed method, Artifacts: Chiu, Ashikhmin local, YeePattanaik applied on Tumblin, Bruce; Overexcessive contrast: TumblinTurk, Pattanaik, Reinhard, Drago, Krawczyk, Kim, Madmad; Insufficient contrast: Durand, Mertens, Khan18, Liang, Khan20. (TIFF) [file pone.0340777.s014.tif]

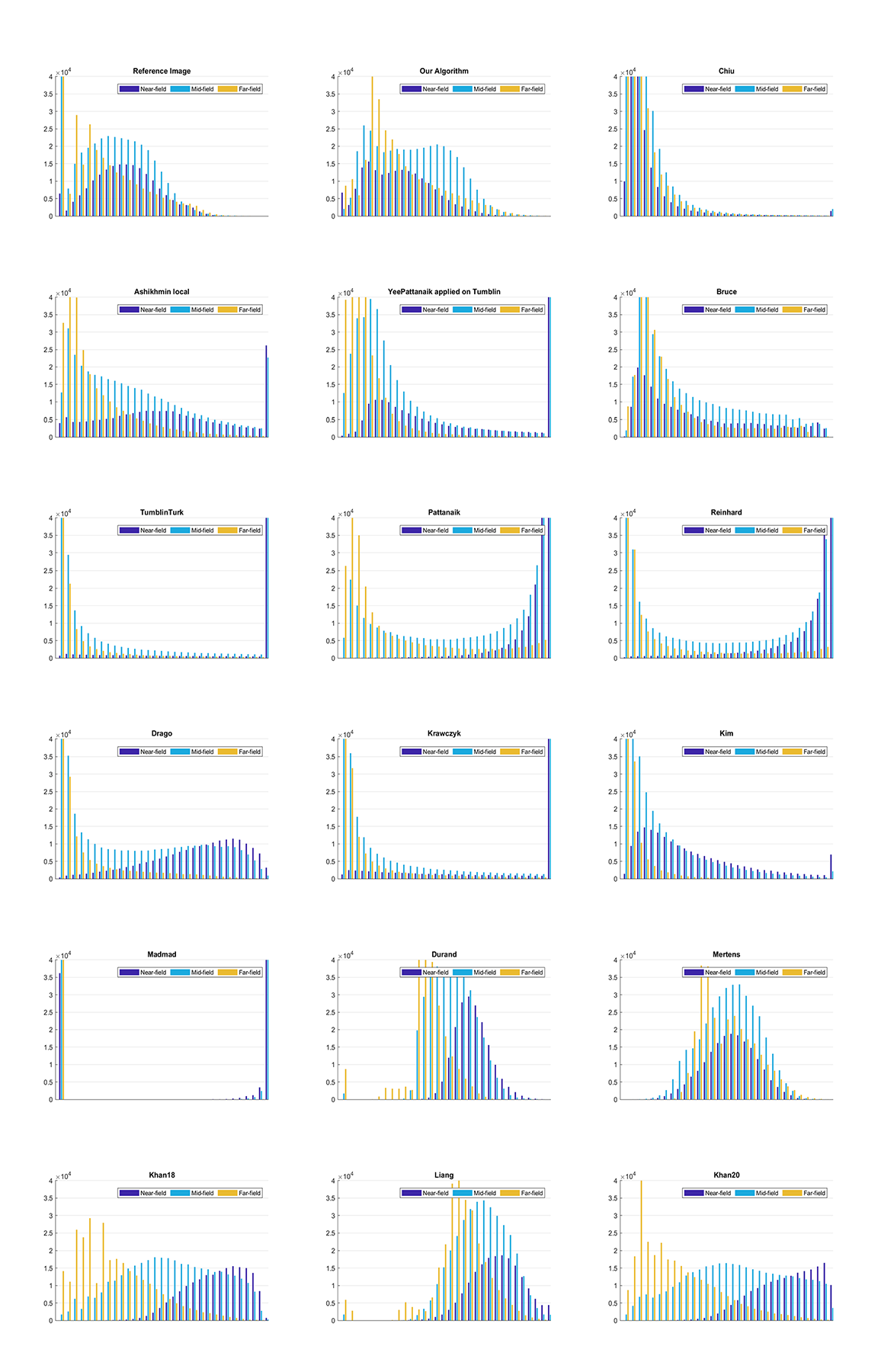

Supplement: S12 Fig — Left to right, top to bottom: image from VOLUSON Expert 22, our proposed method, Artifacts: Chiu, Ashikhmin local, YeePattanaik applied on Tumblin, Bruce; Overexcessive contrast: TumblinTurk, Pattanaik, Reinhard, Drago, Krawczyk, Kim, Madmad; Insufficient contrast: Durand, Mertens, Khan18, Liang, Khan20. (TIFF) [file pone.0340777.s015.tif]

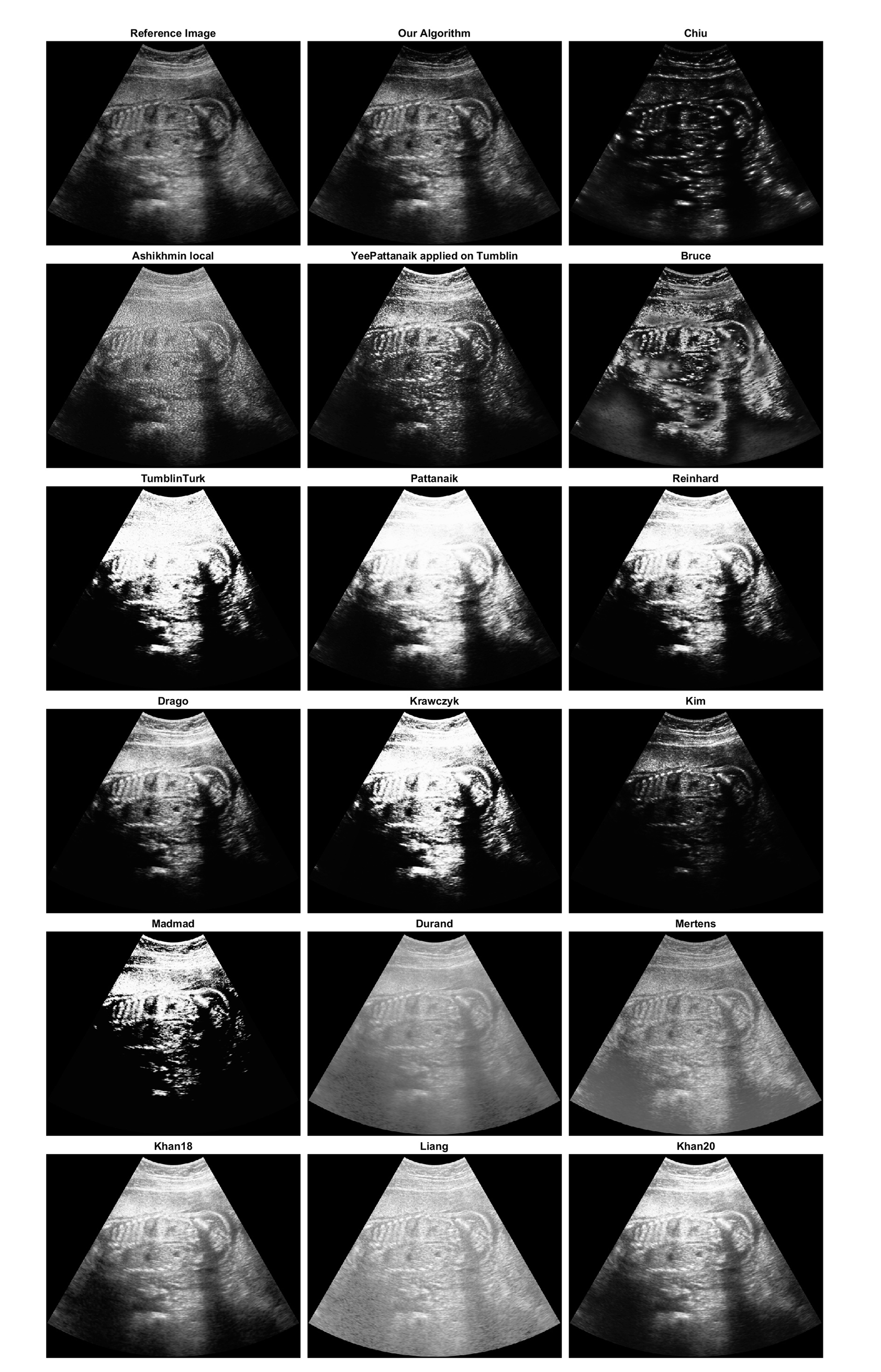

Supplement: S13 Fig — Left to right, top to bottom: image from VOLUSON Expert 22, our proposed method, Artifacts: Chiu, Ashikhmin local, YeePattanaik applied on Tumblin, Bruce; Overexcessive contrast: TumblinTurk, Pattanaik, Reinhard, Drago, Krawczyk, Kim, Madmad; Insufficient contrast: Durand, Mertens, Khan18, Liang, Khan20. (TIFF) [file pone.0340777.s016.tif]

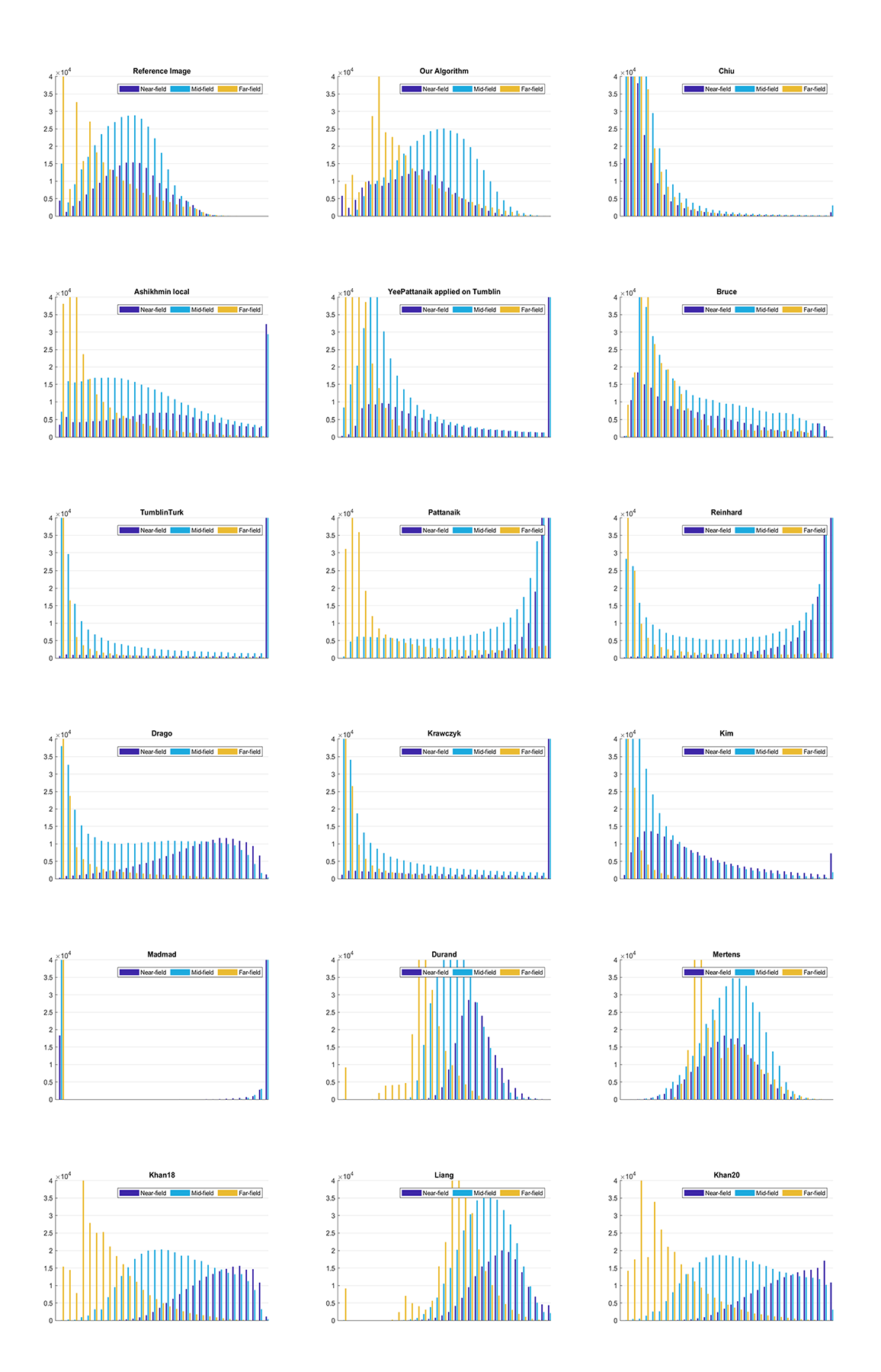

Supplement: S14 Fig — Left to right, top to bottom: image from VOLUSON Expert 22, our proposed method, Artifacts: Chiu, Ashikhmin local, YeePattanaik applied on Tumblin, Bruce; Overexcessive contrast: TumblinTurk, Pattanaik, Reinhard, Drago, Krawczyk, Kim, Madmad; Insufficient contrast: Durand, Mertens, Khan18, Liang, Khan20. (TIFF) [file pone.0340777.s017.tif]

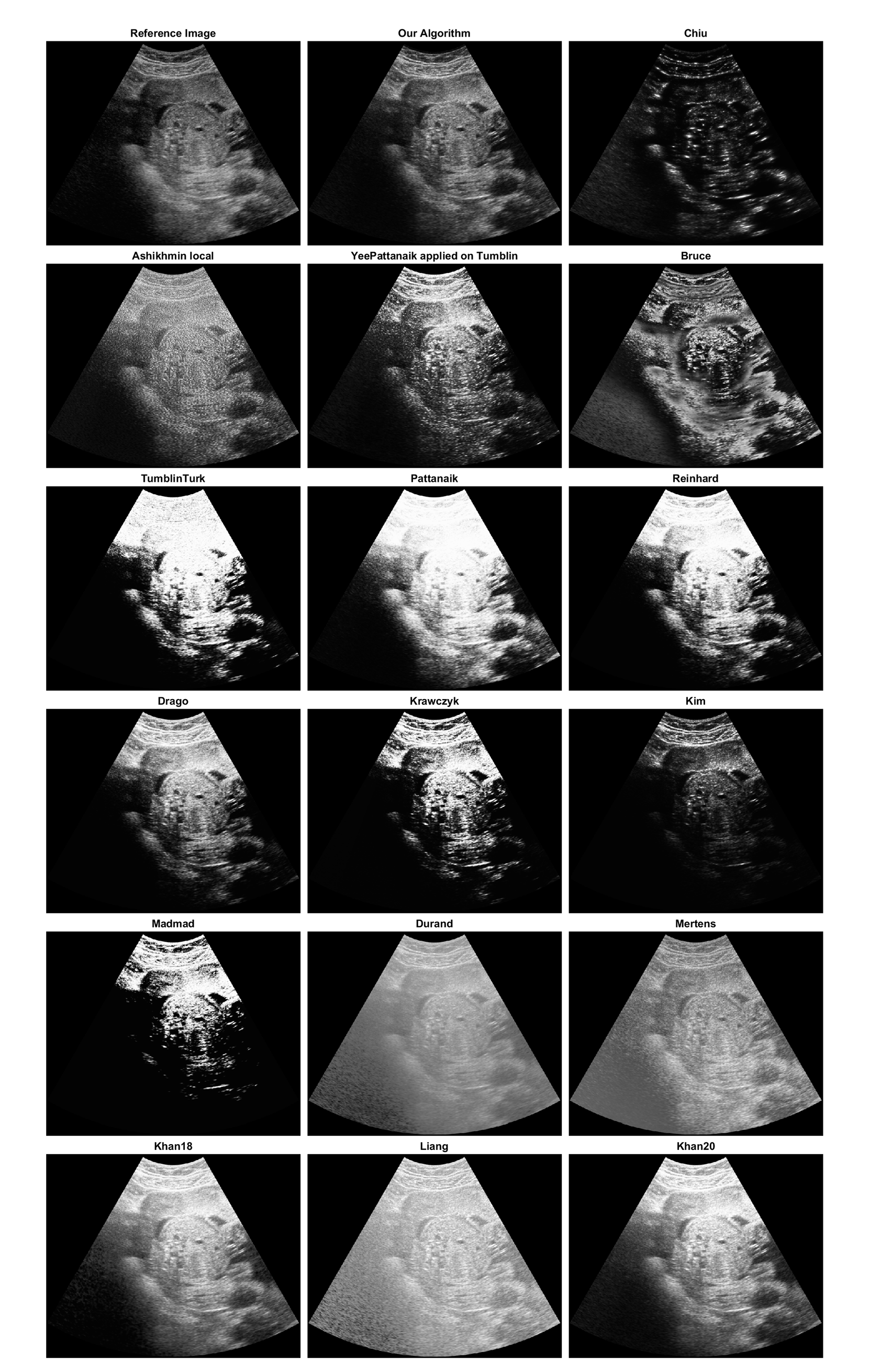

Supplement: S15 Fig — Left to right, top to bottom: image from VOLUSON Expert 22, our proposed method, Artifacts: Chiu, Ashikhmin local, YeePattanaik applied on Tumblin, Bruce; Overexcessive contrast: TumblinTurk, Pattanaik, Reinhard, Drago, Krawczyk, Kim, Madmad; Insufficient contrast: Durand, Mertens, Khan18, Liang, Khan20. (TIFF) [file pone.0340777.s018.tif]

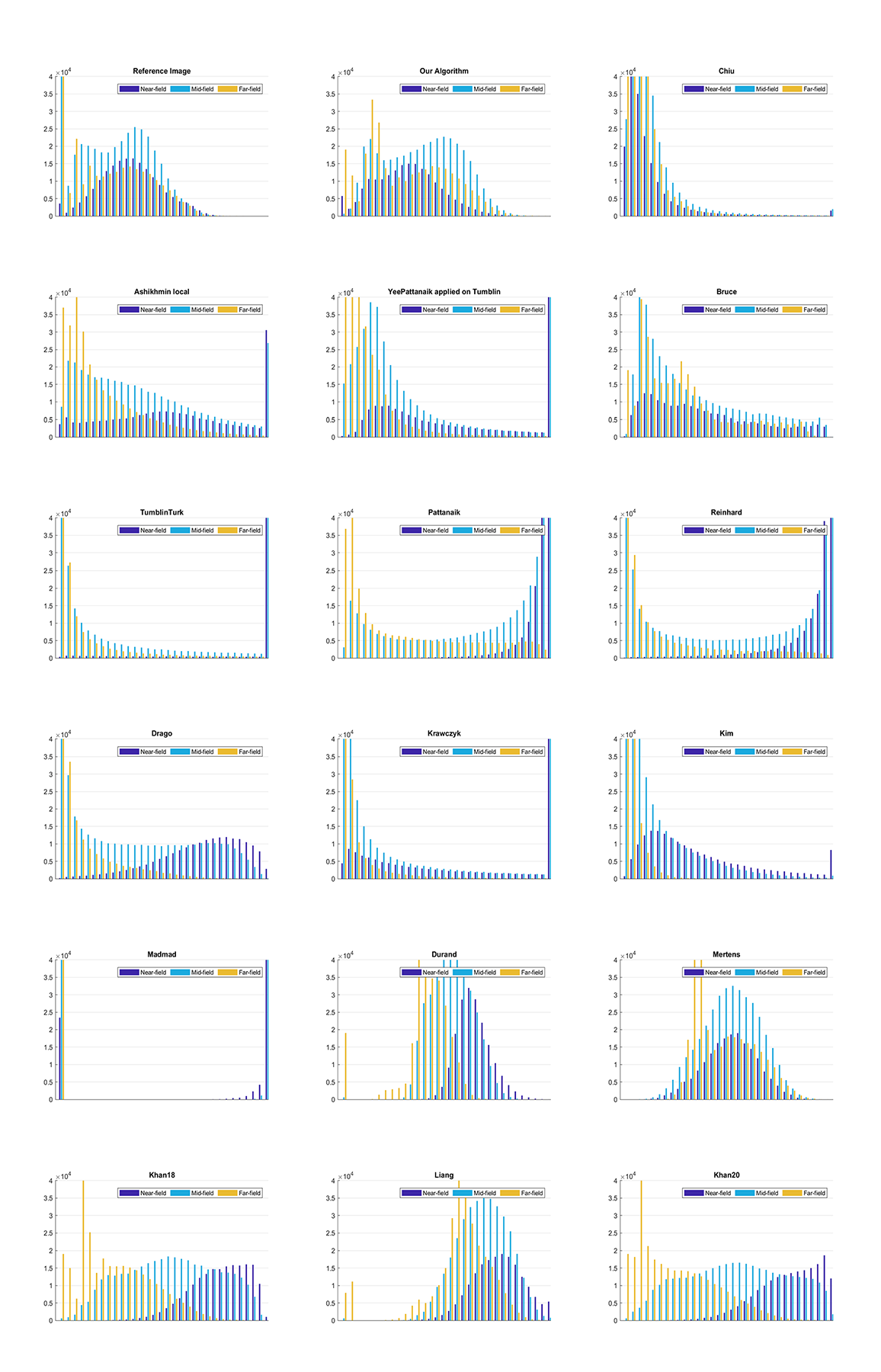

Supplement: S16 Fig — Left to right, top to bottom: image from VOLUSON Expert 22, our proposed method, Artifacts: Chiu, Ashikhmin local, YeePattanaik applied on Tumblin, Bruce; Overexcessive contrast: TumblinTurk, Pattanaik, Reinhard, Drago, Krawczyk, Kim, Madmad; Insufficient contrast: Durand, Mertens, Khan18, Liang, Khan20. (TIFF) [file pone.0340777.s019.tif]

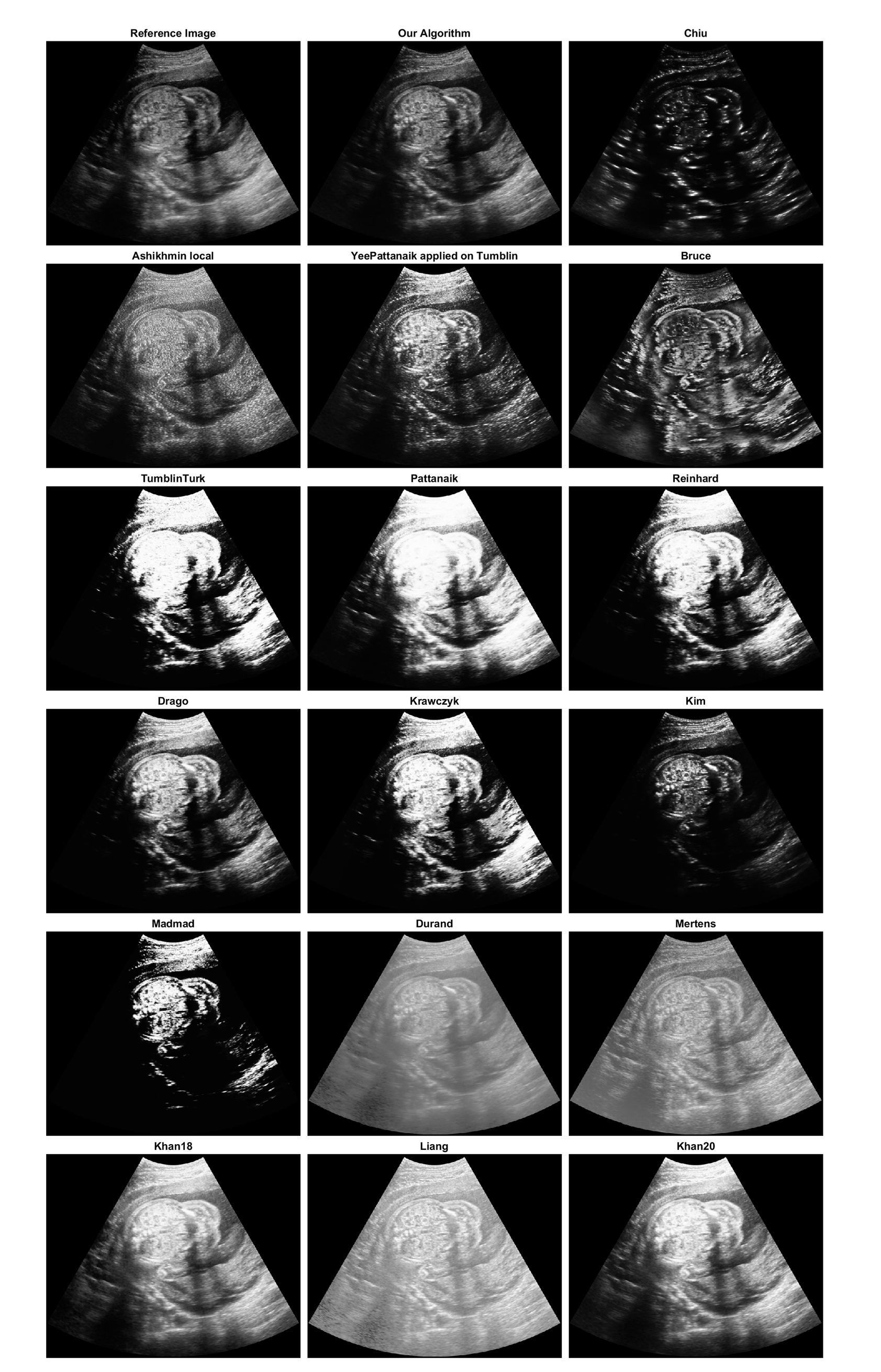

Supplement: S17 Fig — Left to right, top to bottom: image from VOLUSON Expert 22, our proposed method, Artifacts: Chiu, Ashikhmin local, YeePattanaik applied on Tumblin, Bruce; Overexcessive contrast: TumblinTurk, Pattanaik, Reinhard, Drago, Krawczyk, Kim, Madmad; Insufficient contrast: Durand, Mertens, Khan18, Liang, Khan20. (TIFF) [file pone.0340777.s020.tif]

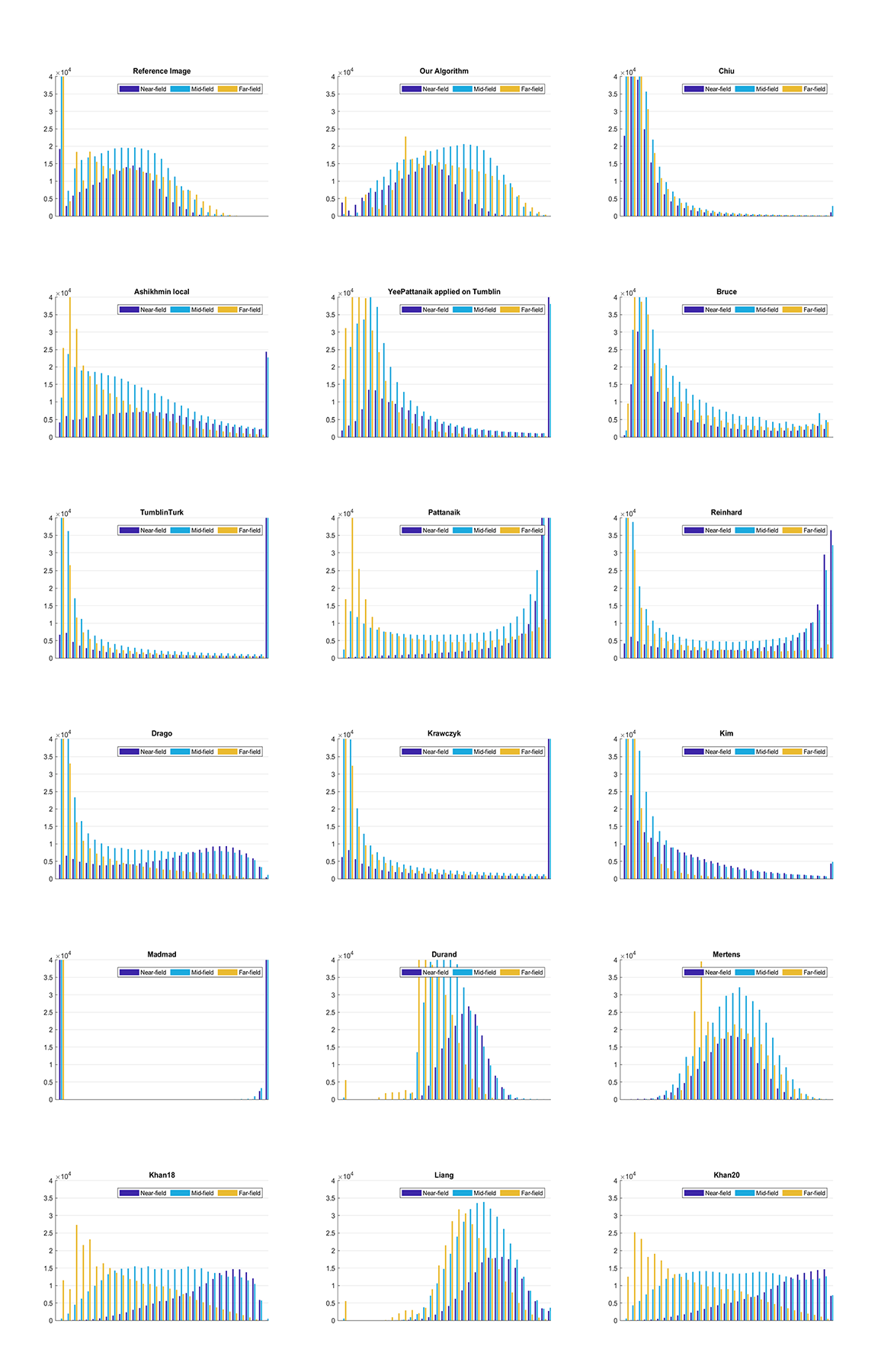

Supplement: S18 Fig — Left to right, top to bottom: image from VOLUSON Expert 22, our proposed method, Artifacts: Chiu, Ashikhmin local, YeePattanaik applied on Tumblin, Bruce; Overexcessive contrast: TumblinTurk, Pattanaik, Reinhard, Drago, Krawczyk, Kim, Madmad; Insufficient contrast: Durand, Mertens, Khan18, Liang, Khan20. (TIFF) [file pone.0340777.s021.tif]

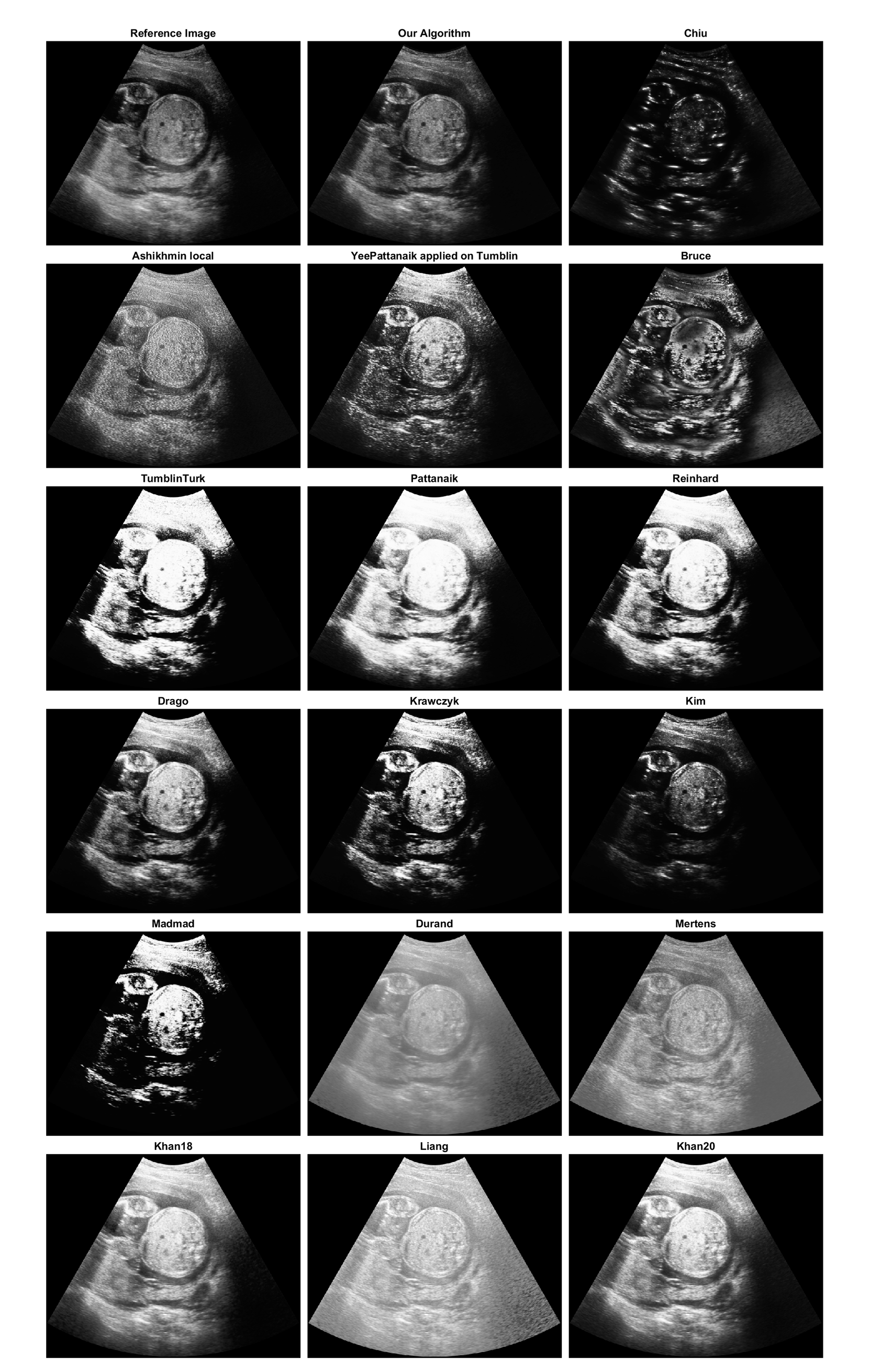

Supplement: S19 Fig — Left to right, top to bottom: image from VOLUSON Expert 22, our proposed method, Artifacts: Chiu, Ashikhmin local, YeePattanaik applied on Tumblin, Bruce; Overexcessive contrast: TumblinTurk, Pattanaik, Reinhard, Drago, Krawczyk, Kim, Madmad; Insufficient contrast: Durand, Mertens, Khan18, Liang, Khan20. (TIFF) [file pone.0340777.s022.tif]

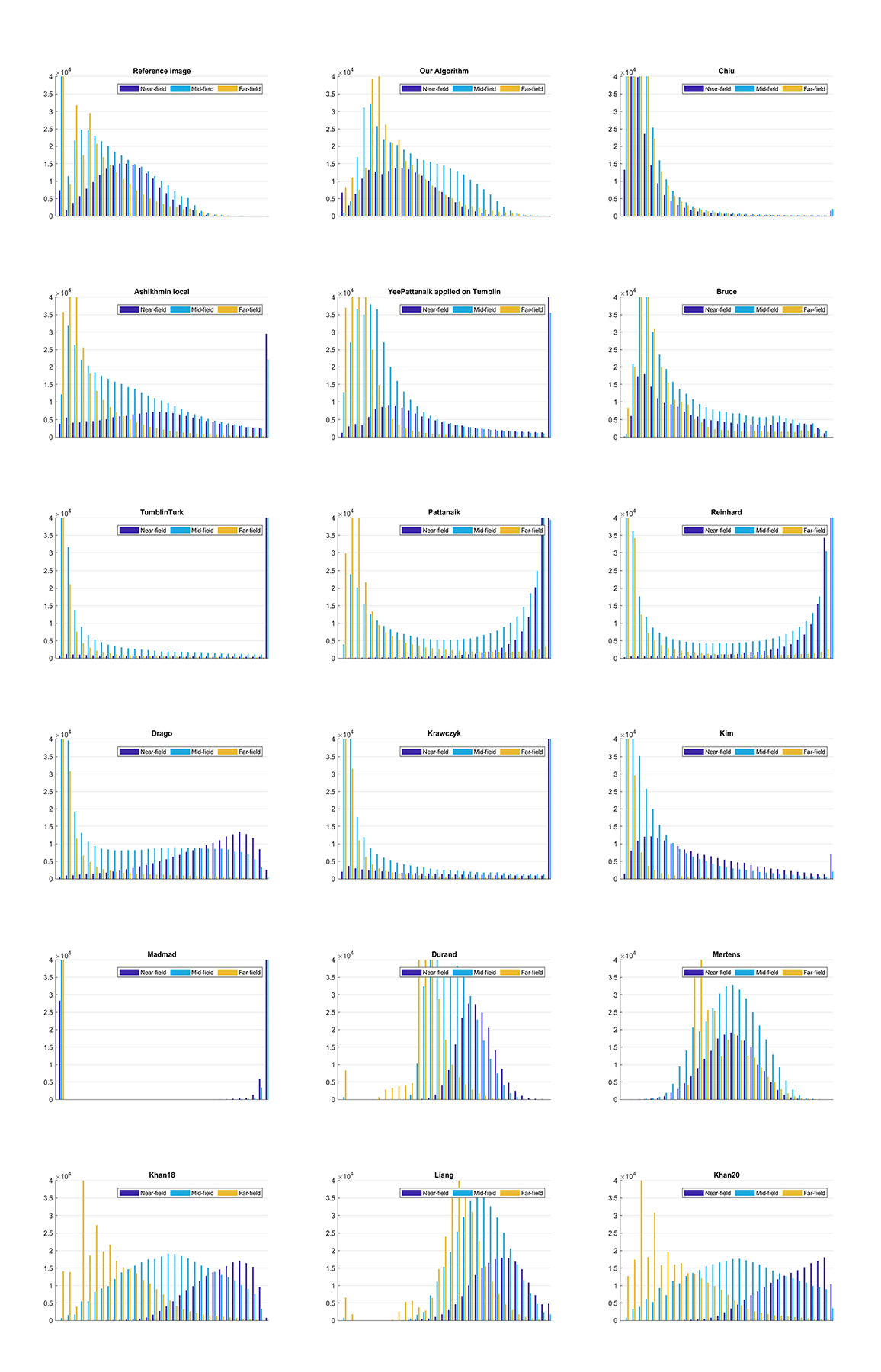

Supplement: S20 Fig — Left to right, top to bottom: image from VOLUSON Expert 22, our proposed method, Artifacts: Chiu, Ashikhmin local, YeePattanaik applied on Tumblin, Bruce; Overexcessive contrast: TumblinTurk, Pattanaik, Reinhard, Drago, Krawczyk, Kim, Madmad; Insufficient contrast: Durand, Mertens, Khan18, Liang, Khan20. (TIFF) [file pone.0340777.s023.tif]

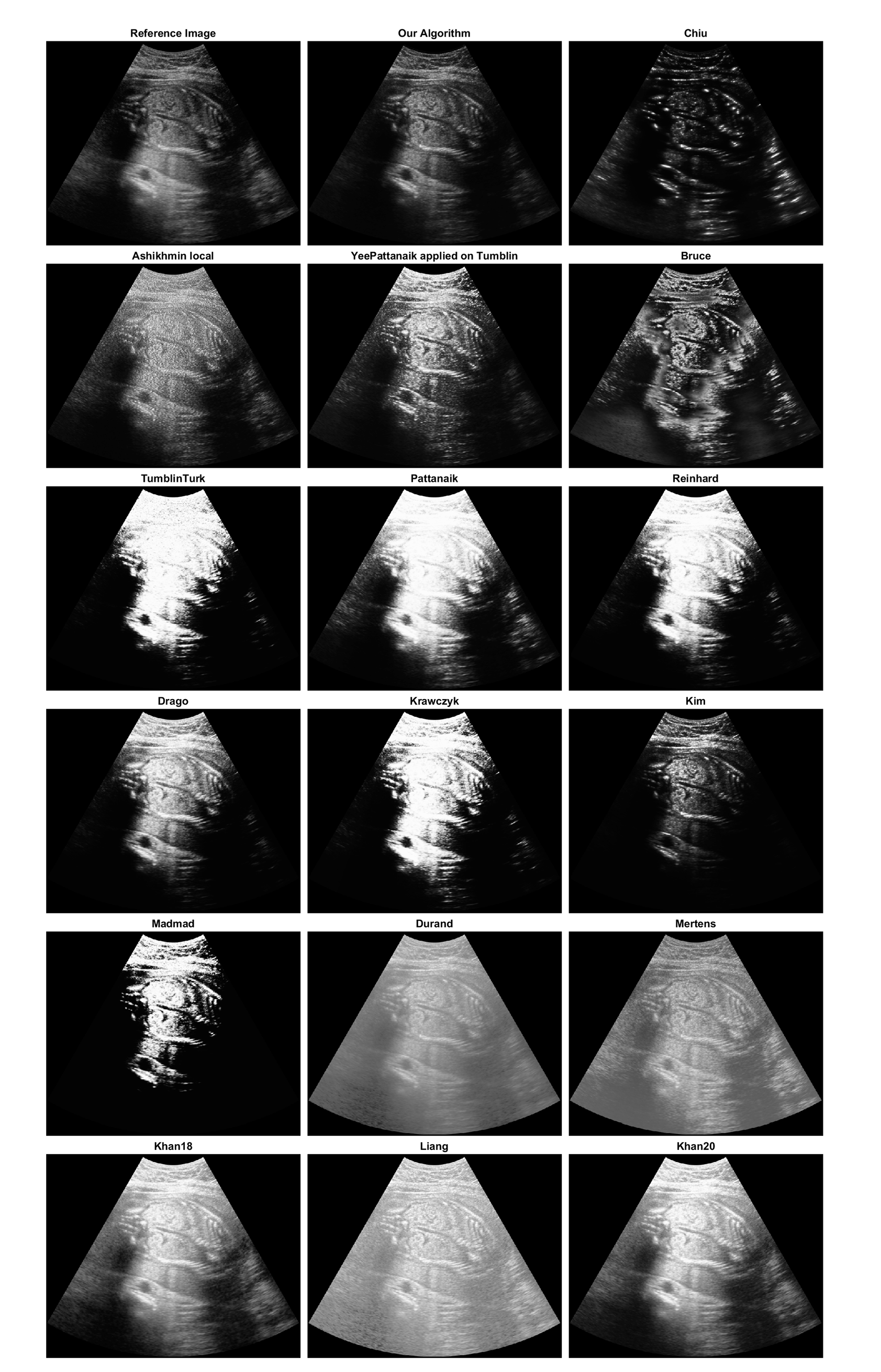

Supplement: S21 Fig — Left to right, top to bottom: image from VOLUSON Expert 22, our proposed method, Artifacts: Chiu, Ashikhmin local, YeePattanaik applied on Tumblin, Bruce; Overexcessive contrast: TumblinTurk, Pattanaik, Reinhard, Drago, Krawczyk, Kim, Madmad; Insufficient contrast: Durand, Mertens, Khan18, Liang, Khan20. (TIFF) [file pone.0340777.s024.tif]

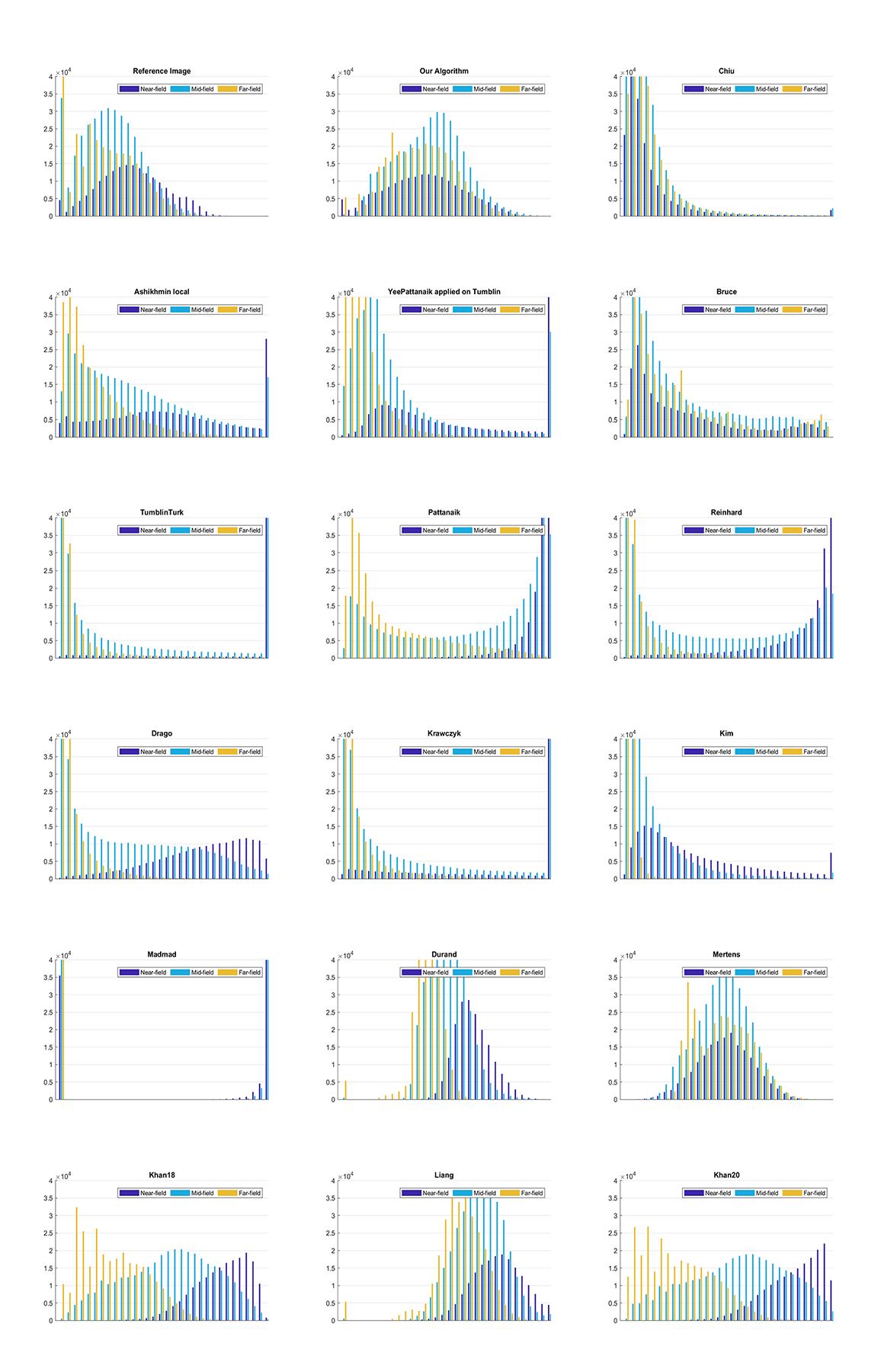

Supplement: S22 Fig — Left to right, top to bottom: image from VOLUSON Expert 22, our proposed method, Artifacts: Chiu, Ashikhmin local, YeePattanaik applied on Tumblin, Bruce; Overexcessive contrast: TumblinTurk, Pattanaik, Reinhard, Drago, Krawczyk, Kim, Madmad; Insufficient contrast: Durand, Mertens, Khan18, Liang, Khan20. (TIFF) [file pone.0340777.s025.tif]

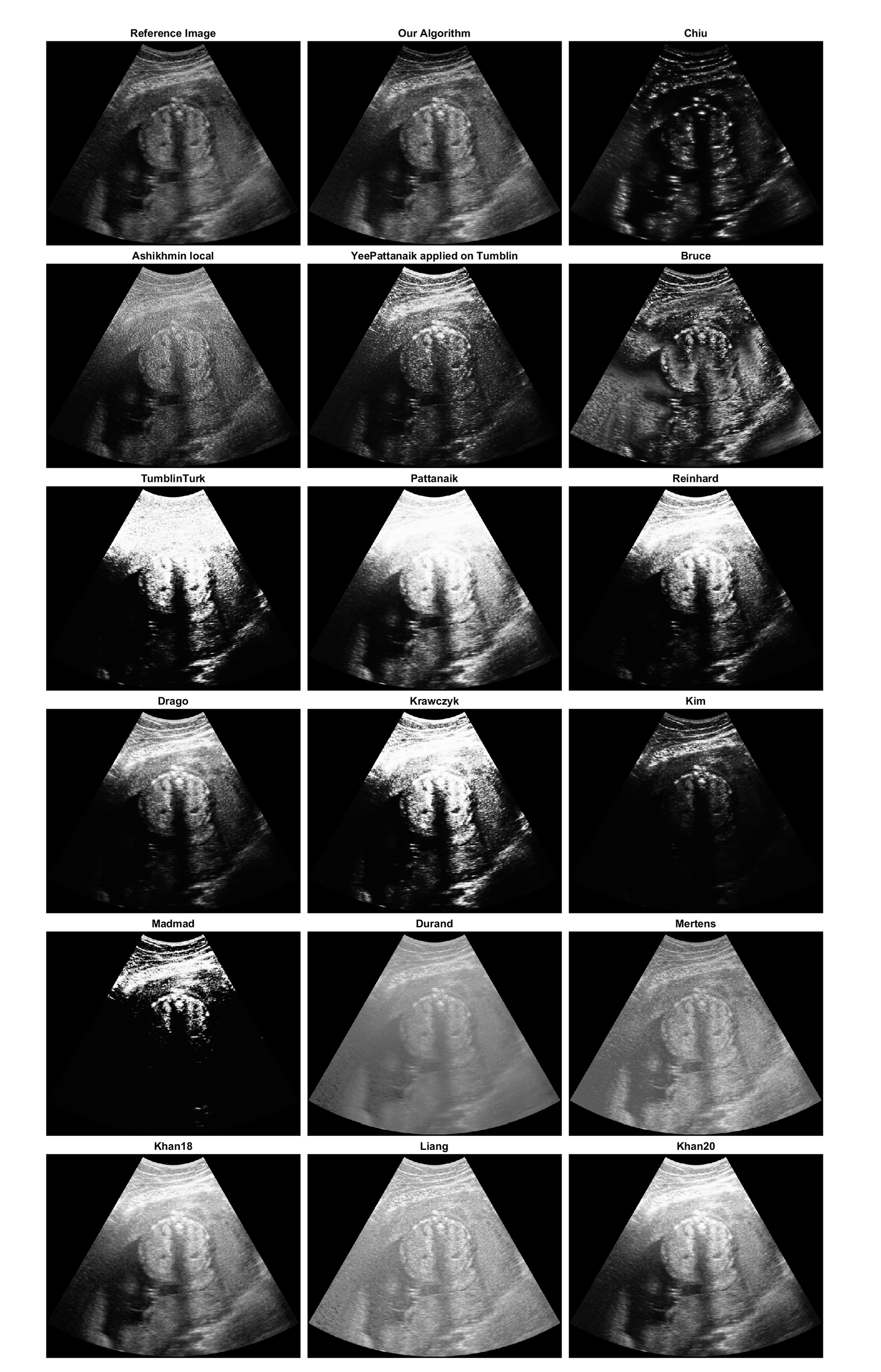

Supplement: S23 Fig — Left to right, top to bottom: image from VOLUSON Expert 22, our proposed method, Artifacts: Chiu, Ashikhmin local, YeePattanaik applied on Tumblin, Bruce; Overexcessive contrast: TumblinTurk, Pattanaik, Reinhard, Drago, Krawczyk, Kim, Madmad; Insufficient contrast: Durand, Mertens, Khan18, Liang, Khan20. (TIFF) [file pone.0340777.s026.tif]

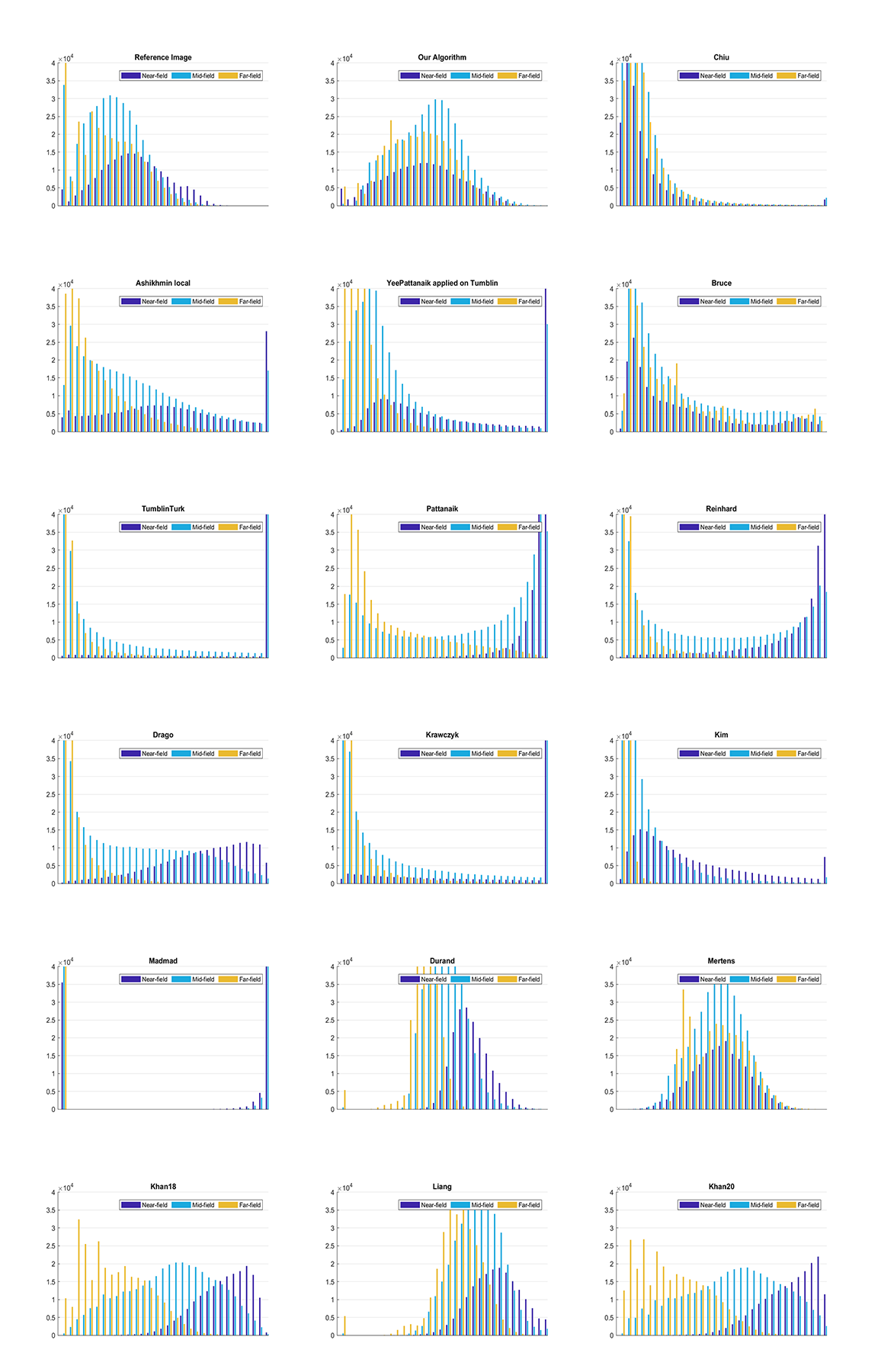

Supplement: S24 Fig — Left to right, top to bottom: image from VOLUSON Expert 22, our proposed method, Artifacts: Chiu, Ashikhmin local, YeePattanaik applied on Tumblin, Bruce; Overexcessive contrast: TumblinTurk, Pattanaik, Reinhard, Drago, Krawczyk, Kim, Madmad; Insufficient contrast: Durand, Mertens, Khan18, Liang, Khan20. (TIFF) [file pone.0340777.s027.tif]

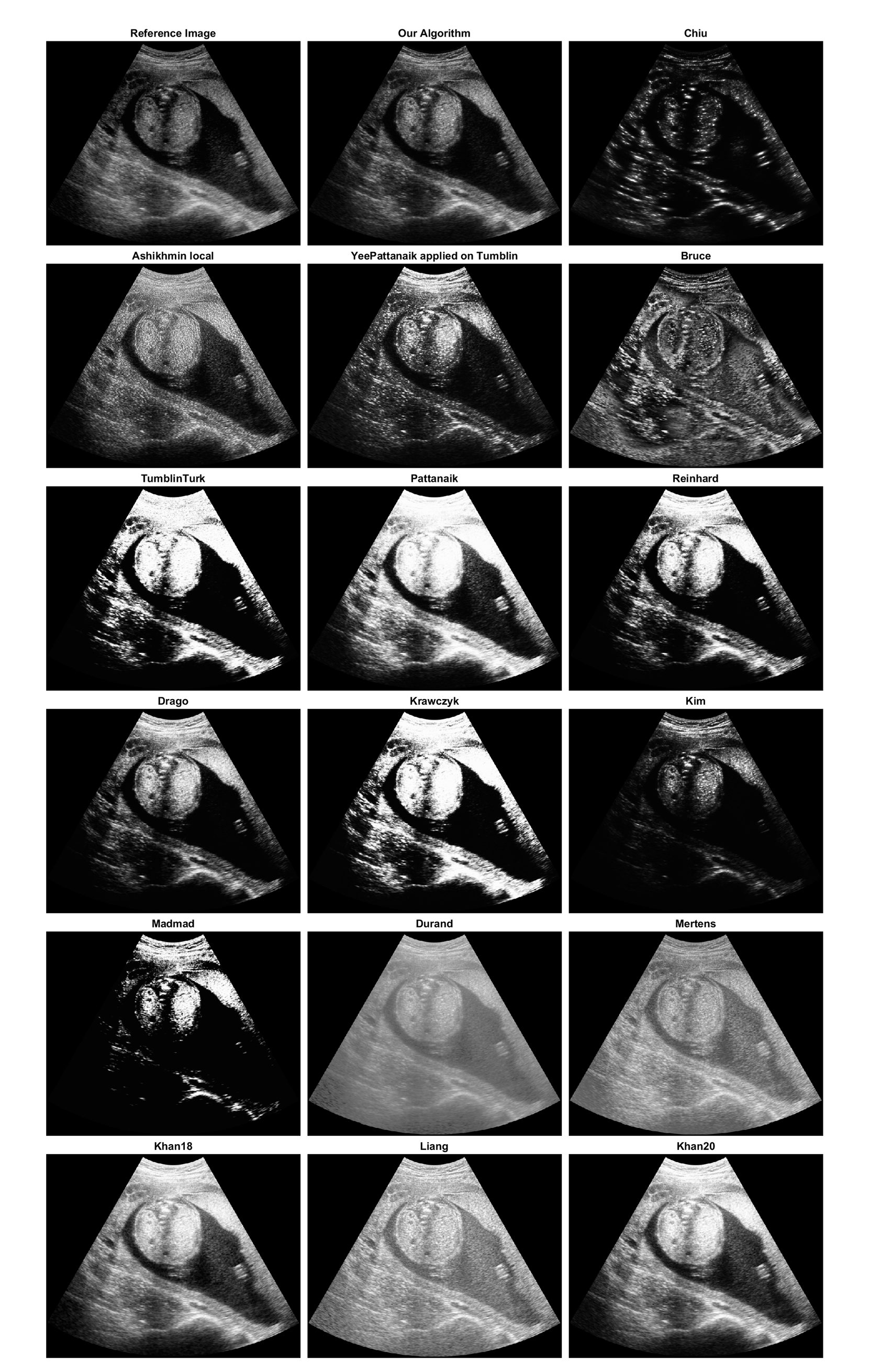

Supplement: S25 Fig — Left to right, top to bottom: image from VOLUSON Expert 22, our proposed method, Artifacts: Chiu, Ashikhmin local, YeePattanaik applied on Tumblin, Bruce; Overexcessive contrast: TumblinTurk, Pattanaik, Reinhard, Drago, Krawczyk, Kim, Madmad; Insufficient contrast: Durand, Mertens, Khan18, Liang, Khan20. (TIFF) [file pone.0340777.s028.tif]

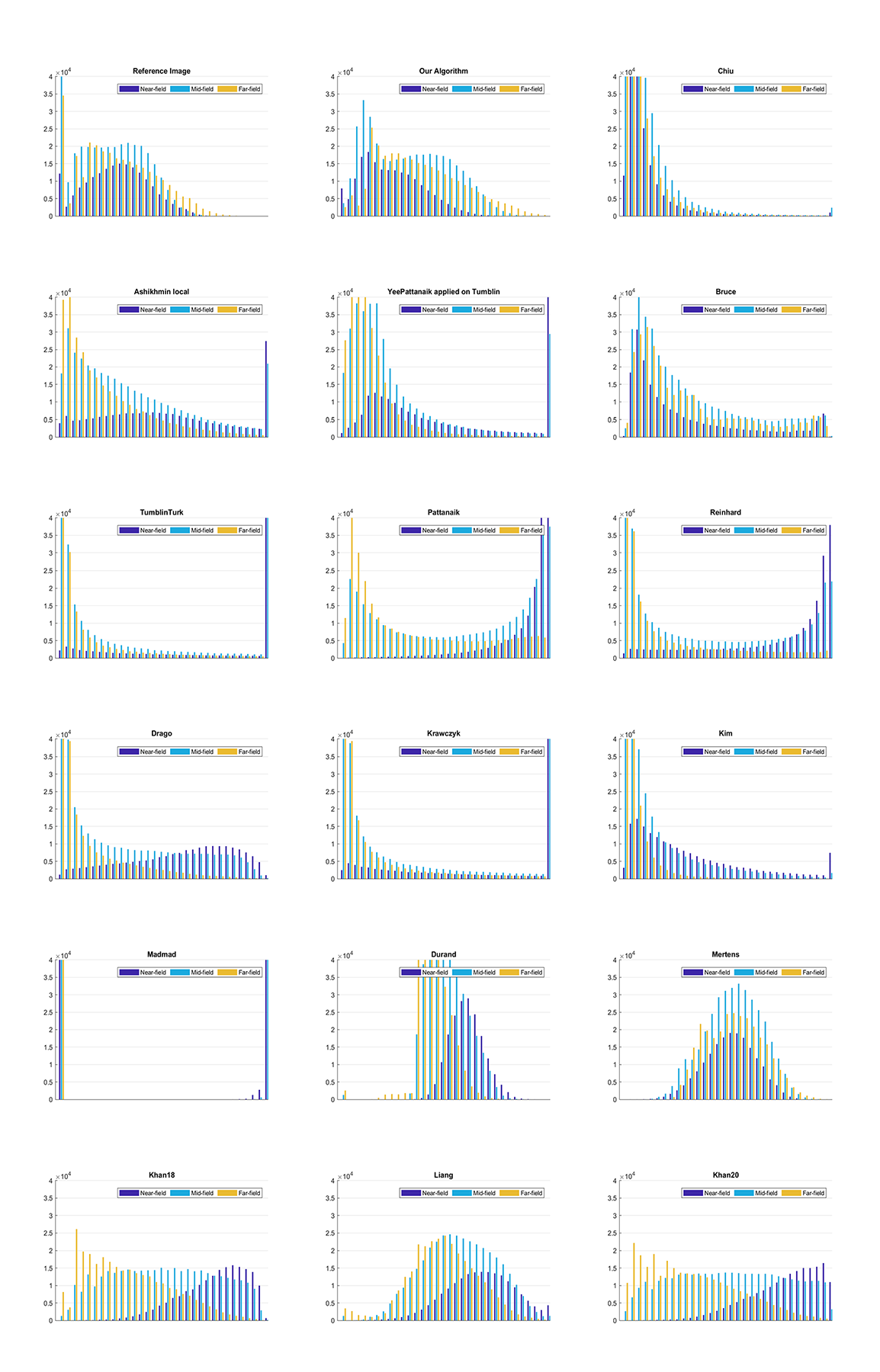

Supplement: S26 Fig — Left to right, top to bottom: image from VOLUSON Expert 22, our proposed method, Artifacts: Chiu, Ashikhmin local, YeePattanaik applied on Tumblin, Bruce; Overexcessive contrast: TumblinTurk, Pattanaik, Reinhard, Drago, Krawczyk, Kim, Madmad; Insufficient contrast: Durand, Mertens, Khan18, Liang, Khan20. (TIFF) [file pone.0340777.s029.tif]

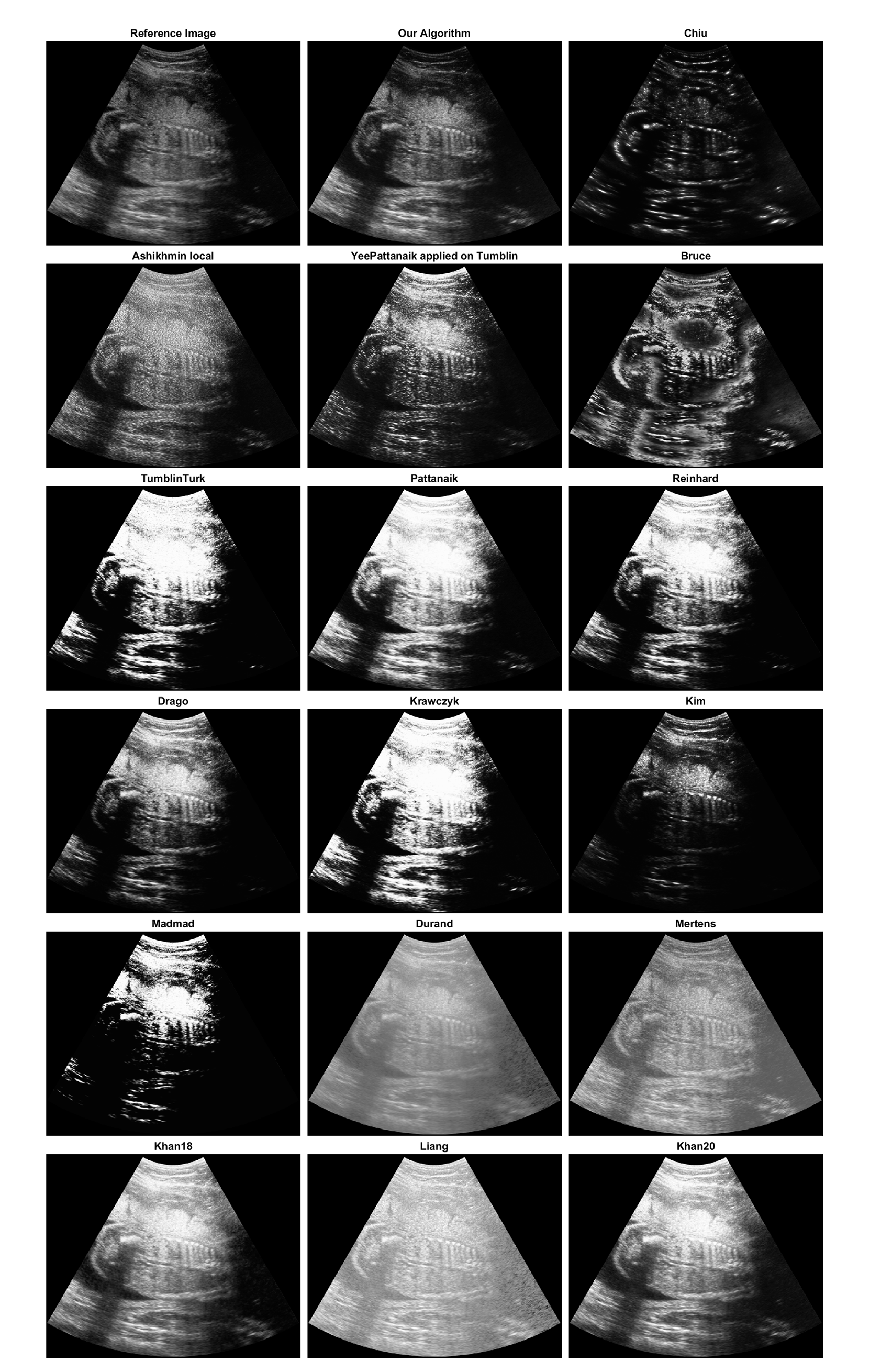

Supplement: S27 Fig — Left to right, top to bottom: image from VOLUSON Expert 22, our proposed method, Artifacts: Chiu, Ashikhmin local, YeePattanaik applied on Tumblin, Bruce; Overexcessive contrast: TumblinTurk, Pattanaik, Reinhard, Drago, Krawczyk, Kim, Madmad; Insufficient contrast: Durand, Mertens, Khan18, Liang, Khan20. (TIFF) [file pone.0340777.s030.tif]

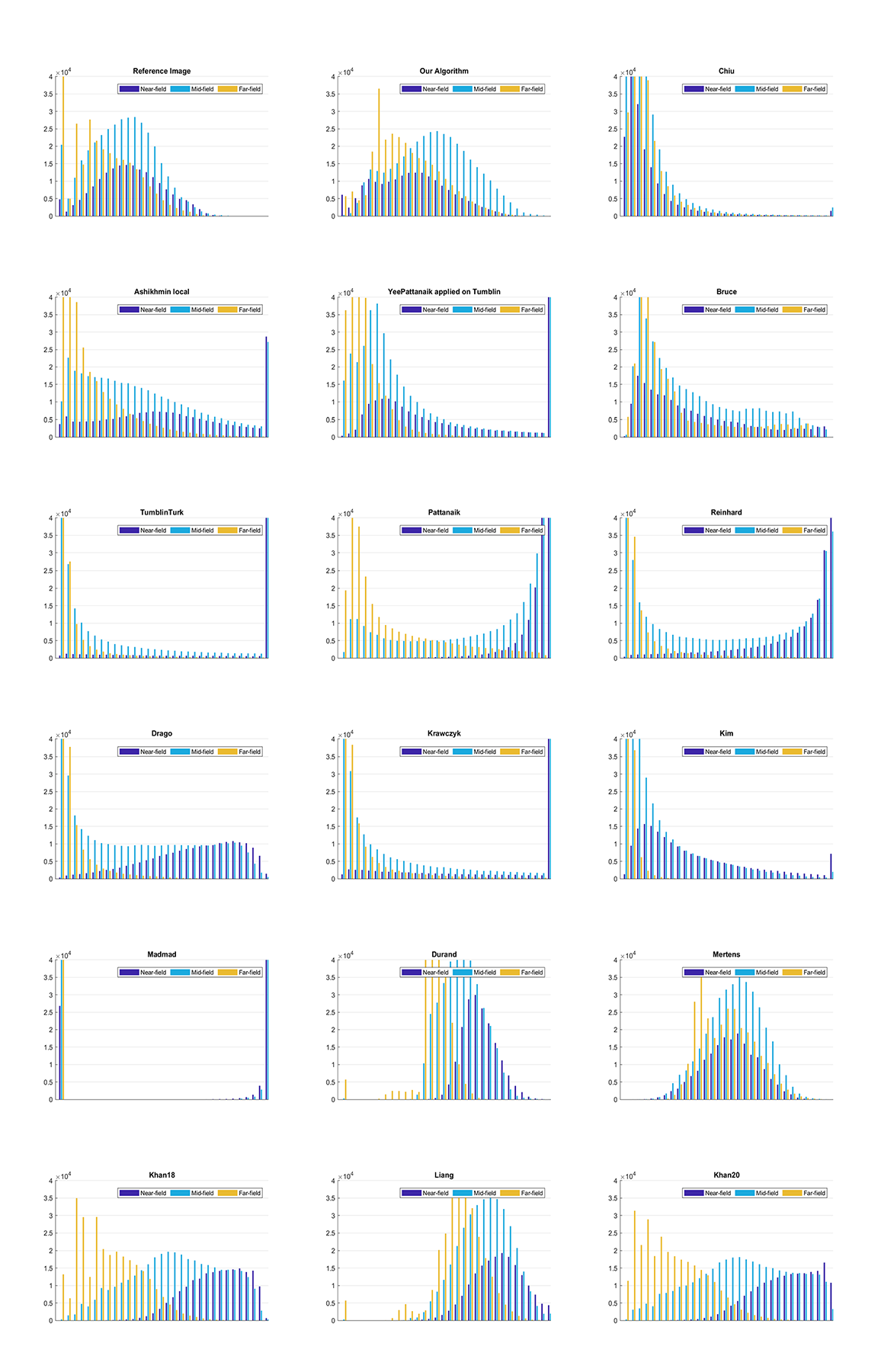

Supplement: S28 Fig — Left to right, top to bottom: image from VOLUSON Expert 22, our proposed method, Artifacts: Chiu, Ashikhmin local, YeePattanaik applied on Tumblin, Bruce; Overexcessive contrast: TumblinTurk, Pattanaik, Reinhard, Drago, Krawczyk, Kim, Madmad; Insufficient contrast: Durand, Mertens, Khan18, Liang, Khan20. (TIFF) [file pone.0340777.s031.tif]

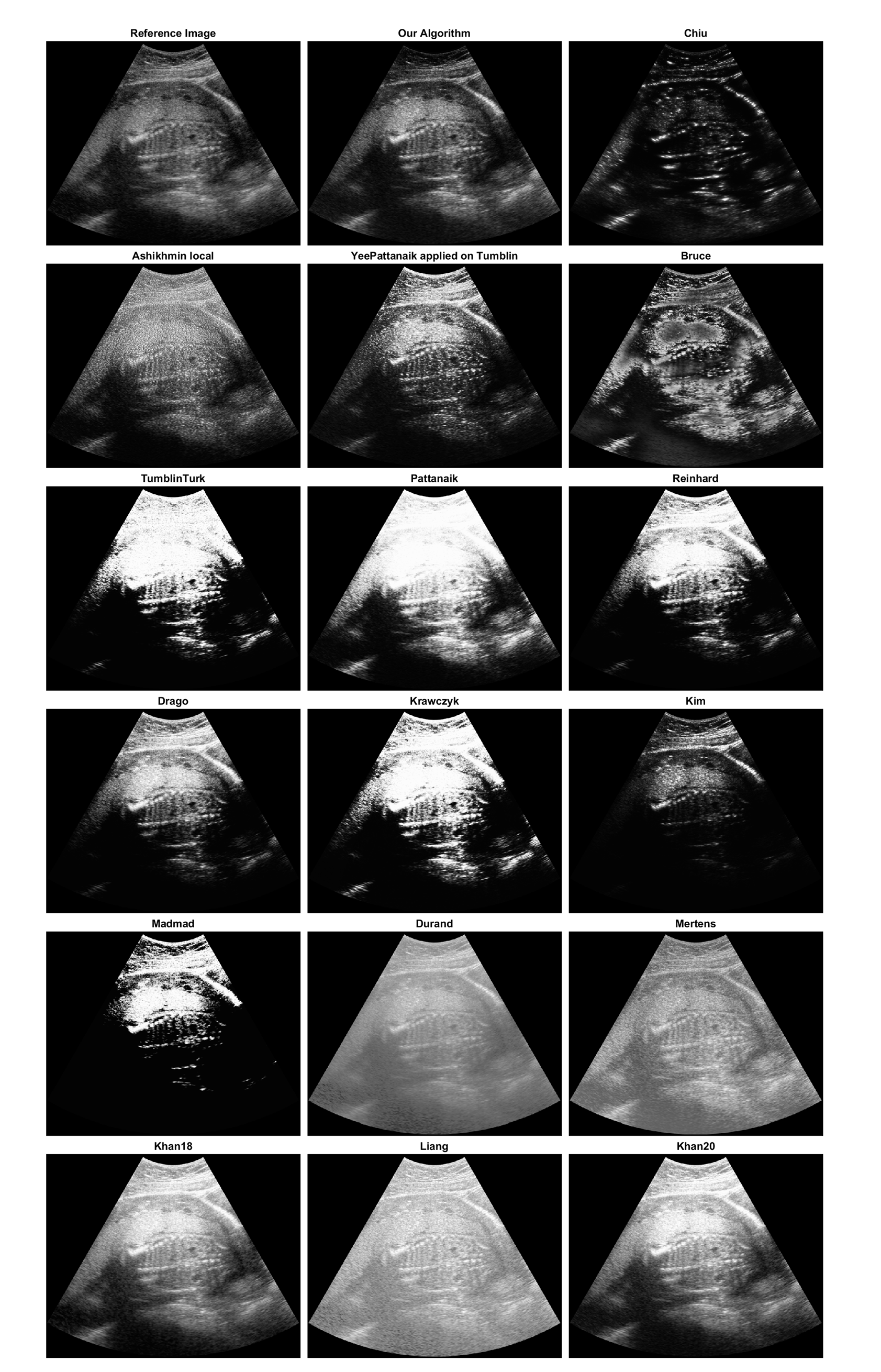

Supplement: S29 Fig — Left to right, top to bottom: image from VOLUSON Expert 22, our proposed method, Artifacts: Chiu, Ashikhmin local, YeePattanaik applied on Tumblin, Bruce; Overexcessive contrast: TumblinTurk, Pattanaik, Reinhard, Drago, Krawczyk, Kim, Madmad; Insufficient contrast: Durand, Mertens, Khan18, Liang, Khan20. (TIFF) [file pone.0340777.s032.tif]

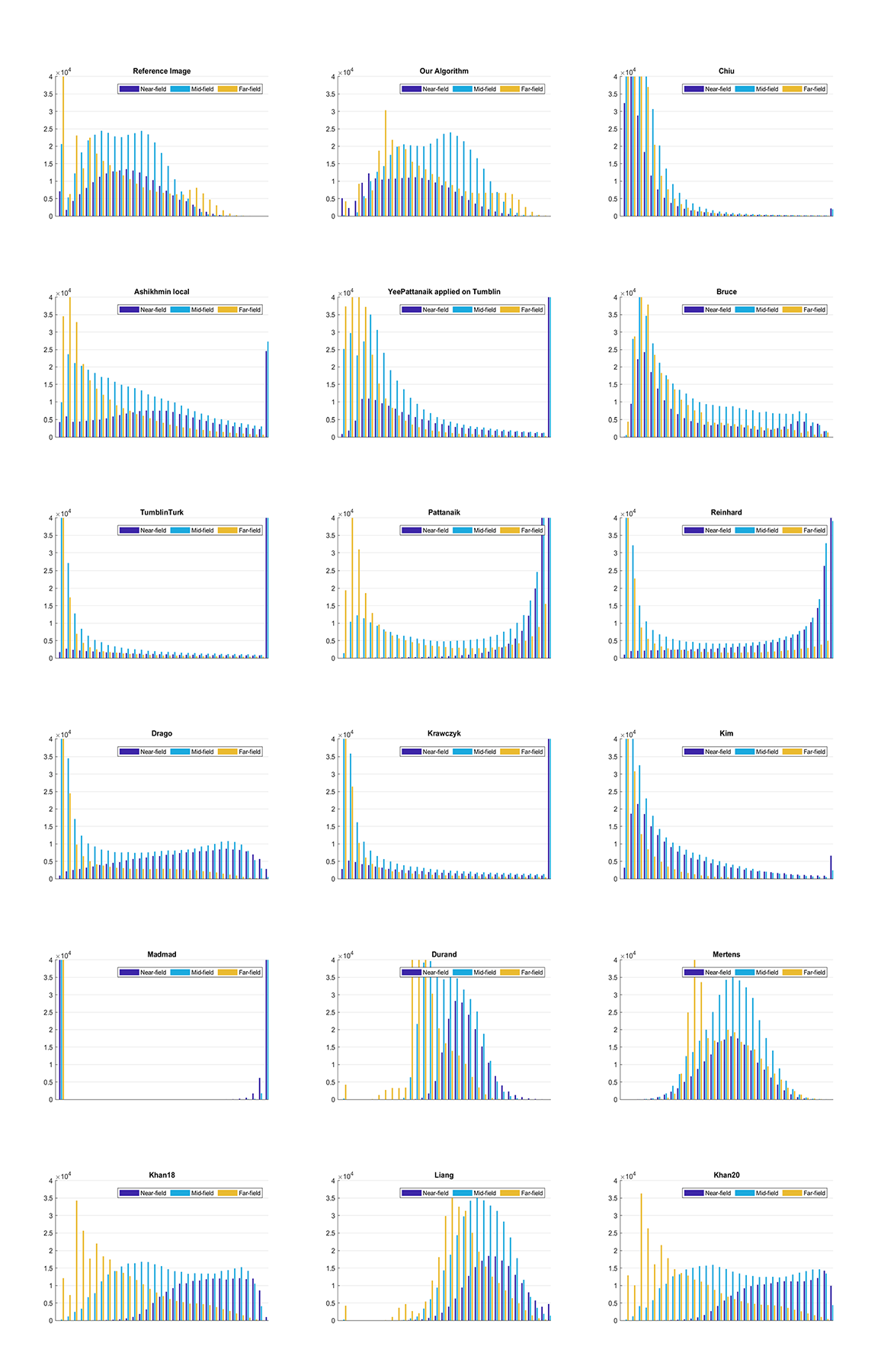

Supplement: S30 Fig — Left to right, top to bottom: image from VOLUSON Expert 22, our proposed method, Artifacts: Chiu, Ashikhmin local, YeePattanaik applied on Tumblin, Bruce; Overexcessive contrast: TumblinTurk, Pattanaik, Reinhard, Drago, Krawczyk, Kim, Madmad; Insufficient contrast: Durand, Mertens, Khan18, Liang, Khan20. (TIFF) [file pone.0340777.s033.tif]

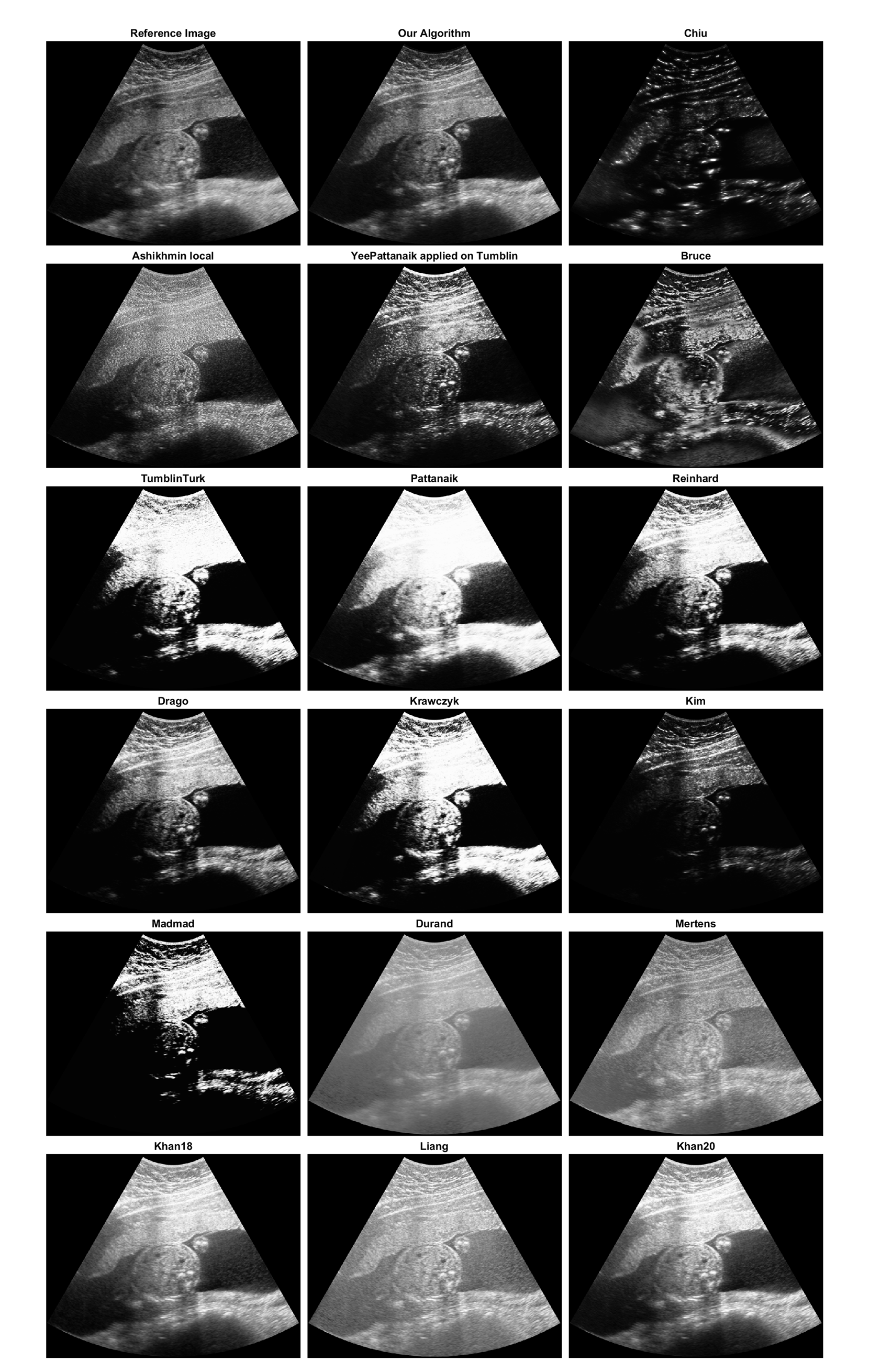

Supplement: S31 Fig — Left to right, top to bottom: image from VOLUSON Expert 22, our proposed method, Artifacts: Chiu, Ashikhmin local, YeePattanaik applied on Tumblin, Bruce; Overexcessive contrast: TumblinTurk, Pattanaik, Reinhard, Drago, Krawczyk, Kim, Madmad; Insufficient contrast: Durand, Mertens, Khan18, Liang, Khan20. (TIFF) [file pone.0340777.s034.tif]

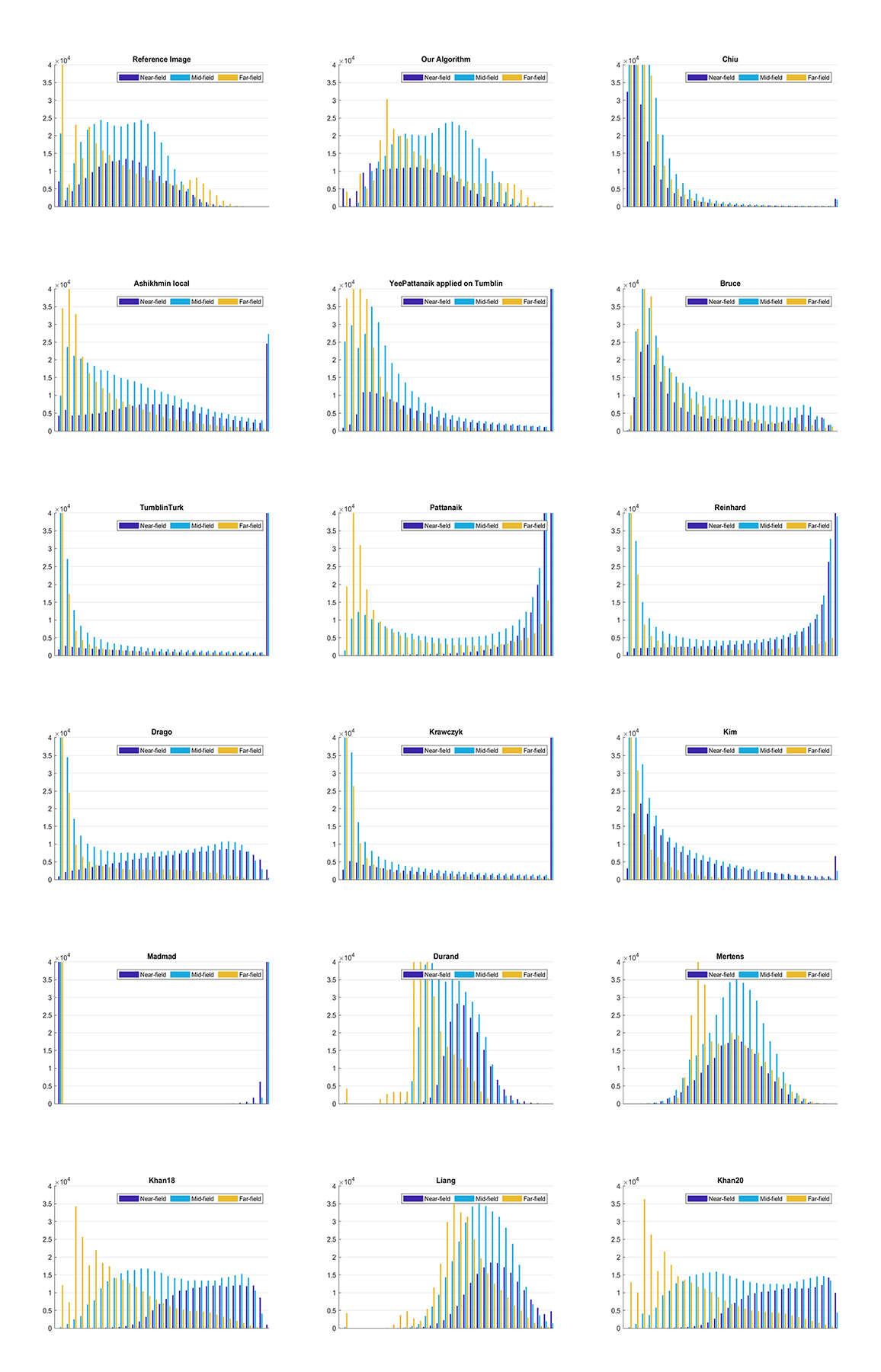

Supplement: S32 Fig — Left to right, top to bottom: image from VOLUSON Expert 22, our proposed method, Artifacts: Chiu, Ashikhmin local, YeePattanaik applied on Tumblin, Bruce; Overexcessive contrast: TumblinTurk, Pattanaik, Reinhard, Drago, Krawczyk, Kim, Madmad; Insufficient contrast: Durand, Mertens, Khan18, Liang, Khan20. (TIFF) [file pone.0340777.s035.tif]

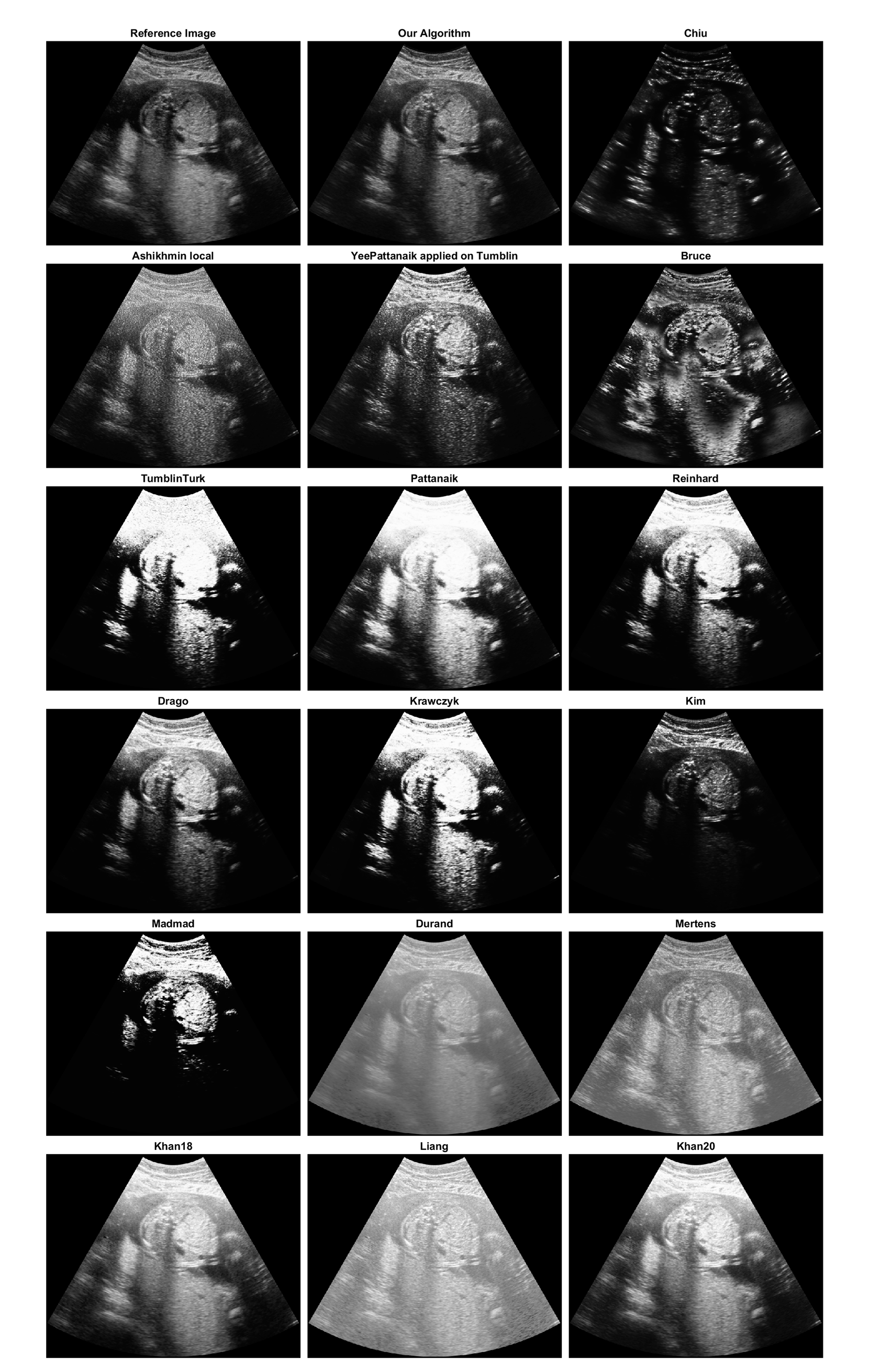

Supplement: S33 Fig — Left to right, top to bottom: image from VOLUSON Expert 22, our proposed method, Artifacts: Chiu, Ashikhmin local, YeePattanaik applied on Tumblin, Bruce; Overexcessive contrast: TumblinTurk, Pattanaik, Reinhard, Drago, Krawczyk, Kim, Madmad; Insufficient contrast: Durand, Mertens, Khan18, Liang, Khan20. (TIFF) [file pone.0340777.s036.tif]

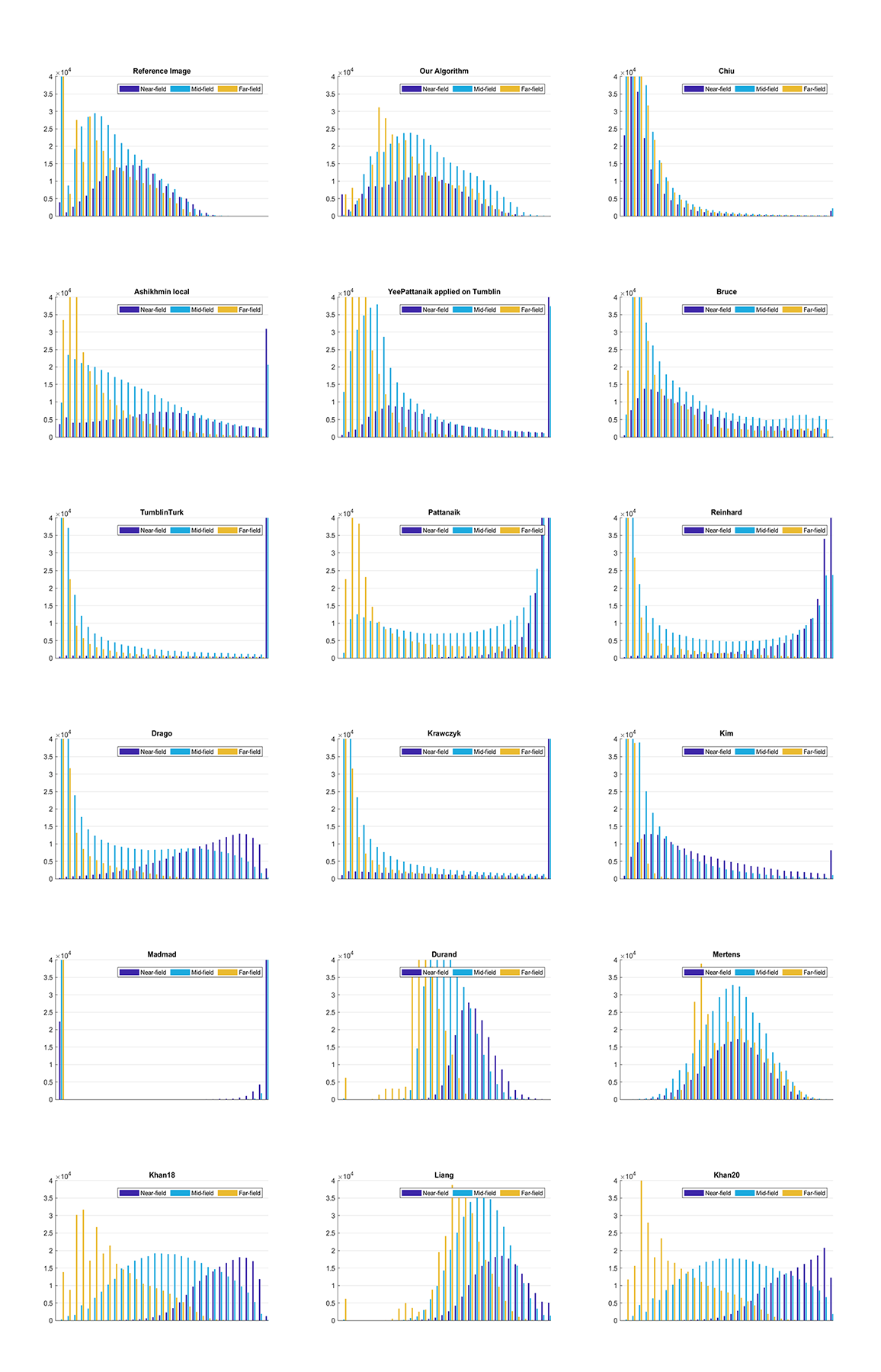

Supplement: S34 Fig — Left to right, top to bottom: image from VOLUSON Expert 22, our proposed method, Artifacts: Chiu, Ashikhmin local, YeePattanaik applied on Tumblin, Bruce; Overexcessive contrast: TumblinTurk, Pattanaik, Reinhard, Drago, Krawczyk, Kim, Madmad; Insufficient contrast: Durand, Mertens, Khan18, Liang, Khan20. (TIFF) [file pone.0340777.s037.tif]

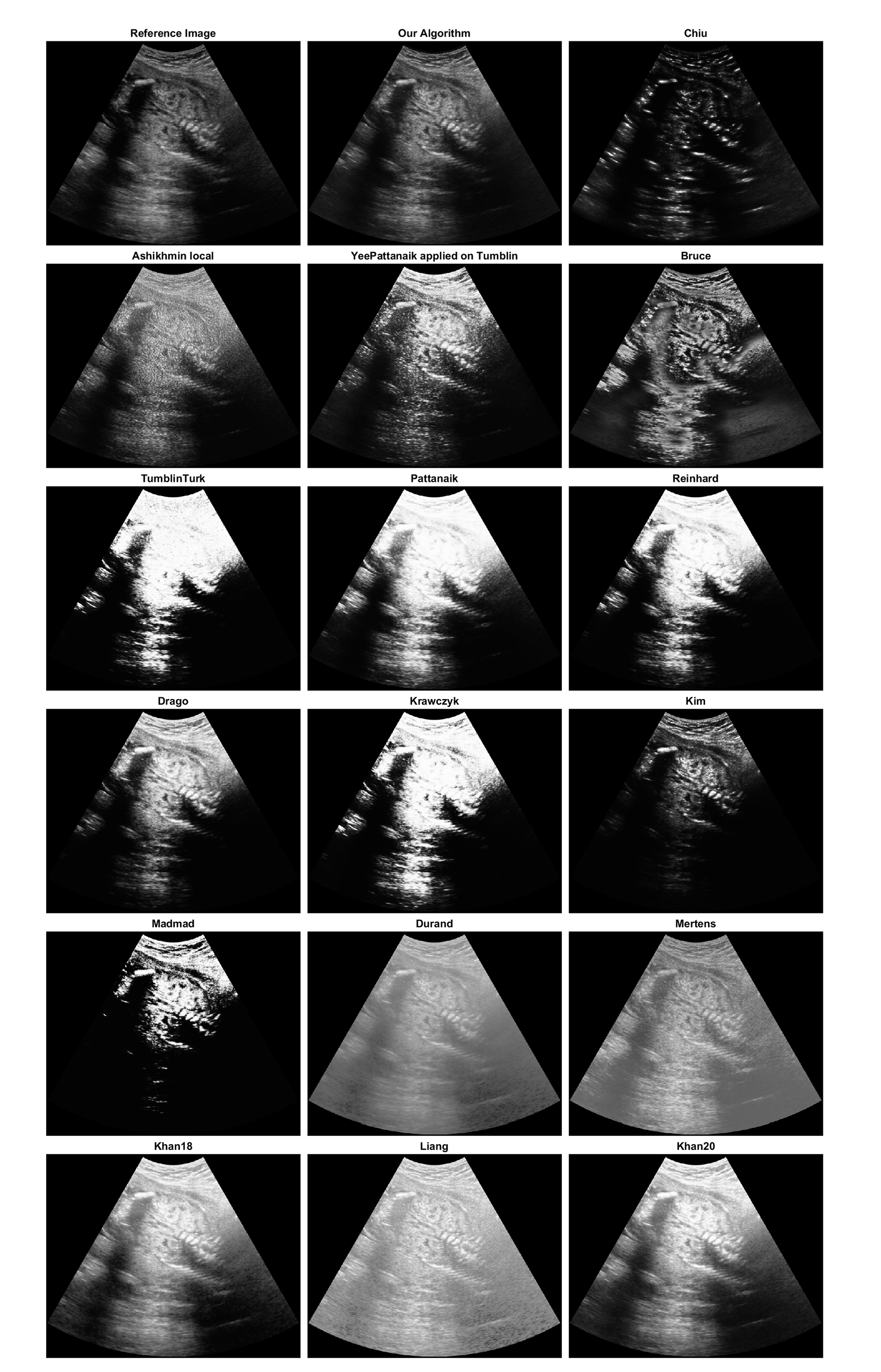

Supplement: S35 Fig — Left to right, top to bottom: image from VOLUSON Expert 22, our proposed method, Artifacts: Chiu, Ashikhmin local, YeePattanaik applied on Tumblin, Bruce; Overexcessive contrast: TumblinTurk, Pattanaik, Reinhard, Drago, Krawczyk, Kim, Madmad; Insufficient contrast: Durand, Mertens, Khan18, Liang, Khan20. (TIFF) [file pone.0340777.s038.tif]

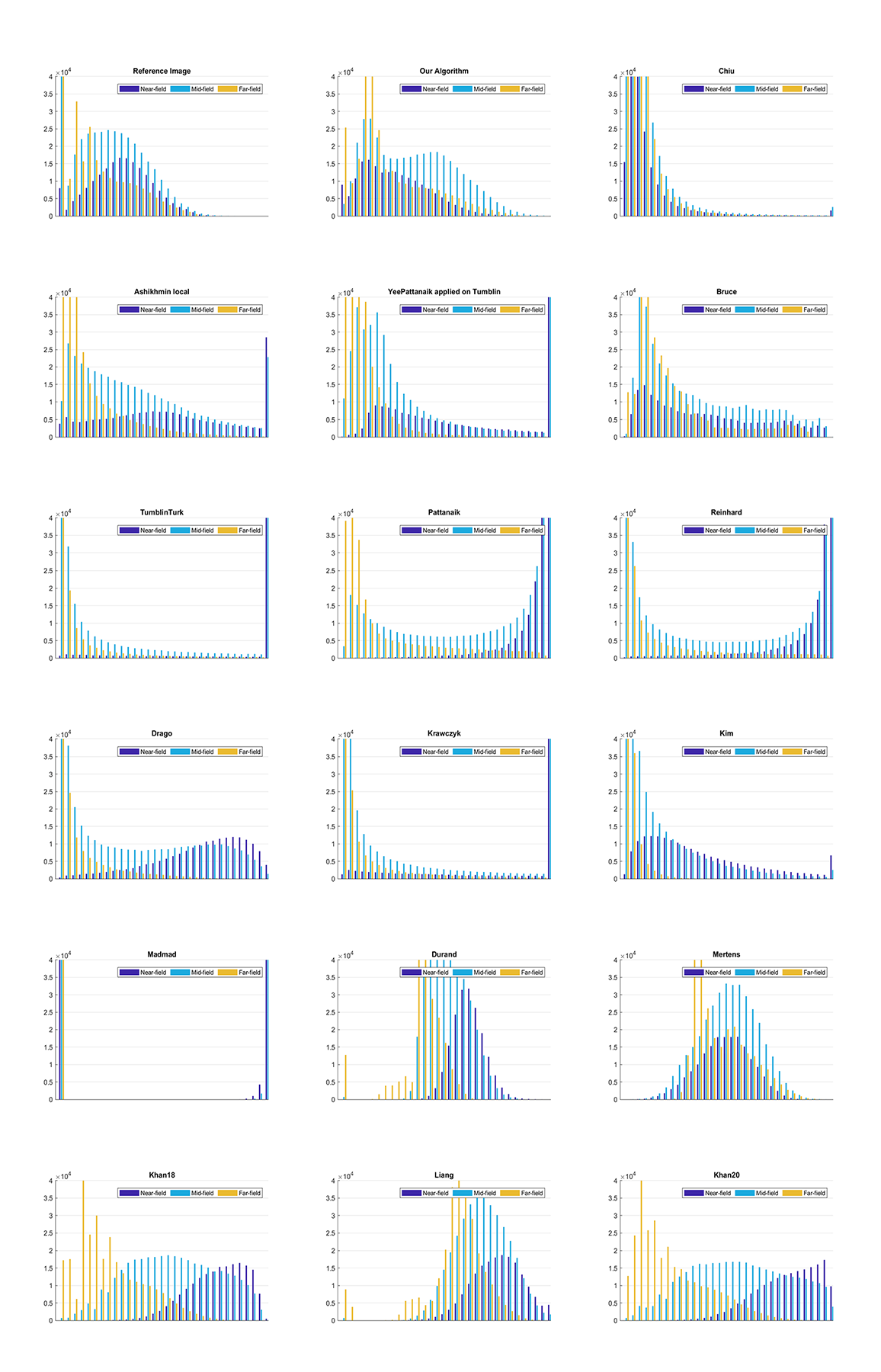

Supplement: S36 Fig — Left to right, top to bottom: image from VOLUSON Expert 22, our proposed method, Artifacts: Chiu, Ashikhmin local, YeePattanaik applied on Tumblin, Bruce; Overexcessive contrast: TumblinTurk, Pattanaik, Reinhard, Drago, Krawczyk, Kim, Madmad; Insufficient contrast: Durand, Mertens, Khan18, Liang, Khan20. (TIFF) [file pone.0340777.s039.tif]

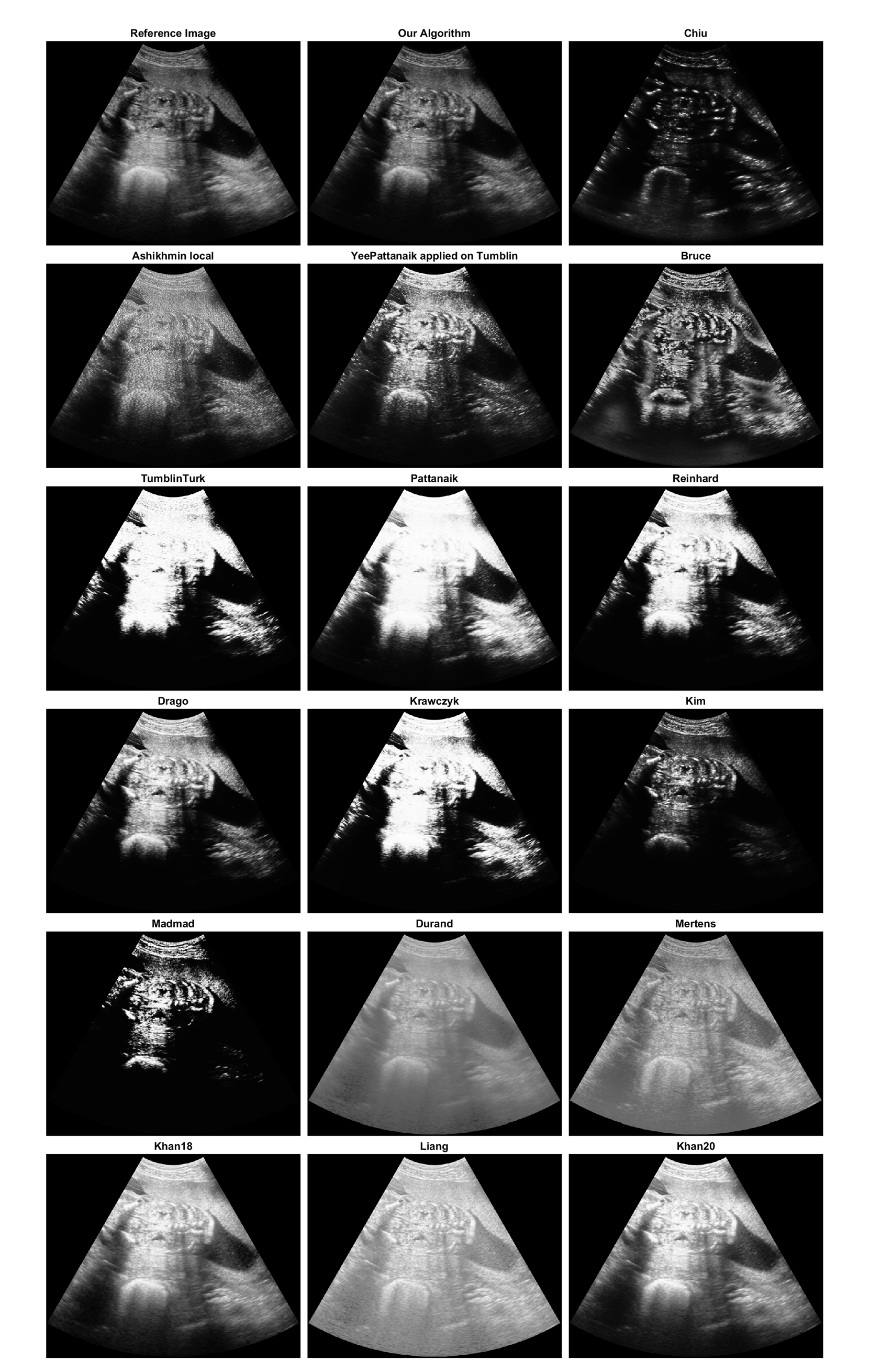

Supplement: S37 Fig — Left to right, top to bottom: image from VOLUSON Expert 22, our proposed method, Artifacts: Chiu, Ashikhmin local, YeePattanaik applied on Tumblin, Bruce; Overexcessive contrast: TumblinTurk, Pattanaik, Reinhard, Drago, Krawczyk, Kim, Madmad; Insufficient contrast: Durand, Mertens, Khan18, Liang, Khan20. (TIFF) [file pone.0340777.s040.tif]

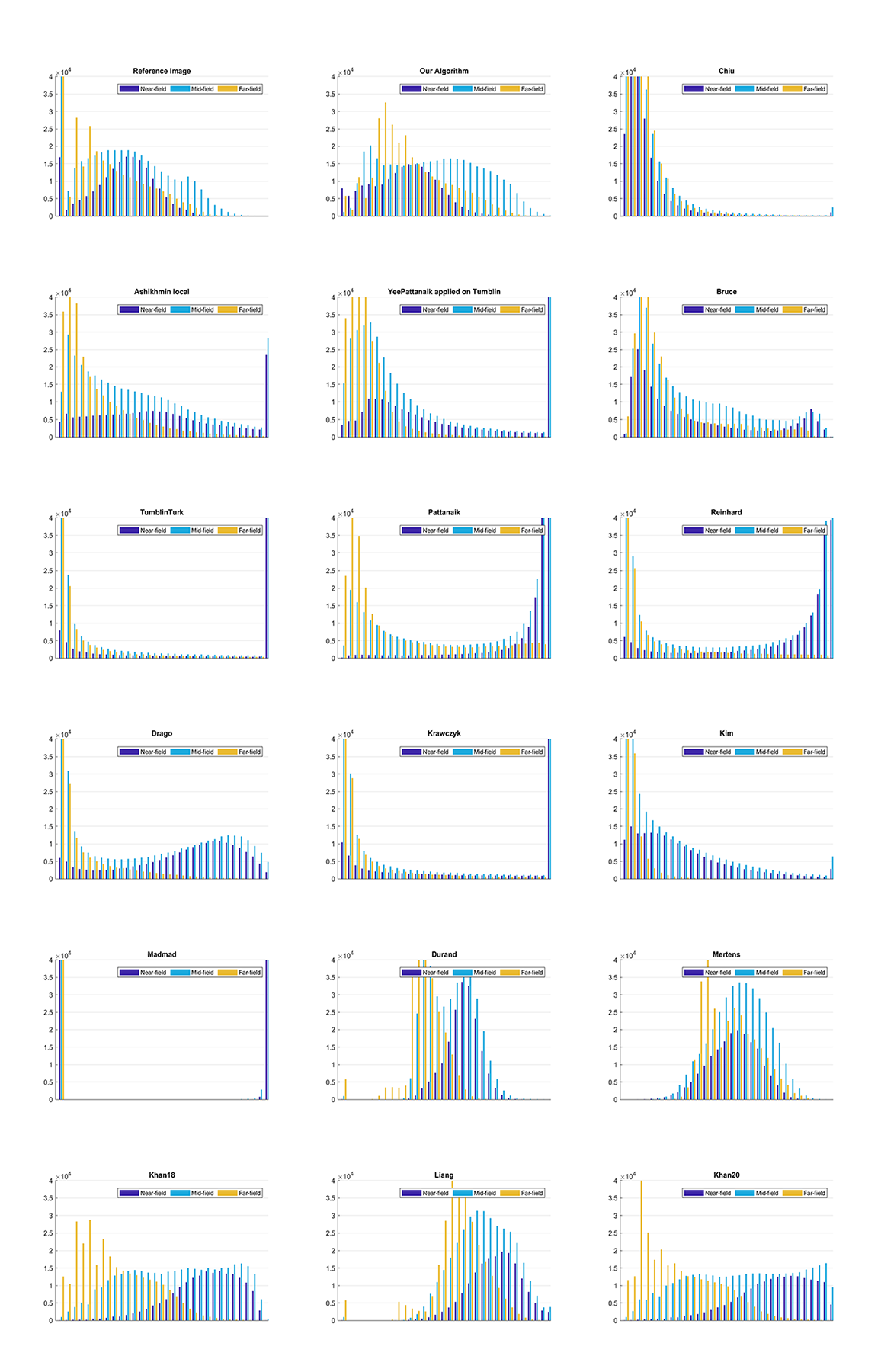

Supplement: S38 Fig — Left to right, top to bottom: image from VOLUSON Expert 22, our proposed method, Artifacts: Chiu, Ashikhmin local, YeePattanaik applied on Tumblin, Bruce; Overexcessive contrast: TumblinTurk, Pattanaik, Reinhard, Drago, Krawczyk, Kim, Madmad; Insufficient contrast: Durand, Mertens, Khan18, Liang, Khan20. (TIFF) [file pone.0340777.s041.tif]

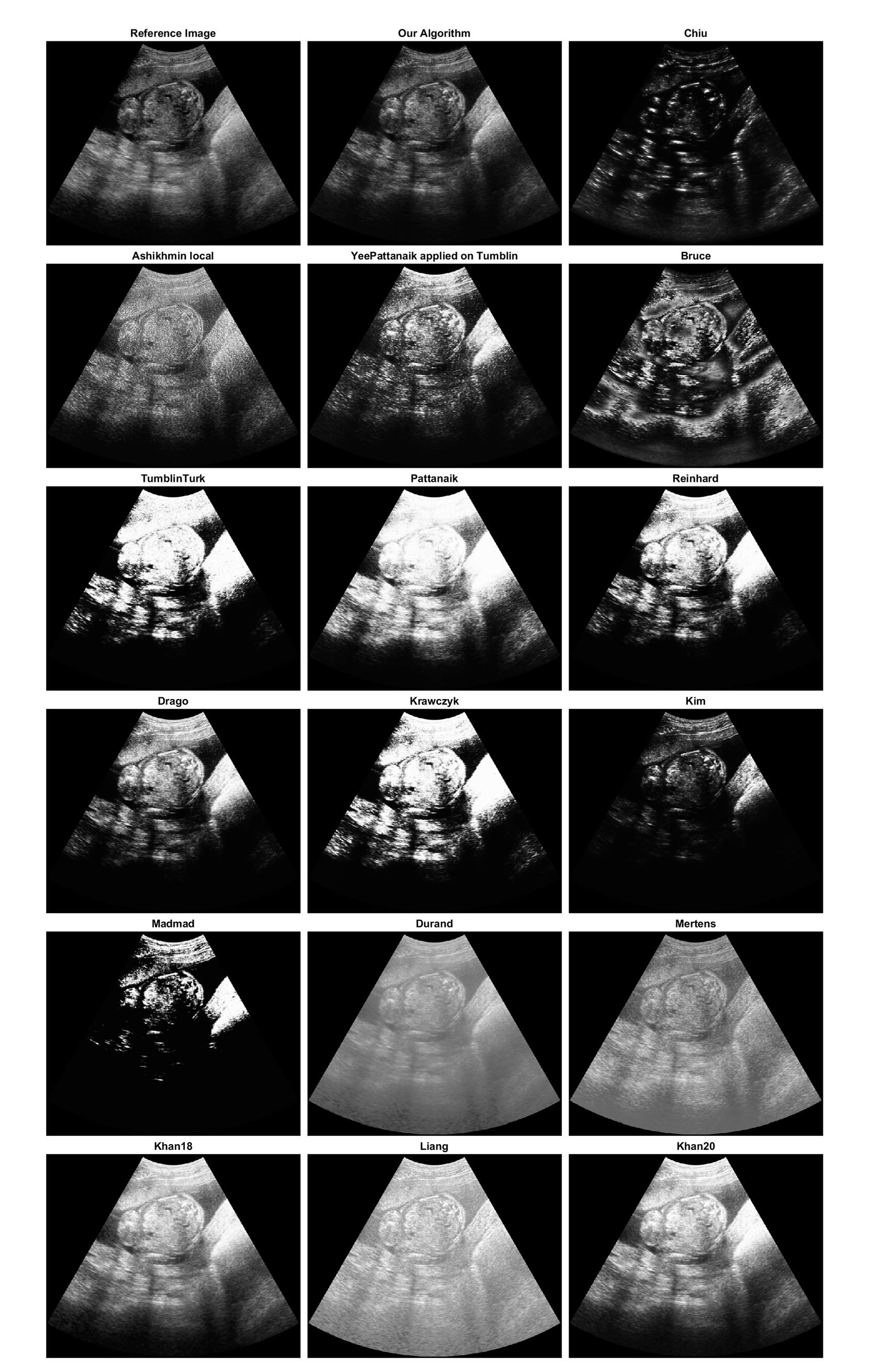

Supplement: S39 Fig — Left to right, top to bottom: image from VOLUSON Expert 22, our proposed method, Artifacts: Chiu, Ashikhmin local, YeePattanaik applied on Tumblin, Bruce; Overexcessive contrast: TumblinTurk, Pattanaik, Reinhard, Drago, Krawczyk, Kim, Madmad; Insufficient contrast: Durand, Mertens, Khan18, Liang, Khan20. (TIFF) [file pone.0340777.s042.tif]

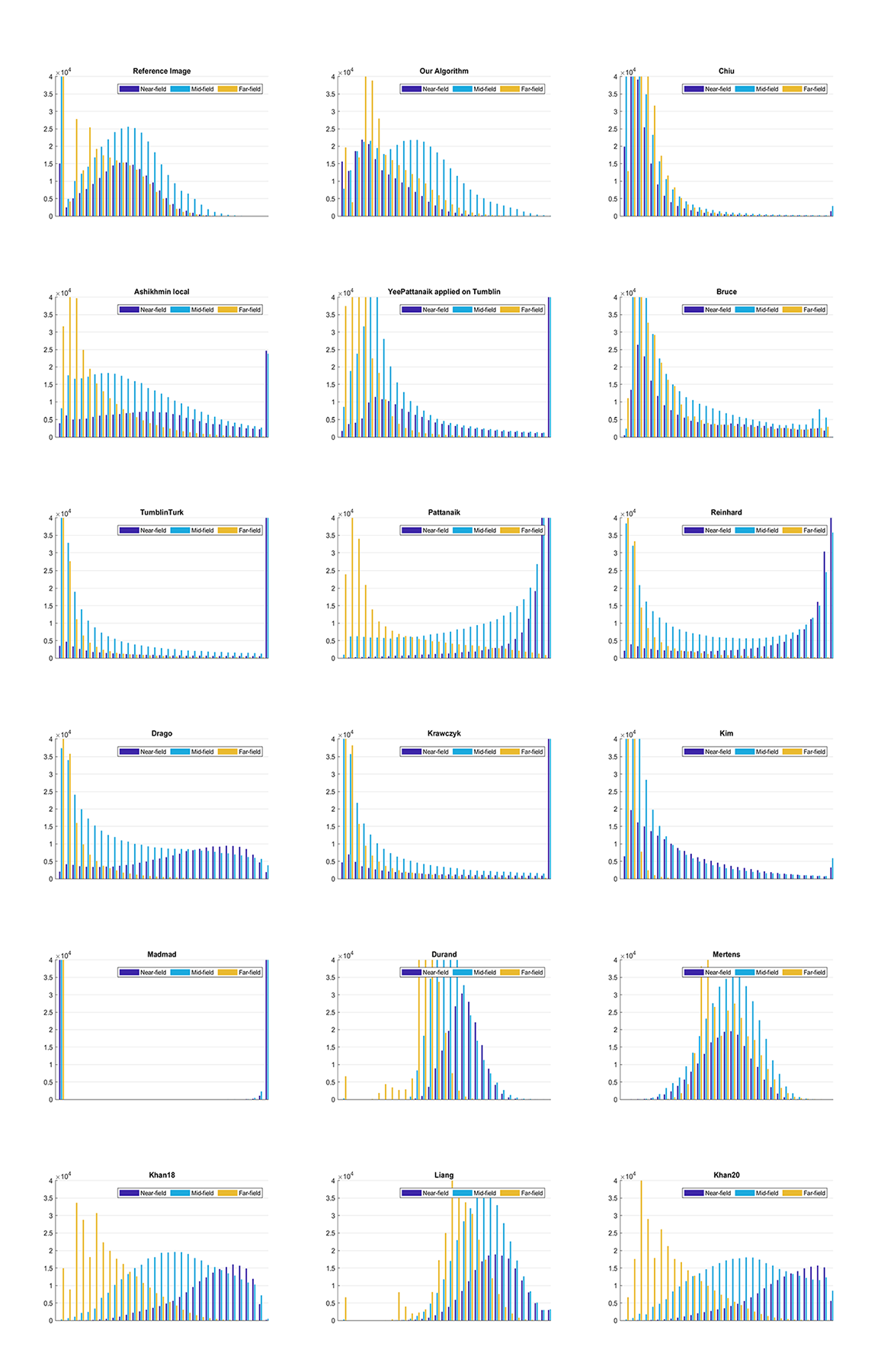

Supplement: S40 Fig — Left to right, top to bottom: image from VOLUSON Expert 22, our proposed method, Artifacts: Chiu, Ashikhmin local, YeePattanaik applied on Tumblin, Bruce; Overexcessive contrast: TumblinTurk, Pattanaik, Reinhard, Drago, Krawczyk, Kim, Madmad; Insufficient contrast: Durand, Mertens, Khan18, Liang, Khan20. (TIFF) [file pone.0340777.s043.tif]
